# Revision of the West Palaearctic *Polistes* Latreille, with the descriptions of two species – an integrative approach using morphology and DNA barcodes (Hymenoptera, Vespidae)

**DOI:** 10.3897/zookeys.713.11335

**Published:** 2017-11-02

**Authors:** Christian Schmid-Egger, Kees van Achterberg, Rainer Neumeyer, Jérôme Morinière, Stefan Schmidt

**Affiliations:** 1 Fischerstr. 1, 10317 Berlin, Germany; 2 Naturalis Biodiversity Center, P.O. 9517, 2300 RA Leiden, The Netherlands; 3 Probsteistrasse 89, 8051 Zürich, Switzerland; 4 SNSB-Zoologische Staatssammlung München, Münchhausenstr. 21, 81247 Munich, Germany

**Keywords:** Taxonomic revision, DNA barcoding, key to species, integrative taxonomy

## Abstract

The genus *Polistes* is revised for the West Palaearctic region based on morphology and DNA barcodes. The revision includes all known West Palaearctic species, raising the number of species in Europe to 14 and to 17 for the West Palaearctic realm. DNA barcodes were recovered from 15 species, 14 of which belong to the subgenus Polistes, and one, *P. wattii*, to the subgenus Gyrostoma. An integrative taxonomic approach combining morphology and molecular data (DNA barcoding) was employed to resolve longstanding taxonomic problems in this group. Two species, *P. austroccidentalis* van Achterberg & Neumeyer, **sp. n.** (= *P. semenowi* auctt.) from W and SW Europe and *P. maroccanus* Schmid-Egger, **sp. n.** from Morocco are described as new. *Polistes bucharensis* Erichson, 1849, and *P. foederatus* Kohl, 1898, were restored from synonymy. The following new synonyms are proposed: *P. sulcifer* Zimmermann, 1930, and Pseudopolistes sulcifer var. similator Zirngiebl, 1955, under *P. semenowi* Morawitz, 1889, **syn. n.**; *Polistes iranus* Guiglia, 1976, Polistes gallica var. ornata Weyrauch, 1938 and *Polistes gallicus muchei* Gusenleitner, 1976, under *P. bucharensis* Erichson, 1849, **syn. n.**; Polistes omissus var. ordubadensis Zirngiebl, 1955, and *P. hellenicus* Arens, 2011, under *Polistes mongolicus* du Buysson, 1911, **syn. n.** An illustrated key includes all species and additionally three species from the subgenera *Aphanilopterus* Meunier, 1888 and *Gyrostoma* Kirby, 1828 (including a Nearctic species recently introduced to Spain and two species occurring in Egypt, the Arabian Peninsula, and SW Asia). A phylogenetic analysis using Bayesian inference provides insights into phylogenetic relationships within the genus *Polistes*.

## Introduction

The paper wasp genus *Polistes* Latreille, 1802, is an important model group for behavioural and evolutionary studies (Tibbetts 2007, Hughes et al. 1993, Jandt et al. 2014). It includes many eusocial species that exhibit various forms of social organization. Moreover, the comparatively small colony size of *Polistes* species and their exposed nests facilitate both field observations and experiments (e.g., Cervo et al. 2008). Currently, over 220 species are recognized worldwide (Arens 2011, Buck et al. 2012, Nugroho et al. 2012), 11 of which occur in Europe (Neumeyer et al. 2014, Castro et al. 2013). Three of them, *P. atrimandibularis*, *P. austroccidentalis* sp. n. (= *P. semenowi* auctt.) and *P. semenowi (= P. sulcifer* Zimmermann, 1930), are social parasites (Cervo 2006, and references therein) and were formerly placed in a separate genus (or subgenus) *Sulcopolistes* Blüthgen, 1938 (Blüthgen 1961, Guiglia 1972) until Carpenter (1990) synonymized *Sulcopolistes* with *Polistes*. Subsequently, a phylogenetic analysis showed that the three socially parasitic species form the sister clade to a clade consisting of *P. dominula* (Christ, 1791) and *P. nimpha* (Christ, 1791) and that this clade is nested within the European *Polistes* species (Choudhary et al. 1994).

Blüthgen (1943) proposed the subgeneric name *Leptopolistes* for several non-parasitic European species, including the type species *P. associus* (Kohl, 1898) (Table 2). Males of these taxa share immediately narrowing temples (genae) in dorsal view (Blüthgen 1943, Guiglia 1972), giving the male head a characteristic shape. Currently, all European Polistes species are assigned to the subgenus Polistes (Carpenter 1996), although the species formerly included in *Leptopolistes* were still considered to be closely related (Carpenter 1997). In the present study, we are using “*P. gallicus* species group” to refer to the former subgenus Leptopolistes, and “*P. dominula* species group” for the remaining species, albeit with some changes from the traditional view (see Table 2).

The taxonomy of the *P. gallicus* species group has been notoriously difficult (see, for example Guiglia 1972, Arens 2011, Neumeyer et al. 2014, 2015). In the present study, an attempt was made to clarify the taxonomic status of all *Polistes* species of the West Palaearctic region, including a list of all available names of the genus, and to provide a key to species. Also, new names are proposed for two social parasitic species from NW Africa and SW Europe, one of them (*P. maroccanus* Schmid-Egger, sp. n.) new to science. Two other species (*P. bucharensis* Erichson, 1849, and *P. foederatus* Kohl, 1898) were restored from synonymy, thus raising the number of valid species in Europe to 14, and to 17 for the West Palaearctic region. Additionally, three species from other subgenera (an American species recently introduced into Spain and two southern species occurring in Egypt, Arabian Peninsula, and SW Asia) are included in the key.

The present study employs the concept of integrative taxonomy (Schlick-Steiner et al. 2010) and compares results from morphological examinations with results from DNA barcoding (see Schmidt et al. 2015 for further details). The morphological data are supplemented with published data (Neumeyer et al. 2014, 2015) and unpublished sources, the latter kindly provided from Aleksandar Ćetković (Belgrade) to Kees van Achterberg.

## Materials and methods

### Sampling

Specimens for DNA barcoding are primarily deposited in the collections of the Zoologische Staatssammlung München and the private collection of CSE. 264 specimens representing all West Palaearctic species of the subgenus Polistes and the subgenus Gyrostoma were processed, the latter being represented by the single species *P. wattii* Cameron, 1900. For DNA extraction, a single leg was removed from each specimen (for further details see Schmidt et al. 2015).

For the present study, a large number of specimens from several collections were morphologically examined. The taxonomic treatment of species is primarily based on the combined analysis of morphological and molecular data, and only those specimens that were analysed both, morphologically and genetically, are listed in the Suppl. material 1.

### DNA extraction and PCR

DNA extraction, PCR amplification, and sequencing were conducted at the Canadian Centre for DNA Barcoding (CCDB) using standardised high-throughput protocols (Ivanova et al. 2006, de Waard et al. 2008), available online under www.ccdb.ca/resources. The 658bp target region, starting from the 5’ end of the mitochondrial cytochrome oxidase *c* (COI) gene, includes the DNA barcode region of the animal kingdom (Hebert et al. 2003). The DNA extracts are stored at the CCDB with aliquots being deposited at the “DNA Storage” facility at the ZSM (see www.zsm.mwn.de/einrichtungen/dna-storage/?lang=en). Specimens that were successfully sequenced are listed under Supporting Information, with sequence lengths and the number of unresolved bases. Detailed specimen and sequence data are accessible in BOLD as a single citable dataset (dx.doi.org/10.5883/DS-WPPOLIST). The sequences are also available on GenBank (for accession numbers see Suppl. material 1).

### Molecular analyses

Sequence divergence statistics were calculated using the Kimura two-parameter model of sequence evolution (Kimura 1980). Sequences shorter than 500bp were excluded from the distance calculations and the phylogenetic analysis. The BIN is assigned by the BOLD system and represents a globally unique identifier for a cluster of sequences that has shown to correspond closely to a biological species (Ratnasingham & Hebert 2013), including Hymenoptera (Schmidt et al. 2015, 2016) and other insects (Hausmann et al. 2011, Raupach et al. 2014, Hendrich et al. 2015, Mutanen et al. 2016). Genetic distances and summary indices were calculated using analytical tools in BOLD and are given as mean and maximum pairwise distances for intraspecific variation, and as minimum pairwise distances for interspecific variation (see Table 1).

**Table 1.** Barcoding statistics of *Polistes* species with distribution, Barcode Index Number (BIN), number of specimens (n), mean intraspecific distance, maximum intraspecific distance, nearest neighbour species, and distance to nearest neighbour species. Distances are based on the Kimura 2P model, country codes follow ISO 3166-1.

*Polistes* species	BIN	n	Country	Mean intraspecific distance	Maximum intraspecific distance	Nearest *Polistes* species	Distance to NN
*P. albellus*	BOLD:AAN3553	12	CH, DE, KZ	0.03	0.16	*P. bischoffi*	2.31
*P. associus*	BOLD:ACG2253	7	HR, IT	0	0	*P. nimpha*	4.82
*P. atrimandibularis*	BOLD:AAN4297	2	IT	0	0	*P. semenowi*	1.7
*P. austroccidentalis*	BOLD:ACG1677	5	CH, FR, MA	0.58	0.95	*P. atrimandibularis*	2.18
*P. biglumis*	BOLD:AAN3552	21	DE, IT	0.57	1.17	*P. bischoffi*	2.48
*P. bischoffi*	BOLD:ACG2292	11	CH, FR, HR, SP	0.14	0.32	*P. albellus*	2.31
*P. bucharensis*	BOLD:ACM7975	9	AZ, CY	1.03	2.03	*P. dominula*	3.29
	BOLD:ACR2719	1	AZ				
	BOLD:ACY7463	3	GR				
*P. dominula*	BOLD:AAA9495	14	DE, FR	2.45	5.42	*P. bucharensis*	3.29
	BOLD:AAB7105	37	AZ, CH, DE, GR, HR, IT				
	BOLD:ACR3974	10	MA				
*P. foederatus*	BOLD:ACG2291	21	AZ, GR, HR, IT, TR	0.49	1.29	*P. biglumis*	2.88
*P. gallicus*	BOLD:AAN3302	20	HR, IT, PT, SP	0.28	1.09	*P. biglumis*	2.49
	NONE	2	AZ, DE				
*P. maroccanus*	BOLD:ACR4397	1	MA	N/A	0	*P. atrimandibularis*	3.78
*P. mongolicus*	BOLD:AAN3303	51	AZ, CY, GR, HR, IT, TR	0.88	2.04	*P. biglumis*	2.99
*P. nimpha*	BOLD:AAL0103	18	DE, GR, IT	2.2	6.28	*P. dominula*	3.56
	BOLD:ACC1661	4	DE				
*P. semenowi*	BOLD:ACG1290	6	CH, IT	0.28	0.65	*P. atrimandibularis*	1.7
*P. wattii*	BOLD:AAE1384	4	UAE	0	0	*P. gallicus*	12.84

### Phylogenetic analysis

The COI sequence data were submitted to a phylogenetic analysis using MrBayes version 3.2 (Ronquist and Huelsenbeck 2003) after alignment using the BOLD Aligner. Duplicates of identical sequences were removed from the dataset so that each haplotype was represented by a single sequence. The analysis employed separate, unlinked substitution models for codon positions of the COI sequence fragment. Two independent runs with one cold and three heated chains were run for five million generations after which the average standard deviation of split frequencies reached values lower than 0.01. The resulting tree was rooted using two basal outgroup taxa (Peters et al. 2017), including one representative of the Eumeninae (*Eumenes pedunculatus*) and one species of Masarinae (*Celonites abbreviatus* (Villers)).

### Acronyms of depositories and other institutions

**CSCF** Centre Suisse de Cartographie de la Faune, Neuchâtel, Switzerland

**CSE** Christian Schmid-Egger, Berlin, Germany

**CvA** Kees van Achterberg, Leiden, The Netherlands

**ETHZ** Entomologische Sammlung der Eidgenössischen Technischen Hochschule Zürich, Switzerland

**HNHM** Hungarian Natural History Museum, Budapest, Hungary

**LSL** Linnean Society of London, London, United Kingdom

**MFNB** Museum für Naturkunde, Berlin, Germany

**MNHN** Muséum national d’Histoire naturelle, Paris, France

**MSNG** MMuseo di storia naturale Giacomo Doria, Genova, Italy

**MSNM** Museo civico di storia naturale, Milano, Italy

**MSNV** MMuseo di Storia Naturale di Venezia, Italy

**MZL** Musée cantonal de zoologie, Lausanne, Switzerland

**NHMW** Naturhistorisches Museum Wien, Austria

**NMBE** Naturhistorisches Museum der Burgergemeinde Bern, Switzerland

**OLL** Oberösterreichisches Landesmuseum Linz, Austria.

**RMNH** Naturalis Biodiversity Center, Leiden, the Netherlands

**RN** Rainer Neumeyer, Zürich, Switzerland

**ZISP** Zoological Institute of the Russian Academy of Sciences, St Petersburg, Russia

**ZSM** Zoologische Staatssammlung München, Munich, Germany

**ZMUZ** Zoologisches Museum der Universität Zürich, Switzerland

### Abbreviation used in the key and descriptions

**POL** Postocellar diameter, the distance between the lateral ocelli.

## Results

### Key to West Palaearctic species of the genus *Polistes* Latreille

Regional terms of geographic distribution are abbreviated as Central (C), Southern (S), Eastern (E), Northern (N) and West (W). Southwest Asia includes Turkey, Iran, Israel and adjacent countries. Temperate Asia refers to Asia north of 37° northern latitude (approximative only, species may occur south of 37° north, e.g. in mountain regions).

**Table:** 

1	Parastigma elongate and long compared to length of vein 1-SR of fore wing (a); second and third metasomal tergites with brown or blackish curved lines (b); [mesopleuron only sparsely punctate medially]; subgenus Gyrostoma	**2**
	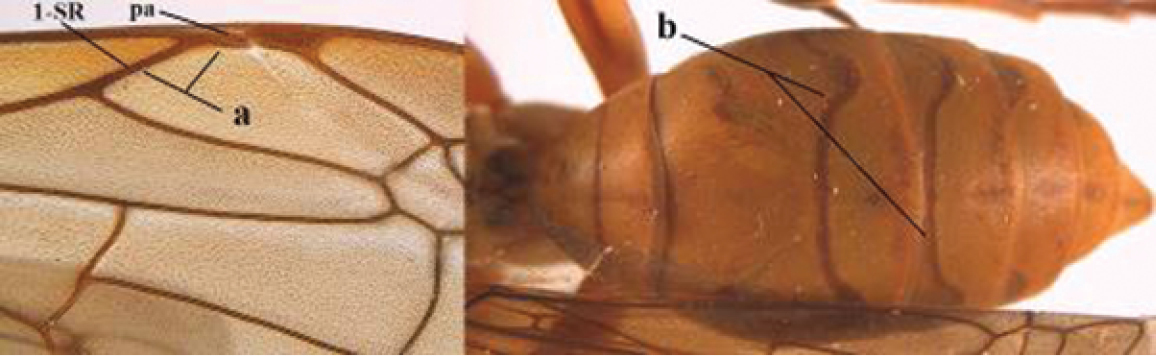	
–	Parastigma short, hardly or not longer than wide and short compared to length of vein 1-SR of fore wing (aa); second and third metasomal tergites without curved lines, often with a black pattern (bb)	**3**
	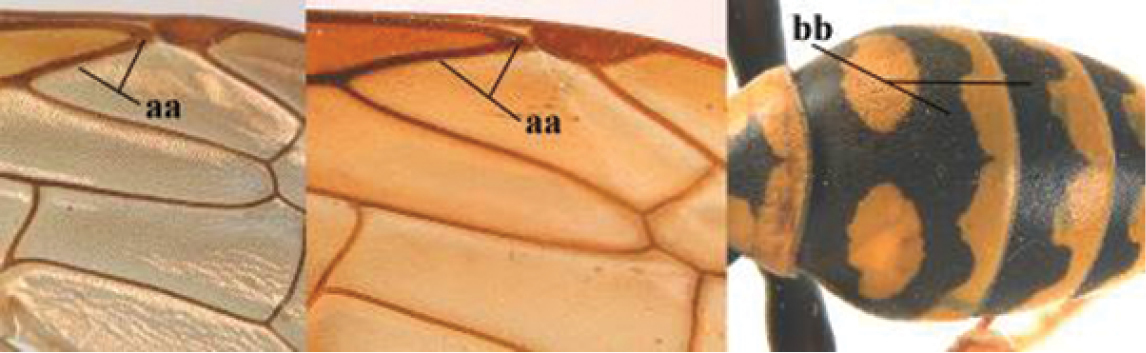	
2	Female: Dorsal part of epistomal (frontoclypeal) suture blackish or dark brown (a); length of fore wing 15–28 mm. Male: Clypeus evenly convex (b); lateral tubercles on each side of apex of last sternite subtriangular and wider basally (c), its terminal apophyses long and spatulate apically (d); Palaearctic distribution: Egypt, Oman, Iran, Afghanistan	***P. olivaceus* (DeGeer, 1773)**
	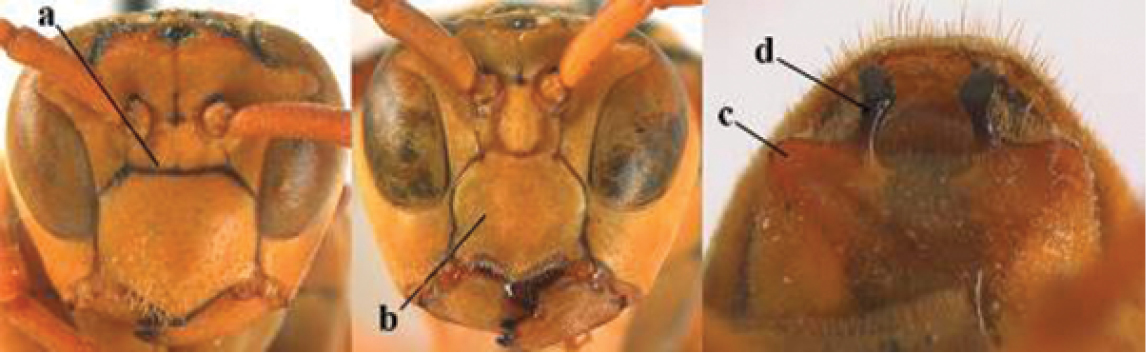	
–	Female: Dorsal part of epistomal suture yellowish (aa); length of fore wing 11–17 mm. Male: Clypeus with impression (bb); lateral tubercles of last sternite more cylindrical and narrower basally (cc), its terminal apophyses long and pointed apically (dd); Palaearctic distribution: Afghanistan, Iran, Iraq, Saudi Arabia, United Arab Emirates, Oman, China	***P. wattii* Cameron, 1900**
	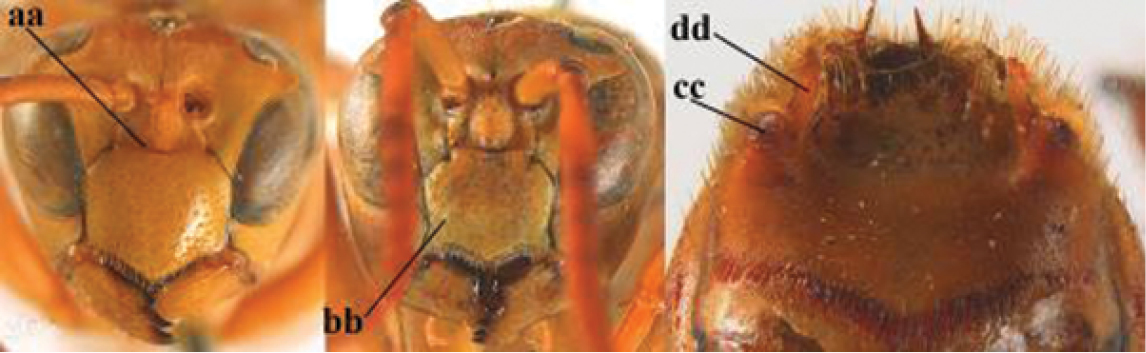	
3	Body brown with yellow pattern (a); mesopleuron indistinctly sculptured (b, c); recently introduced to northern Spain, native to South and Central America, Caribbean islands and southern U.S.A. (subgenus Aphanilopterus)	***P. major* Palisot de Beauvois, 1818**
	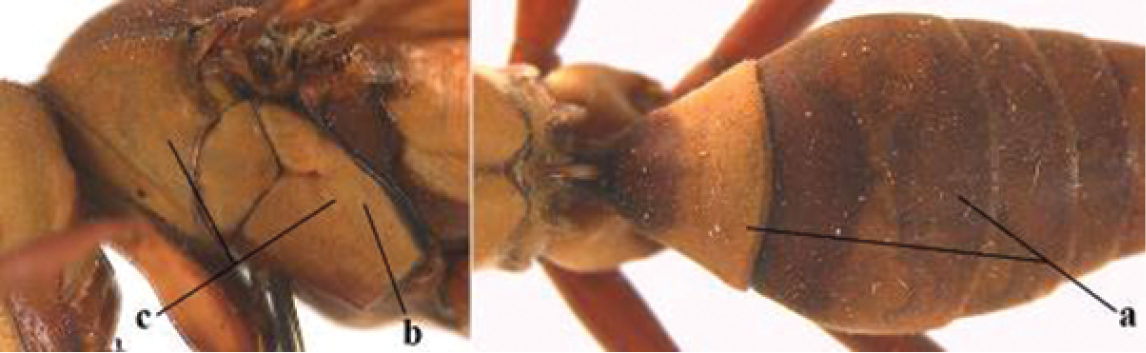	
–	Body black with yellow pattern (aa); mesopleuron with dense irregular microstriae (bb, cc) (subgenus Polistes)	**4**
	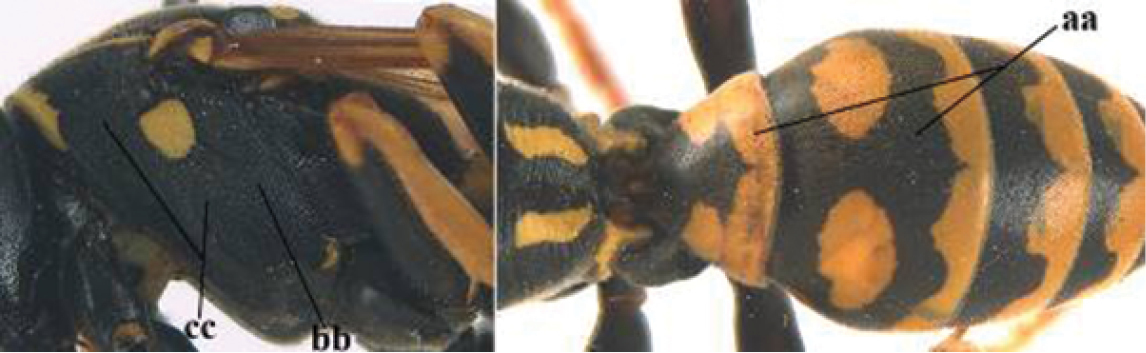	
4	Antenna with 12 segments (a); metasoma with 6 visible tergites and sternites (b); face and/or clypeus with yellow and black pattern (c); females	**5**
	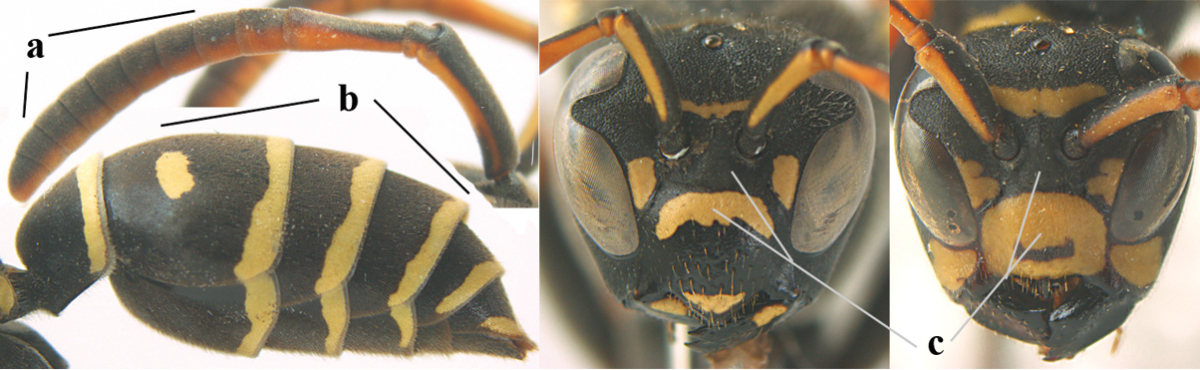	
–	Antenna with 13 segments (aa); metasoma with 7 visible tergites and sternites (bb); face and/or clypeus yellow (cc; but more or less blackish pattern present in *P. atrimandibularis* ccc); males	**21**
	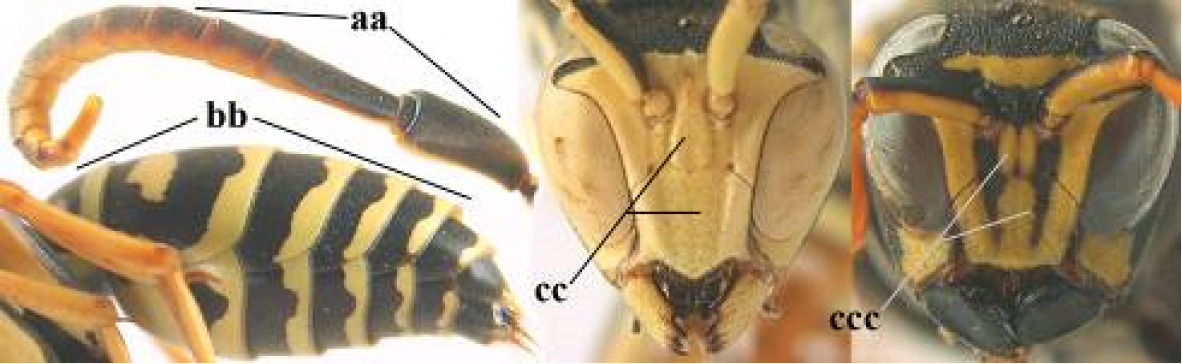	
5	Mandible very stout and its outer face more or less depressed (a); ventral border of clypeus strongly convex (b) and medially acute (c); socio-parasitic species	**6**
	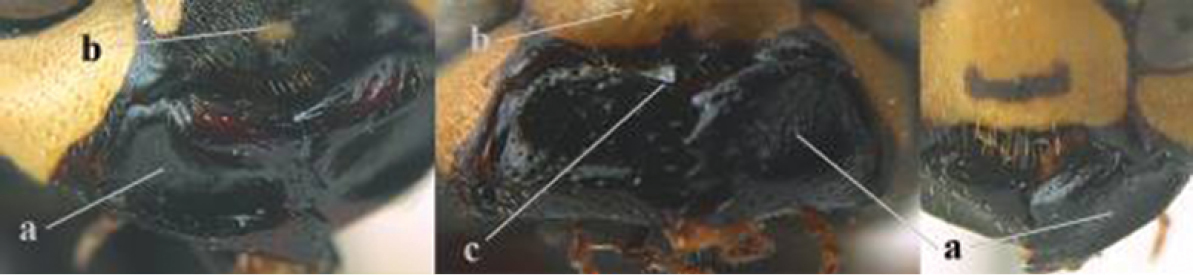	
–	Mandible comparatively slender and its outer face weakly convex or flat (aa); ventral border of clypeus flat (bb) and medially truncate or slightly concave (cc); social species	**9**
	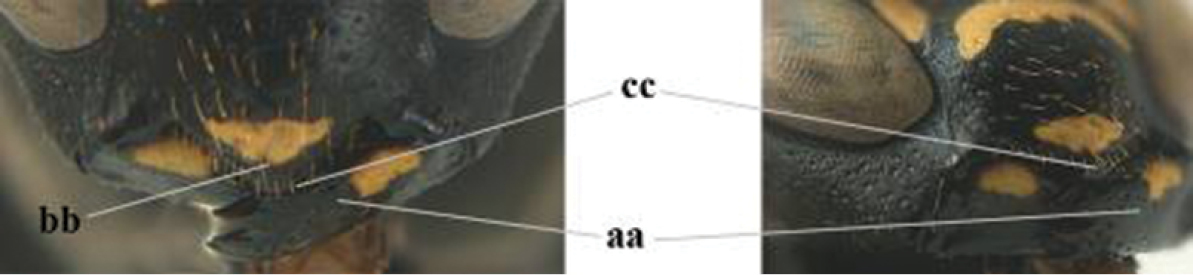	
6	Basal half of mandible distinctly angulate (a) and flattened (b); clypeus gradually depressed ventrally (c); [yellow area along inner eye margin connected to yellow bar above antennal sockets; clypeus largely punctate ventrally and with fine pubescence as in medial area (c)]; SE and southern C Europe, C Asia [= *P. sulcifer* Zimmermann, 1930]	***P. semenowi* Morawitz, 1889**
	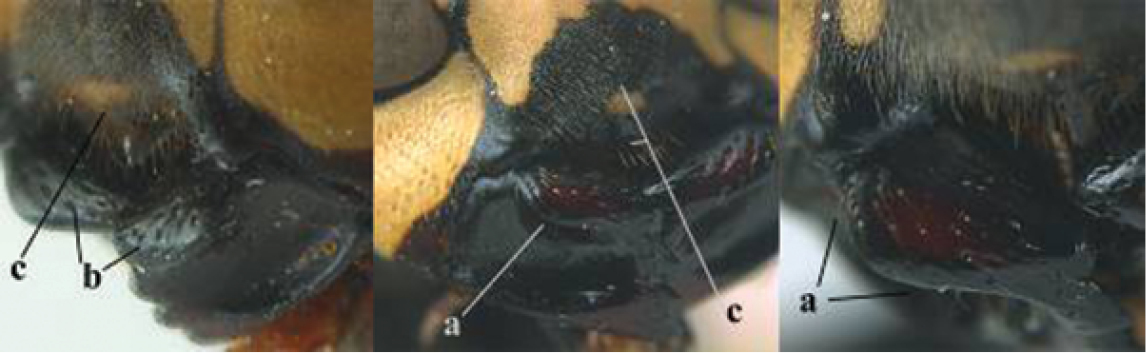	
–	Basal half of mandible gradually curved (aa) and convex (bb); clypeus abruptly depressed ventrally (cc)	**7**
	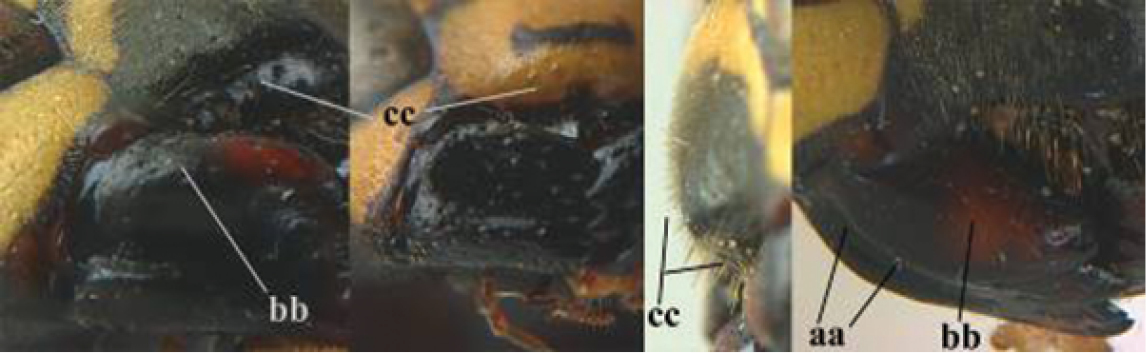	
7	Dorsal ridge of mandible wide, convex and distinctly elevated above middle of mandible (a, b); fine pubescence of clypeus conspicuous and comparatively long (b, c); yellow area along inner eye margin usually connected with yellow bar above antennal sockets (d); SW and southern C Europe, N Africa; [= *P. semenowi* auctt.]	***P. austroccidentalis* van Achterberg & Neumeyer, sp. n.**
	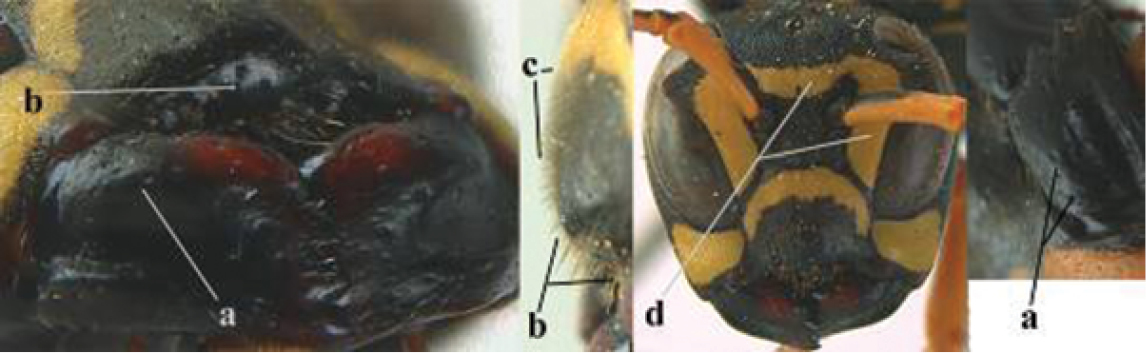	
–	Dorsal ridge of mandible narrow, slightly convex and hardly elevated above middle of mandible (aa); fine pubescence of clypeus inconspicuous and short (bb); yellow area along inner eye margin separated from yellow bar above antennal sockets (cc)	**8**
	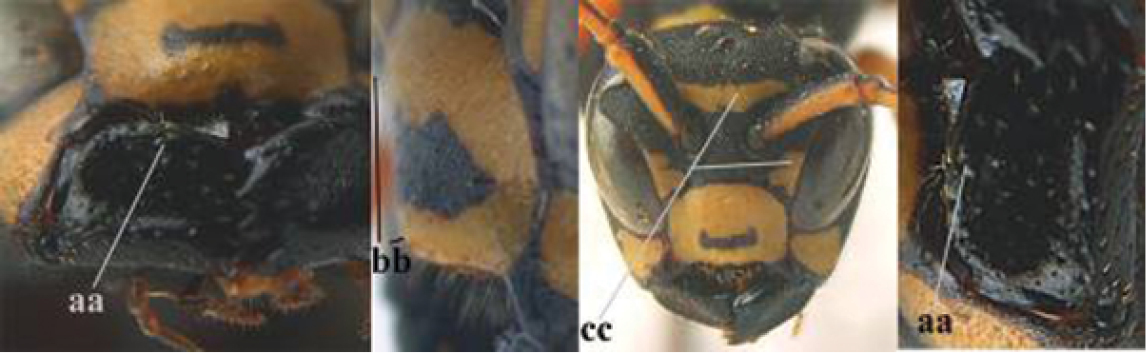	
8	Clypeus 0.7 times as long as wide (a); lower ridge of mandible 0.4 times as wide as mandible (b); depression of mandible with rather dull surface (c); area above lower edge of clypeus (below dark spot) dull and straight (d); S and southern C Europe to W Asia	***P. atrimandibularis* Zimmermann, 1930**
	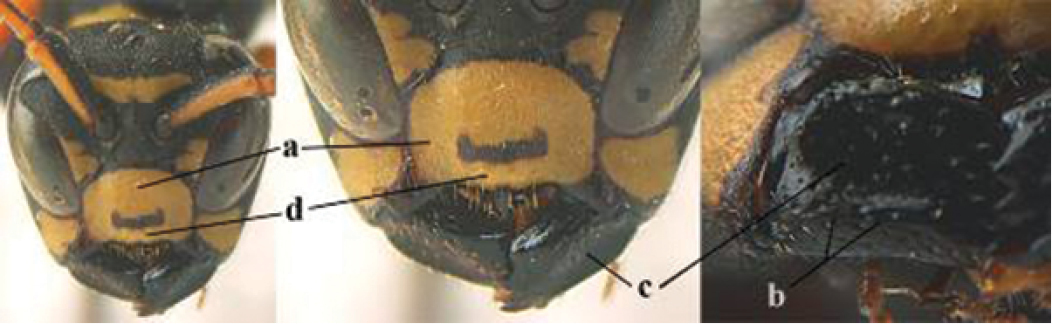	
–	Clypeus 0.8–0.9 times as long as wide (aa); lower ridge of mandible 0.2 times as wide as mandible (bb); depression of mandible with shiny surface (cc); area above lower edge of clypeus (below dark spot) shiny and distinctly convex (dd); Morocco	***P. maroccanus* Schmid-Egger, sp. n.**
	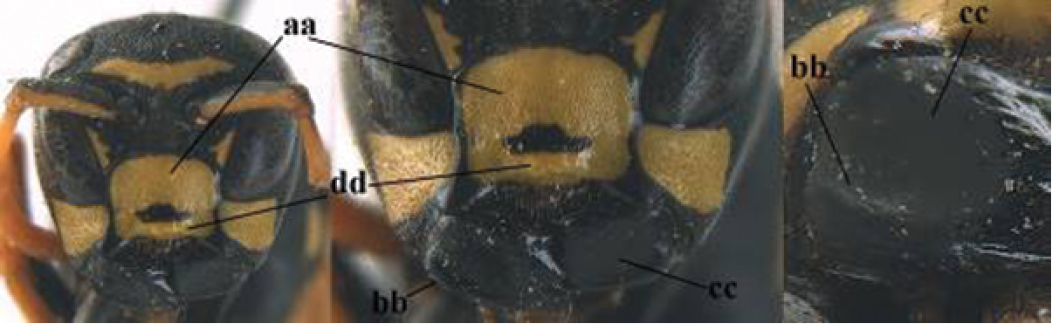	
9	Malar space mainly yellow and connected to yellow ventral part of temple (a); mandible usually black (b), **if** mandible with yellow patch then total yellow area smaller than yellow area of malar space and temple; clypeus somewhat wider than high medially (c); hind coxa black dorsally (d); *dominula*-group	**10**
	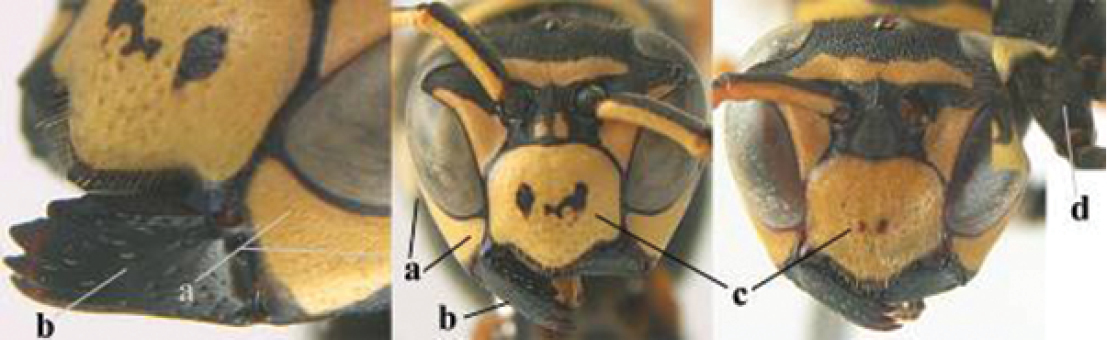	
–	Malar space black as ventral part of temple (aa); **if** rarely with small yellow patch then total yellow area of mandible larger than that of yellow patch of malar space and usually not connected to ventral yellow part of temple; mandible with yellow patch (bb); clypeus about as wide as high medially (cc); dorsal colour of hind coxa variable (dd, d); *gallicus*-group	**16**
	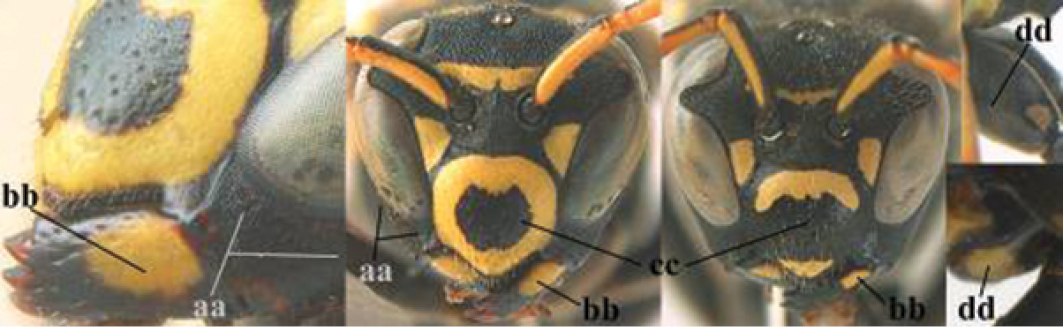	
10	Specimens from Europe (excluding Crete) and NW Africa	**11**
–	Specimens from Asia (including Crete and Cyprus)	**13**
11	Transverse yellow band of pronotum narrower medio-laterally than dorso-laterally near junction with oblique yellow stripes of pronotum (a), rarely narrowed dorso-laterally and slightly widened below it **and then** mesoscutum usually without yellow markings (d); scapus more or less dark brown or blackish baso-ventrally (b), apical half of hypopygium often entirely black or dark brown (c), sometimes with small yellow spot, rarely apical quarter of hypopygium yellow; mesoscutum usually without paired yellow spots (d) but sometimes minute, or medium-sized (dd); apical half of antenna more or less darkened dorsally (e); Europe and Palaearctic Asia	***P. nimpha* (Christ, 1791)**
	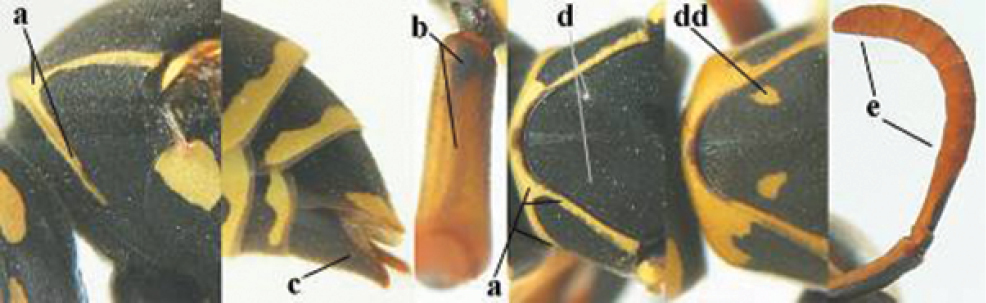	
–	Transverse yellow band of pronotum as wide medio-laterally as dorsolaterally or wider (aa; rarely narrowed medio-laterally and slightly widened below it) **and** mesoscutum usually with two yellow spots (dd); scapus entirely yellow baso-ventrally (bb), rarely slightly infuscate; colour of apical half of hypopygium variable, often entirely yellow or apically brownish (cc), rarely entirely black; mesoscutum with paired yellow spots usually medium-sized to large (dd), rarely minute or absent; apical half of antenna evenly orange or yellow dorsally (ee), but more or less infuscate in *P. associus*	**12**
	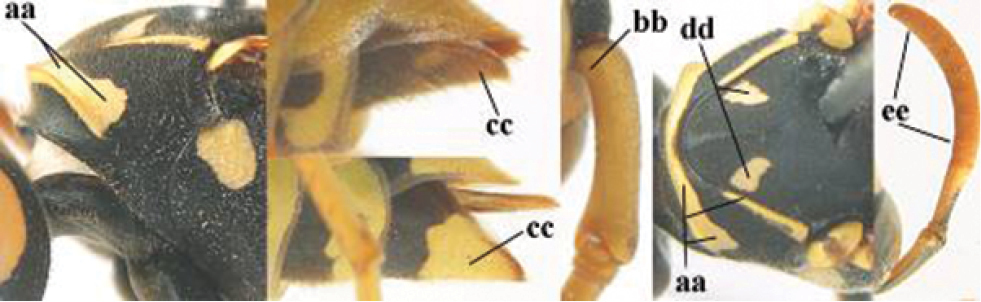	
12	Scapus slightly widened apically in dorsal view (a), if scapus intermediate then ocellar triangle acute anteriorly (b); change in sculpture between mesepisternum and epicnemium frequently gradual (c; = epicnemial ridge indistinct); apical half of antenna more or less brownish dorsally (d); apical half of hypopygium (= sternite VI) often largely black and brown or largely brown, slightly darker than apex of last tergite (e); border of black and yellow on outer side of middle and hind femora often sharp and often without orange intermediate area in Balkan populations (f); often smaller species; S Europe, W Asia	***P. associus* Kohl, 1898**
	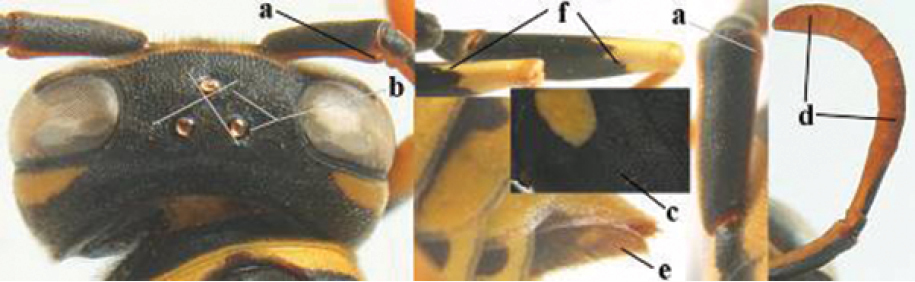	
–	Scapus distinctly widened apically in dorsal view (aa); ocellar triangle transverse (bb); change in sculpture between mesepisternum and epicnemium abrupt (cc; = epicnemial ridge distinct); apical half of antenna orange or yellow (dd); apical half of hypopygium yellow or largely so and as pale as apex of apical tergite (ee), rarely darker; border of black and yellow on outer side of middle and hind femora washed out, partly because of orange intermediate area (ff); somewhat larger species); Europe except N, NW Africa, W and C Turkey, Azerbaijan, probably also farther east in temperate Asia. Not in Crete, see *P. bucharensis* (couplet 14)	***P. dominula* (Christ, 1791)**
	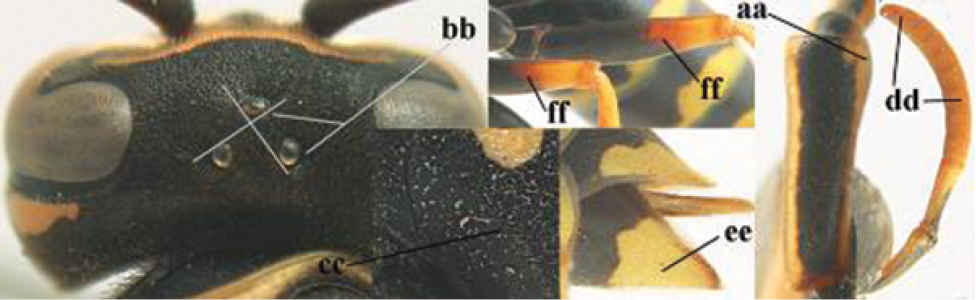	
13	Apical half of sternite VI yellow or largely so, and as yellow as apex of last tergite (a); ventral part of mesopleuron in general more coarsely sculptured than remaining parts (b); [mesoscutum usually with two yellow spots; apical half of antenna orange or yellow reddish]	**14**
	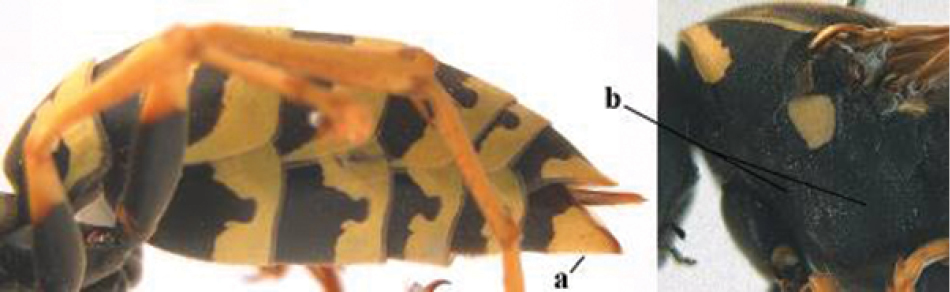	
–	Apical half of sternite VI largely black, **if** with reddish or yellowish apical part then pale part smaller than black part of tergite VI (aa), but especially in *P. associus* sometimes paler and not obviously smaller than black part of last tergite (aaa); mesopleuron ventrally evenly and finer sculptured (bb)	**15**
	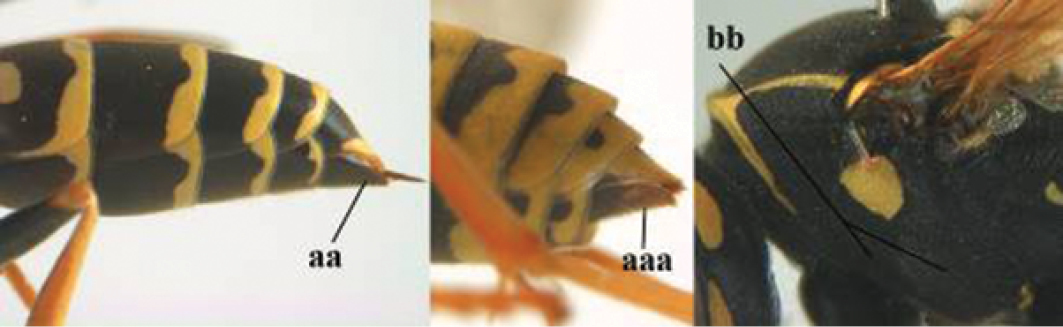	
14	Temple (gena) with wide yellow band, medially wider than half width of temple (a); clypeus yellow medially (b); propodeum and second tergite largely yellow (c); incision of eye mainly yellow (d); [frons slightly convex (about similar to area between antennal sockets and distinctly less so in *P. dominula*) and mainly yellow, extent of yellow area variable, sometimes reaching ocellar area, scapus often largely yellow dorsally]; W and C Asia, Cyprus, Egypt, Crete (specimens from Crete differ in colour pattern, see description below)	***P. bucharensis* Erichson, 1849**
	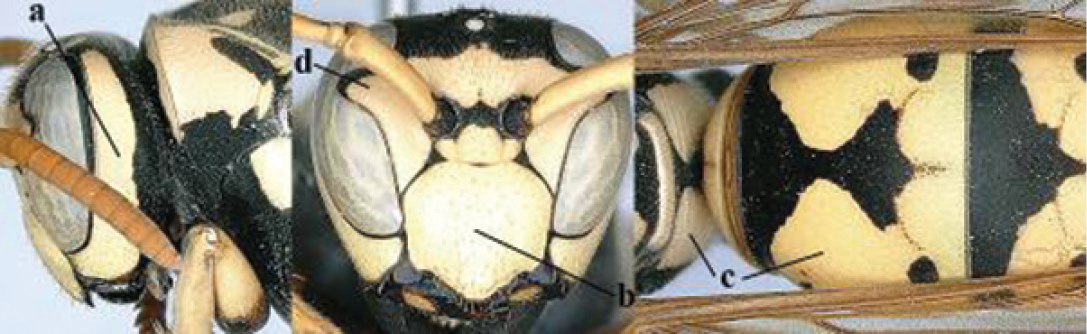	
–	Temple with separated yellow spots (aa), **if** connected then band medially narrower than half width of temple; clypeus with medial black spot, may lack in few specimens (bb); propodeum and second tergite largely black (cc); incision of eye mainly black (dd); [bristles of clypeus conspicuous]; W and C Turkey, Azerbaijan, probably also farther east in temperate Asia	***P. dominula* (Christ, 1791)**
	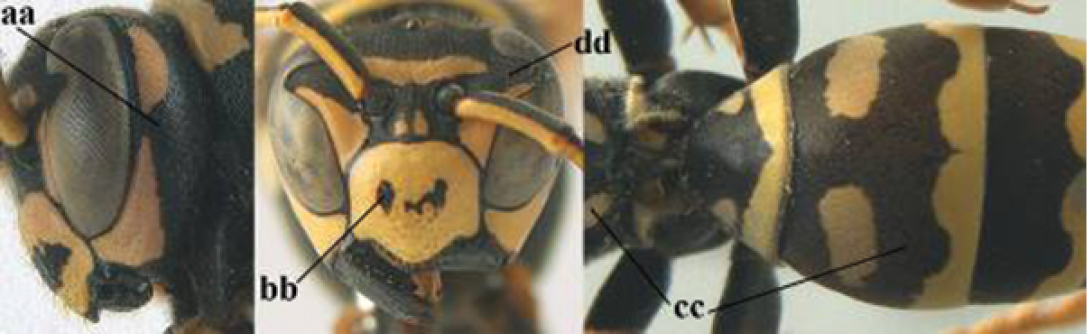	
15	Posterolateral yellow bands of pronotum usually not connected to anterior transverse band (a); anterior transverse yellow band of pronotum medio-laterally as wide as dorso-laterally or wider (b); mesoscutum with paired yellow spots (c); [clypeus with transverse black band; hypopygium all black, with apical yellow or reddish spot or entirely reddish; distinction from *P. nimpha* in SW Asia is not always possible, because *P. nimpha* is highly variable in this region; usually sculpture of ventral part of mesopleuron finer in *P. associus* than in *P. nimpha*]; southern Europe, western Asia	***P. associus* Kohl, 1898**
	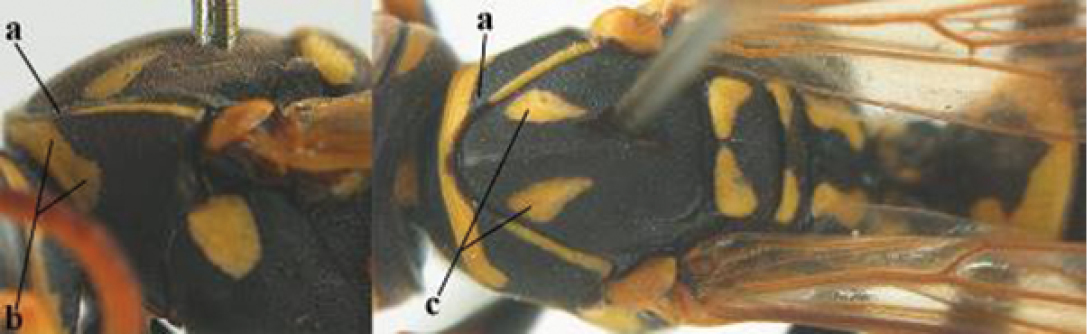	
–	Posterolateral yellow bands of pronotum usually connected to anterior transverse band (aa); anterior transverse yellow band of pronotum medio-laterally narrower than dorso-laterally (bb); mesoscutum sometimes lacking yellow spots (cc); [colour pattern is highly variable in this species, more so than in Europe]; Europe and Palaearctic Asia	***P. nimpha* (Christ, 1791)**
	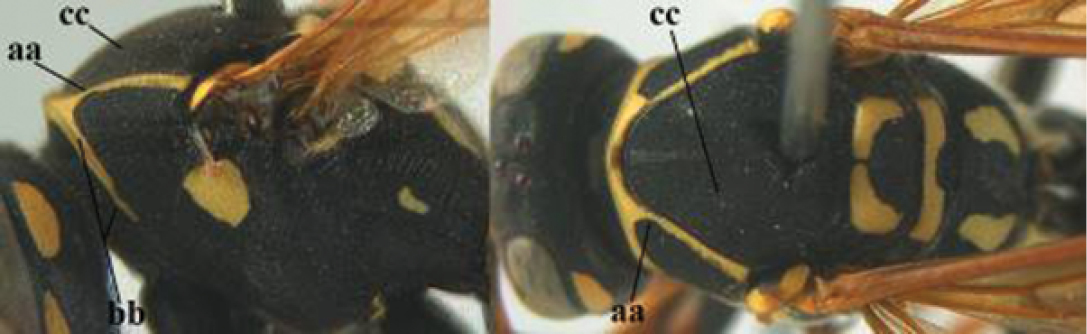	
16	Transverse yellow band of pronotum narrow (a); antenna largely black or blackish dorsally (b), similar to colour of frons; basal third of scapus more or less black or blackish ventrally (c); pubescence of pronotum and mesoscutum 0.7–0.9 times as long as diameter of anterior ocellus (d); yellow spots of propodeum narrow or absent, **if** present then usually narrower than minimum distance between spots (e); [malar space at least 0.87 times as long as POL]	**17**
	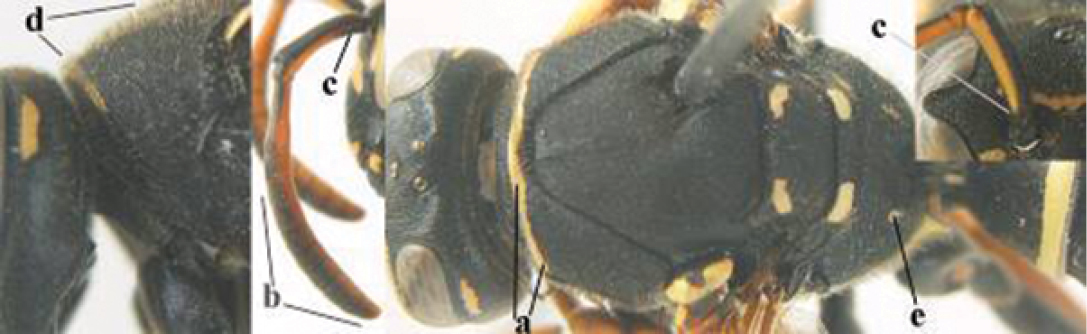	
-	Transverse yellow band of pronotum wider (aa); antenna orange brown or somewhat darkened dorsally (bb) and distinctly paler than frons; basal third of scapus yellow ventrally (cc), but darkened in most *P. foederatus*; pubescence of pronotum and mesoscutum about half as long as diameter of anterior ocellus (dd), but longer in *P. bischoffi* (ddd); yellow spots of propodeum wide and usually larger (ee), but sometimes smaller in *P. foederatus*; [malar space usually shorter than 0.87 times POL, except in *P. foederatus*]	**18**
	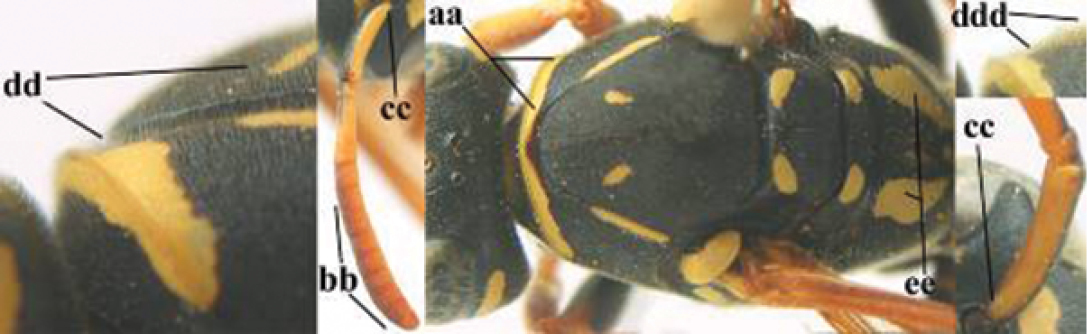	
17	Malar space 1.22–1.76 times as long as POL (a); change in sculpture and level between mesepisternum and epicnemium abrupt (b; = epicnemial ridge distinct); yellow lateral stripes of pronotum usually absent (c); tegula in most specimens with large dark medial spot, reaching outer margin (d); pubescence of mesoscutum on average 0.9 times as long as greatest diameter of anterior ocellus; Europe, temperate Asia	***P. biglumis* (Linnaeus, 1758)**
	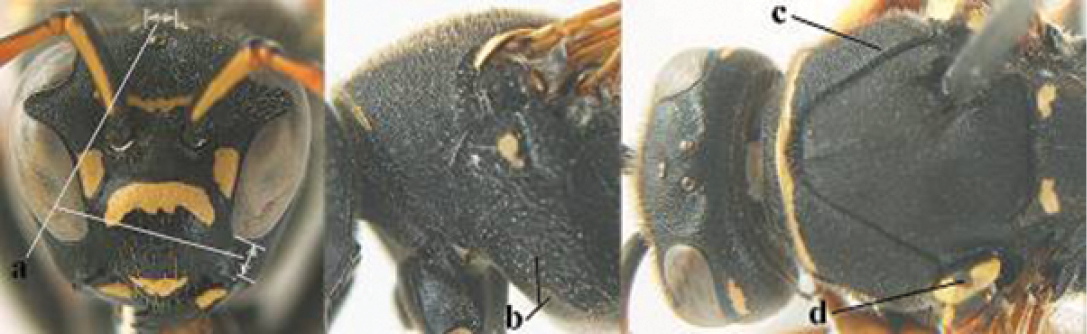	
–	Malar space 0.87–1.19 times as long as POL (aa); change in sculpture and level between mesepisternum and epicnemium often gradual (bb; = epicnemial ridge absent or obsolescent); yellow lateral stripes of pronotum present (cc; but absent in Mongolian specimens); tegula in most specimens entirely yellow, at most blackish subbasally (dd); pubescence of mesoscutum on average 0.7 times as long as greatest diameter of anterior ocellus; C Europe, temperate Asia	***P. albellus* Giordani Soika, 1976**
	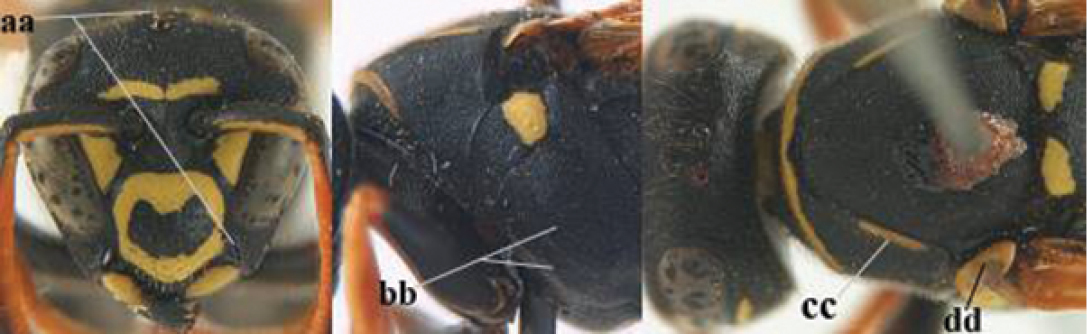	
18	Malar space 0.85–1.0 times as long as POL (a); distal half of antenna slightly darker dorsally (b); clypeus with central black patch, may lack in few specimens (c); metanotal and propodeal spots as in (d) [yellow dorsal spot of hind coxa large to absent; hind tibia dorsally with dark brown patch and at both, inner and outer side often with small preapical brown spot; yellow bands of sternites IV-V often medially interrupted; distinct difference in sculpture between mesepisternum and epicnemium (= epicnemial ridge distinct)]; N Italy to SE Europe, Turkey and Central Asia	***P. foederatus* Kohl, 1898**
	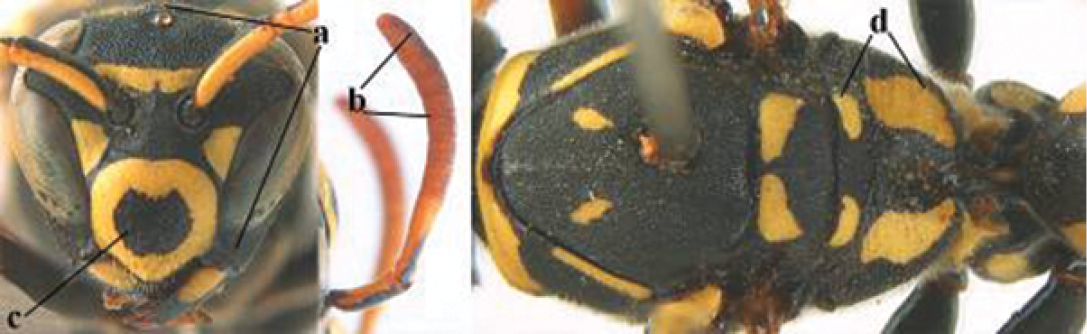	
–	Malar space at most 0.75 times as long as POL (aa); antenna as pale dorsally as ventrally (bb), but darker dorsally in some *P. bischoffi*; central dark patch on clypeus usually smaller and situated below middle of clypeus or absent (cc; except in *P. bischoffi*); propodeal and metanotal spots according to picture (dd)	**19**
	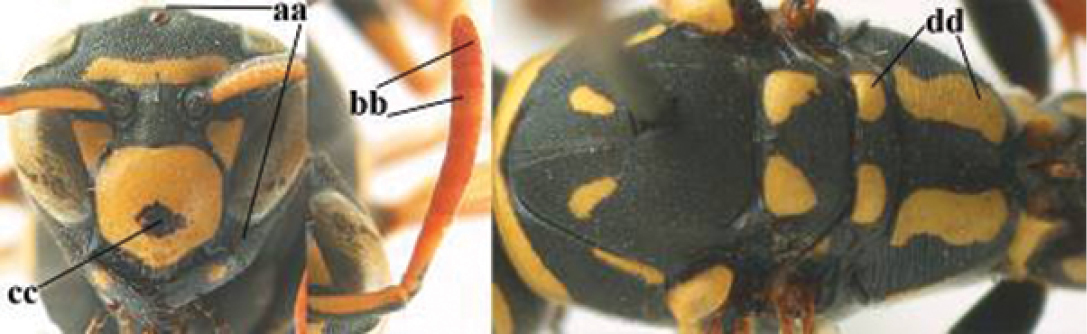	
19	Apical yellow band of sternite IV medially interrupted (a) or narrow; hind coxa black dorsally (b); clypeus often with a wide black transverse band or with more or less transverse trapezoid black patch, often close to or connected with lateral margins of clypeus and situated nearly halfway clypeus (c); change in sculpture between mesepisternum and epicnemium frequently gradual (d; = epicnemial ridge indistinct); southern C and S Europe	***P. bischoffi* Weyrauch, 1937**
	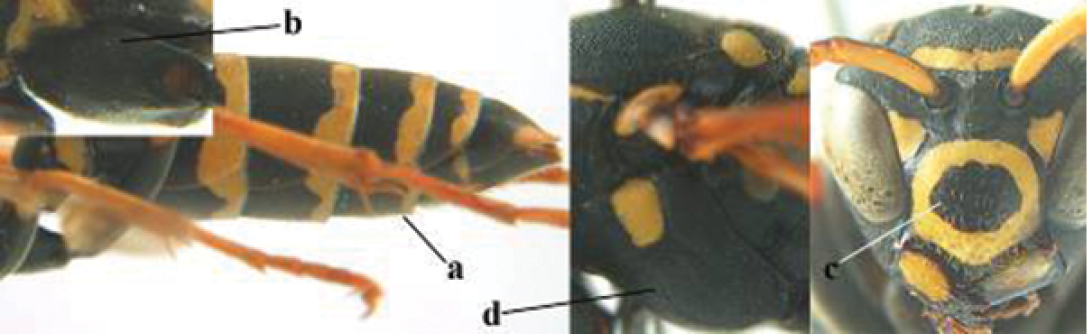	
–	Apical yellow band of sternite IV usually complete and wide (aa); hind coxa with yellow patch dorsally (bb); central dark patch on clypeus usually less developed and situated below middle of clypeus (ccc) or absent (cc); usually with an abrupt change in sculpture between mesepisternum and epicnemium (dd; = epicnemial ridge distinct), but sometimes rather gradual	**20**
	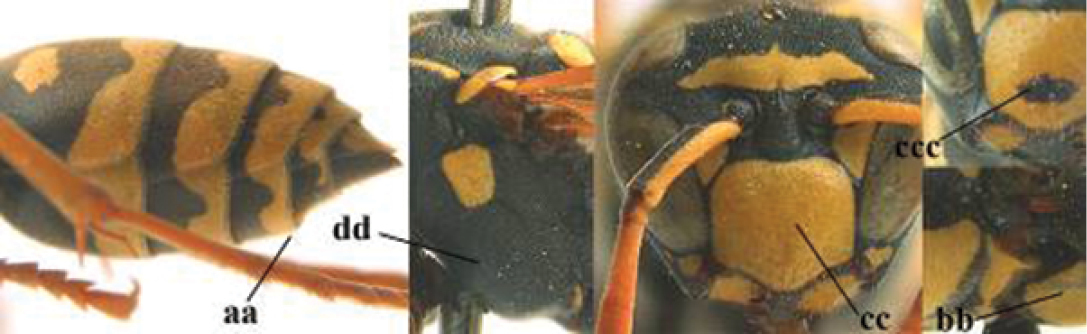	
20	Transverse yellow band of pronotum medio-laterally wider than dorso-laterally (a); central dark patch on clypeus usually developed, but small, rounded or forming a transverse band (b); mesoscutum usually with pair of medium-sized to large yellow spots (c); [some specimens have an all yellow clypeal disk and all black mesoscutum; specimens from NW Africa often have sternite IV apically yellow, which is more or less darkened in northern specimens]; NW Africa, SW Europe, N Italy, southern Switzerland, Croatia, Corfu	***P. gallicus* (Linnaeus, 1767)**
	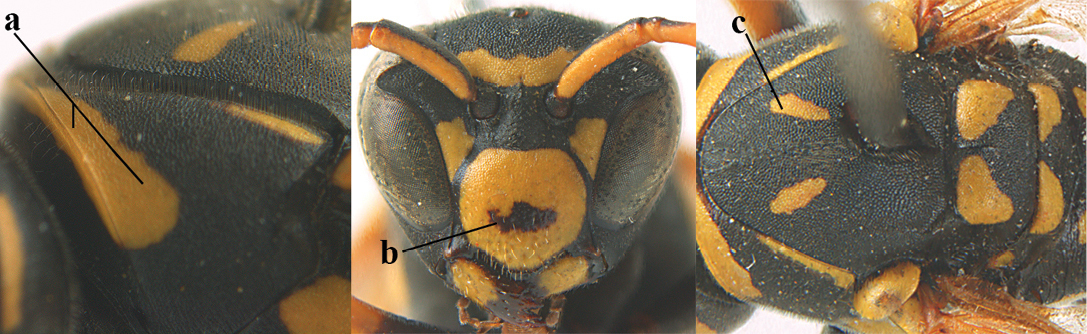	
–	Transverse yellow band of pronotum dorso-laterally wider than medio-laterally (aa); clypeus entirely yellow (bb) or with minute black spot, but spot sometimes medium-sized (bbb); mesoscutum usually black (cc) or with pair of small yellow spots (but sometimes large in Asian specimens; ccc); Croatia and SE Europe, Turkey, Cyprus to Central Asia and Egypt	***P. mongolicus* du Buysson, 1911, stat. rev.**
	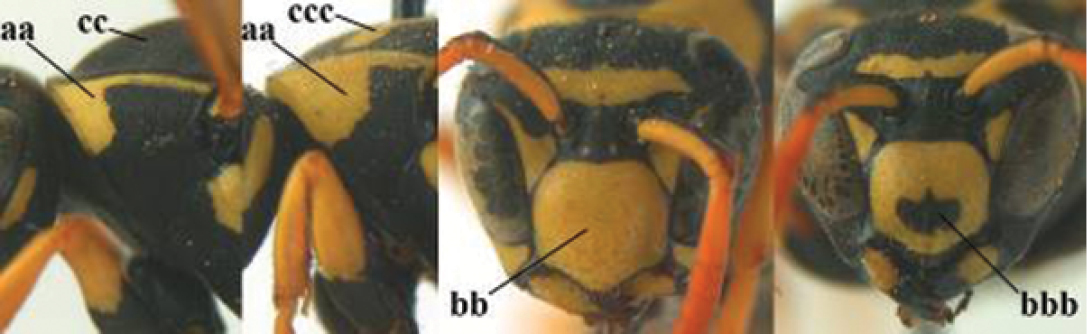	
**Males**
21	Mandible very stout and with a distinct depression on its outer face (a); clypeus depressed medio-apically (b), its margin tapered to a small point (c); malar space comparatively long and wide (d); socio-parasitic species	**22**
	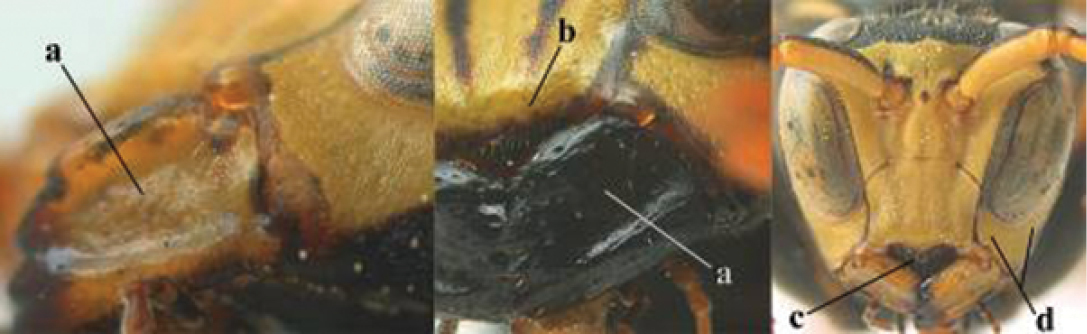	
–	Mandible comparatively slender and its outer face flat or slightly convex (aa); clypeus flat medio-ventrally (bb), its margin evenly convex or broadly triangular (cc); malar space medium-sized (dd) or narrow; social species	**24**
	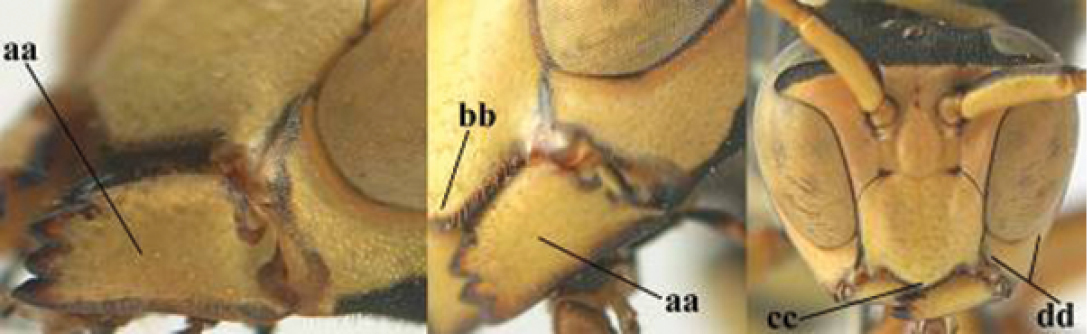	
22	Basal half of mandible sinuate in dorsal view (a); mandibular depression strongly concave (b); [mandible mainly yellow or brown (d); fore and middle coxae and mesopleuron ventrally usually with yellow pattern (c); dorsal ridge of mandible at most 0.25 times mandibular width and prominent]; SE and southern C Europe to Central Asia	***P. semenowi* Morawitz, 1889**
	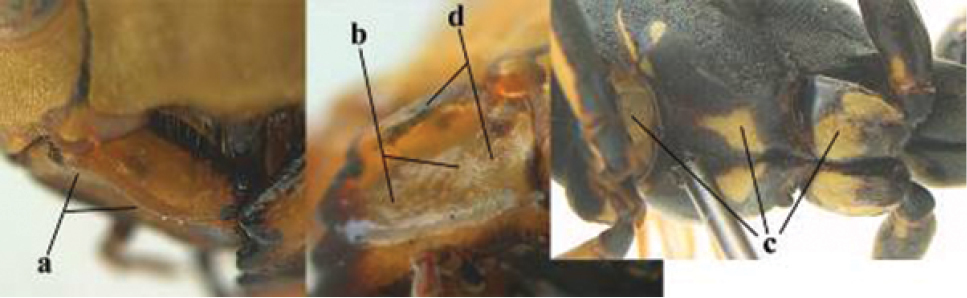	
–	Basal half of mandible straight in dorsal view or nearly so (aa); mandibular depression shallowly concave (bb); [fore and middle coxae and mesopleuron ventrally usually black (cc), colour of mandible variable (d, dd)]	**23**
	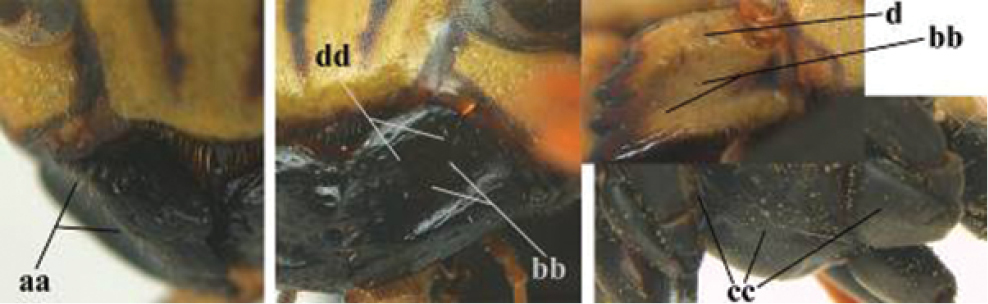	
23	Mandible mainly yellow, except for its more or less darkened margins (a); mandibular depression shorter and occupying less than half of outer face of mandible (b), dorsal ridge at least 0.33 times as wide as mandible (c); clypeus entirely yellow medially (d); SW Europe, southern C Europe, NW Africa	***P. austroccidentalis* van Achterberg & Neumeyer, sp. n.**
	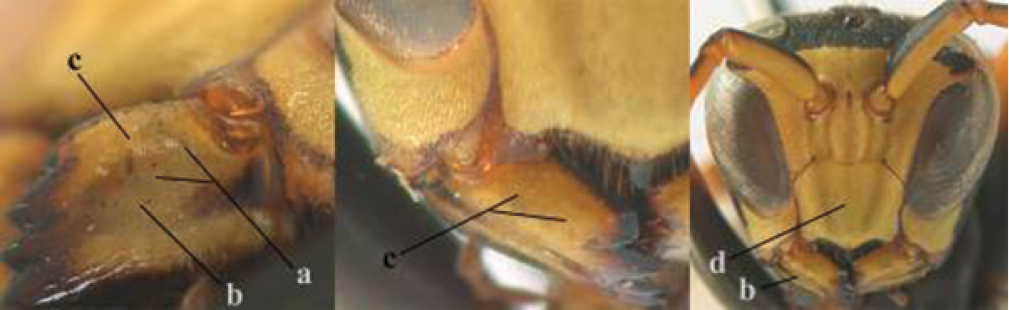	
–	Mandible black (aa), rarely with small yellow spot; mandibular depression wide and occupying most of outer face of mandible (bb), dorsal ridge 0.25–0.30 times mandibular width (cc); clypeus medially with dark brown pattern (dd), but sometimes largely reduced or absent; S Europe and southern C Europe to W Asia. [If from Morocco consider *P. maroccanus* (male unknown)]	***P. atrimandibularis* Zimmermann, 1930**
	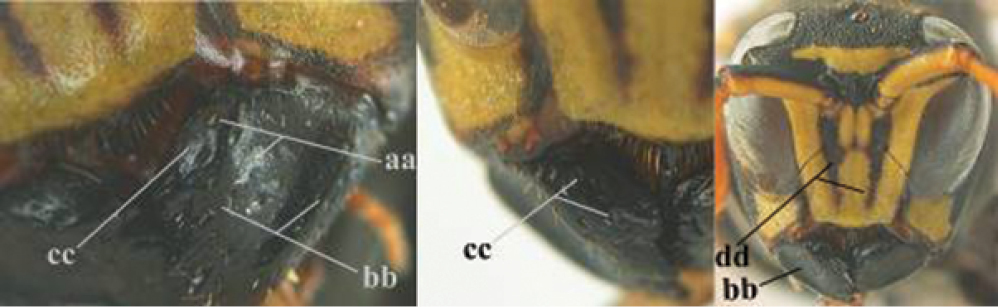	
24	Temple bulged behind eye in dorsal view, slightly convex (a); head trapezoid in anterior view (b); apical margin of clypeus triangular (c); width of clypeus 1.0–1.1 times its length (d, ddd); latero-ventral margin of clypeus narrowly black or dark brown and convex in lateral view (e), but flattened in *P. biglumis*	**25**
	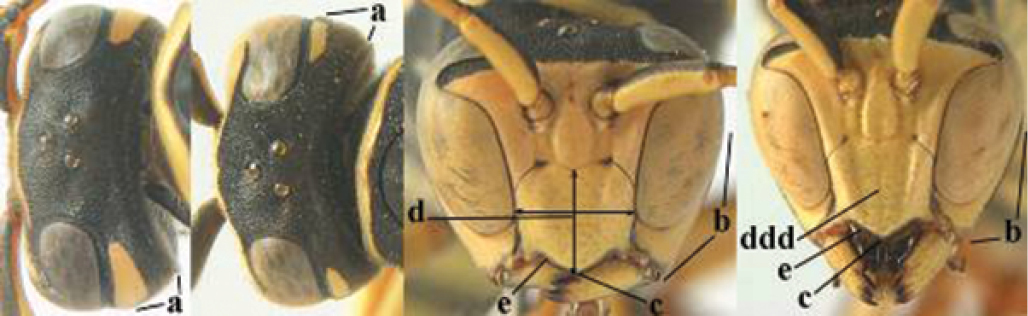	
–	Temple narrowed behind eye in dorsal view, more or less straight (aa); head nearly triangular in anterior view (bb), but less so in *P. bischoffi*; apical margin of clypeus rounded (cc) or subtruncate; width of clypeus 0.9–1.1 times its length (dd); latero-ventral margin of clypeus yellow and flattened (ee)	**28**
	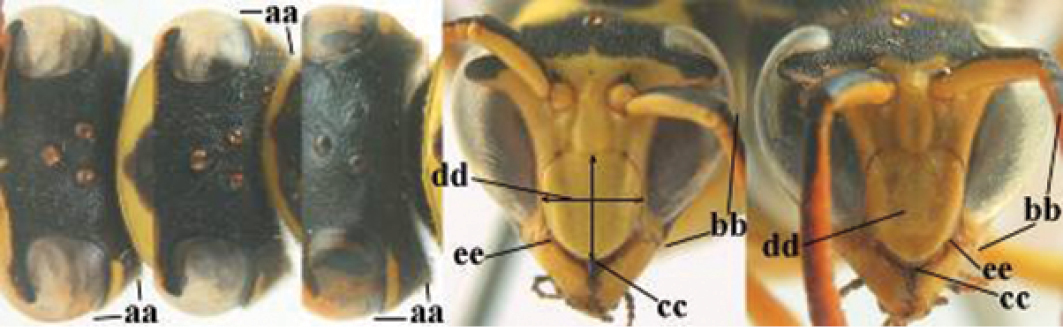	
25	Dorsal length of apical antennal segment 2.2–2.9 times as long as its maximal width (a), 1.3–1.5 times as long as fifth antennal segment (b); clypeus distinctly depressed medially (c) and with distinct lateral ridges (d); medio-longitudinal depression of face more or less impressed and U-shaped (e); antenna coloured according to fig. f; short carina between antennal sockets sharp dorsally and usually pale yellow (g); clypeus with short bristles and medio-ventrally flat; width of clypeus 1.0–1.1 times its length; [head sometimes distinctly narrowed ventrally]; Europe and Palaearctic Asia	***P. nimpha* (Christ, 1791)**
	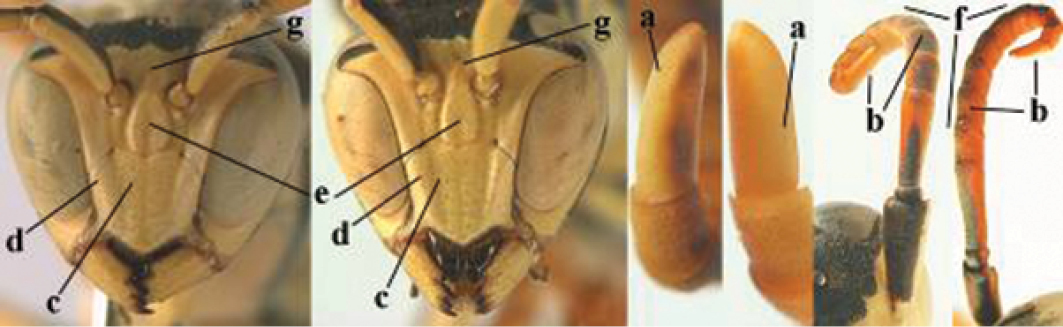	
–	Dorsal length of apical antennal segment 1.5–2.1 times as long as its maximal width (aa), 1.0–1.2 times as long as length of fifth antennal segment (bb); clypeus not or slightly depressed medially (cc) and lateral ridges absent dorsally or slightly developed (dd); face flat medially, without longitudinal depression (ee); antenna coloured as in ff or fff; short carina between antennal sockets obtuse dorsally and more or less infuscate (gg); width of clypeus 1.1–1.2 times its length	**26**
	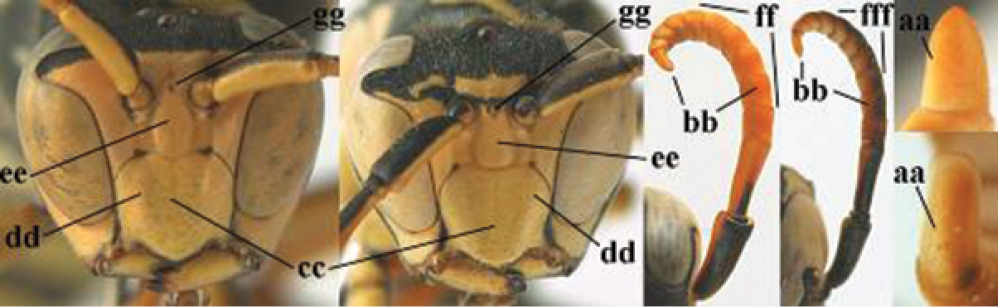	
26	Mesosternum entirely black (a); black baso-dorsal stripe on third antennal segment 0.25 times length of segment (b); lateral ridges of clypeus and ridge between antennal socket and clypeus more distinct (c); [largely yellow coloured species, specimens from eastern Turkey however may have reduced whitish yellow markings; W and C Asia, Cyprus, Egypt, Crete.	***P. bucharensis* Erichson, 1849**
	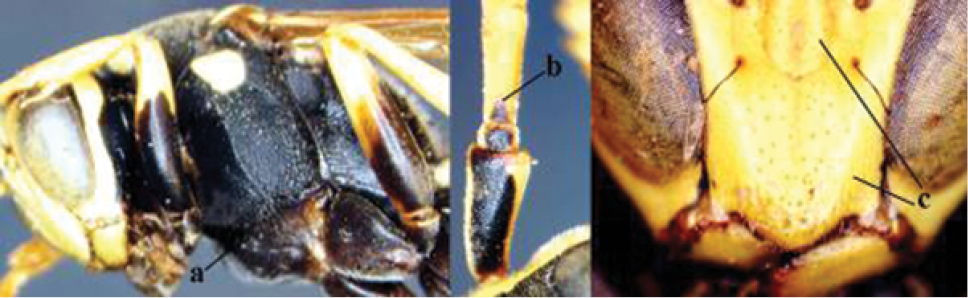	
–	Mesosternum at least with some yellow spots posteriorly (aa), and often entirely yellow, but in some males of *P. dominula* from southern Greece entirely black; black baso-dorsal stripe on third antennal segment at least 0.5 times length of segment (bb); clypeus laterally nearly flat and ridge between antennal socket and clypeus indistinct (cc)	**27**
	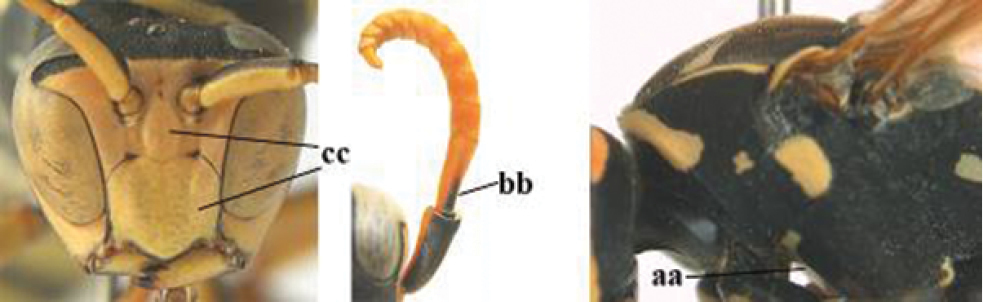	
27	Most of clypeus with long bristles and distinct punctures (a); apical half of antenna uniformly orange-yellow dorsally and ventrally (b); apical antennal segment comparatively stout and dorsally about 1.5 times as long as wide basally (c); setae of pronotum medio-dorsally and of mesoscutum about half as long as width of posterior ocellus (d); width of clypeus 1.1 times its length (e) and medio-ventrally more flattened (f); [fourth and fifth antennal segments strongly oblique in lateral view]; Europe (for specimens from Crete see also *P. bucharensis*), NW Africa, W and C Turkey, Azerbaijan, probably more eastern in temperate Asia	***P. dominula* (Christ, 1791)**
	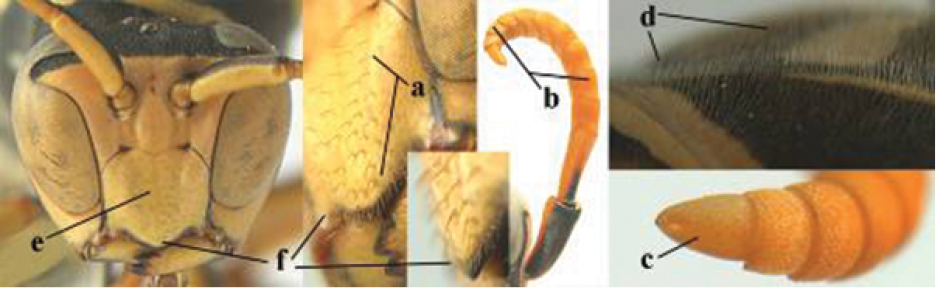	
–	Dorsal 0.7 of clypeus without bristles and punctures (aa); apical half of antenna usually darker dorsally than ventrally (bb); apical antennal segment more slender, dorsally about twice as long as wide basally (cc); setae of pronotum medio-dorsally and of mesoscutum at least 0.8 times as long as width of posterior ocellus (dd) or longer; width of clypeus about equal to its length (ee) and medio-ventrally less flattened (ff); [fourth and fifth antennal segments moderately oblique in lateral view]; Europe, temperate Asia	***P. biglumis* (Linnaeus, 1758)**
	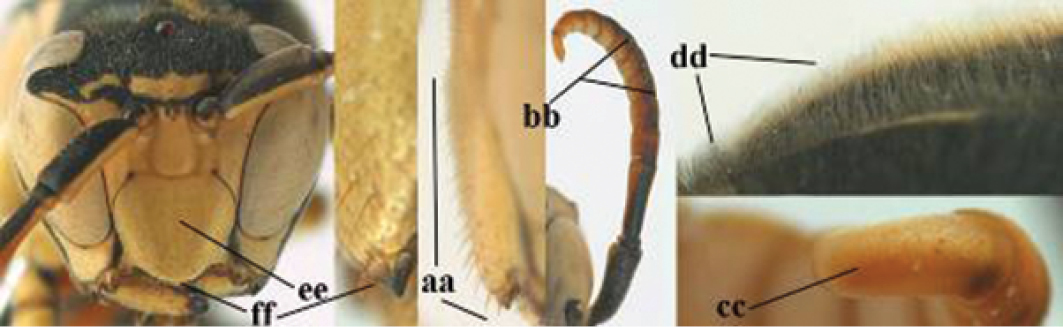	
28	Ventral half of clypeus distinctly depressed (a) and with distinct lateral ridges (b); dorsal length of apical antennal segment about 3.0 times its width (c); frons with a distinct longitudinal depression medially (d); sternites III-VII usually with transverse yellow band basally (e; visible with sternites sufficiently extruded) and last sternite often partly yellow (f); apical half of antenna usually distinctly darkened dorsally (g); clypeus more or less truncate medio-ventrally (h); S Europe, W Asia	***P. associus* Kohl, 1898**
	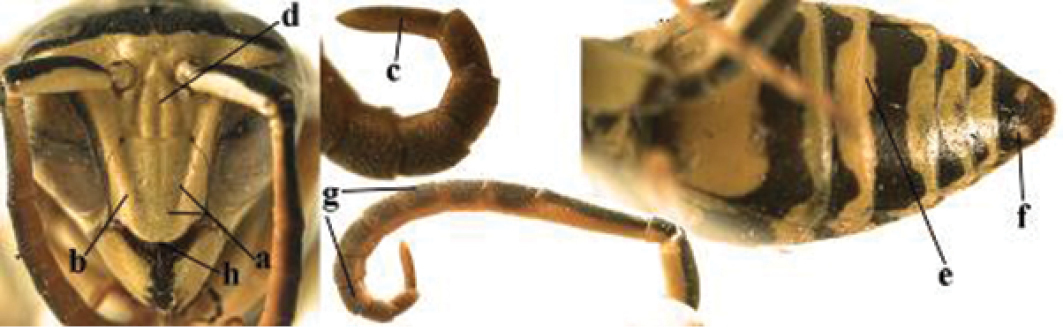	
–	Ventral half of clypeus at most slightly depressed (aa) but without lateral ridges (bb); dorsal length of apical antennal segment about twice its width (cc), but 2.2–2.7 times in *P. mongolicus* from Rhodes and Corfu; frons flat medially (dd), but sometimes with a shallow longitudinal depression (ddd); sternites III-VII black basally (ee) and last sternite entirely black or nearly so (ff); apical half of antenna evenly orange or yellow dorsally (gg), but darkened in *Polistes albellus* (ggg); clypeus usually more or less curved medio-ventrally (hh)	**29**
	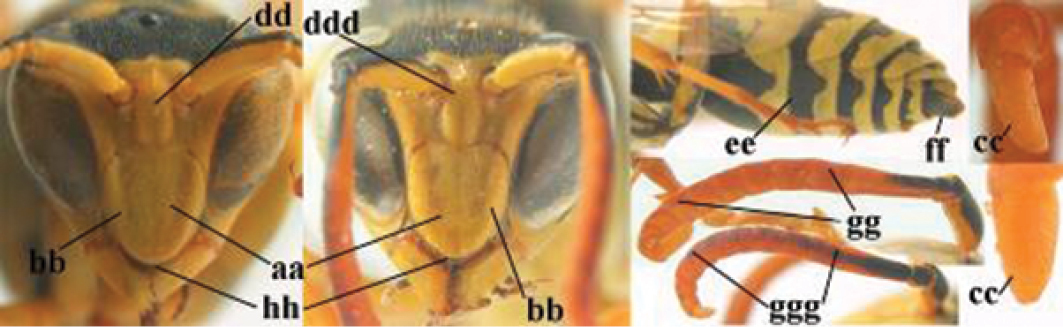	
29	Malar space long, 0.8–1.0 times as long as width of third antennal segment measured basally (a, b); malar space often entirely yellow (c) or narrowly black posteriorly; [basal half of second sternite with yellow pattern; mesopleuron obliquely rugulose (d)]	**30**
	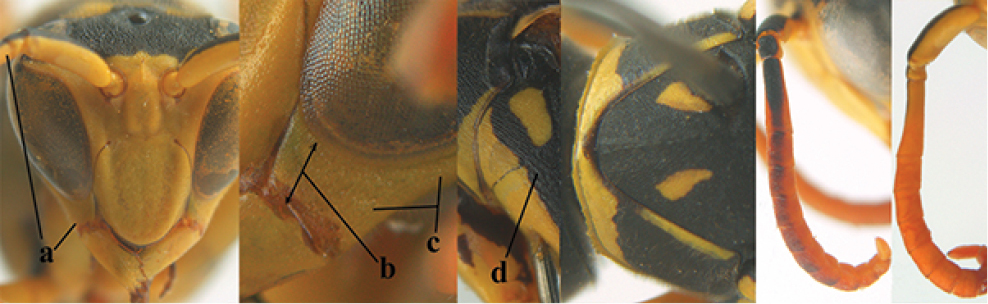	
–	Malar space short, 0.4–0.6 times basal width of third antennal segment (aa, bb); malar space black posteriorly with black area often widened (cc)	**31**
	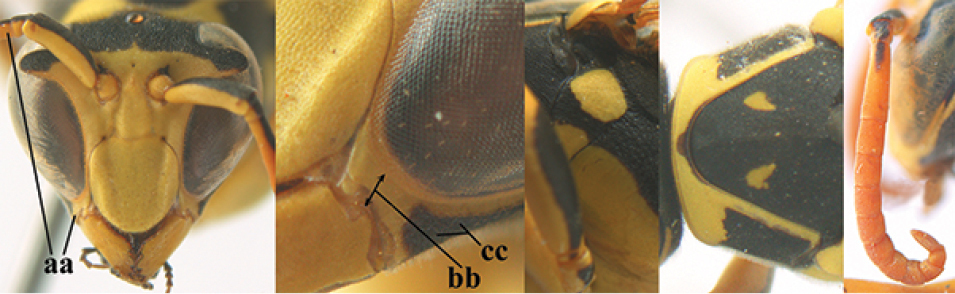	
30	Antenna dorsally orange-yellow (a); pronotal transverse yellow band more or less widened (b); epicnemial ridge delicate but well-defined (c), sometimes reduced in Peloponnesian populations; mesoscutum almost always with pair of medium-sized to large yellow spots (d), sometimes minute or absent; scutellar spots large (e); inner side of hind tibia often partly darkened (f); medial area of face narrower; N Italy to SE Europe, Turkey and Central Asia	***P. foederatus* Kohl, 1898**
	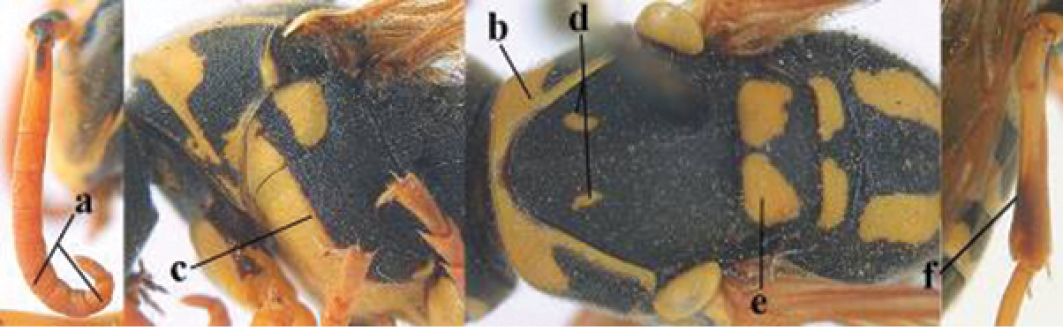	
–	Antenna dorsally dark brown (aa); pronotal transverse yellow band narrow (bb); epicnemial ridge indistinct (cc); mesoscutum with yellow spots minute or absent (dd); scutellar spots small (ee); inner side of hind tibia rather pale (ff); medial area of face wider; C Europe, temperate Asia	***P. albellus* Giordani Soika, 1976**
	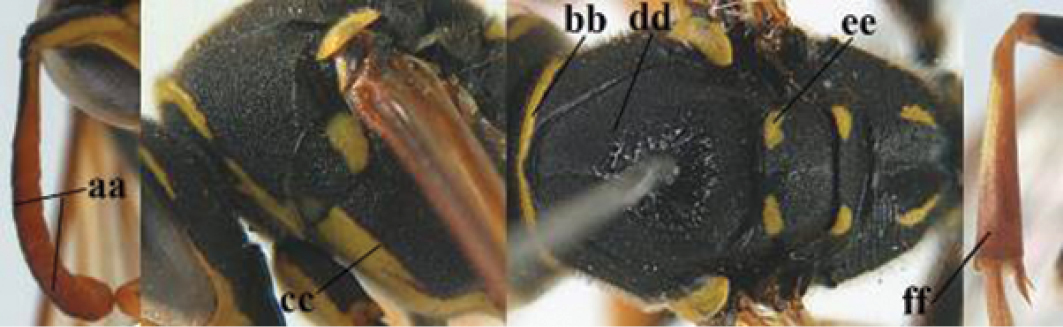	
31	Specimens from W to C Asia, Cyprus, Egypt	**32**
–	Specimens from Europe and NW Africa	**33**
32	Middle and hind coxae largely black dorsally (a); apical yellow band of sternite VI medially interrupted (b); Turkey	***P. bischoffi* Weyrauch, 1937**
	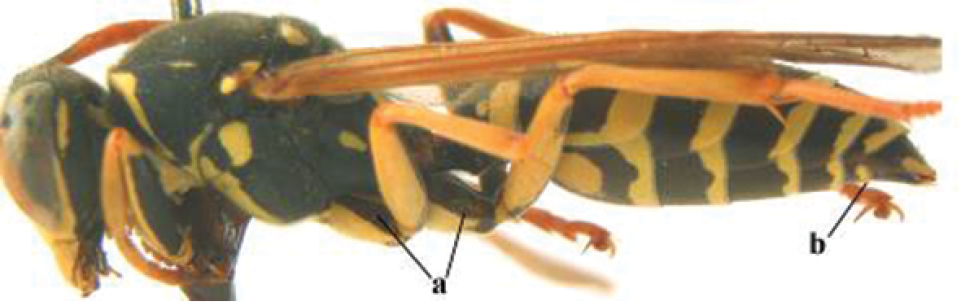	
–	Middle and hind coxae largely yellow dorsally (aa); apical yellow band of sternite VI medially continuous (bb); Cyprus, Turkey to Central Asia and Egypt	***P. mongolicus* du Buysson, 1911**
	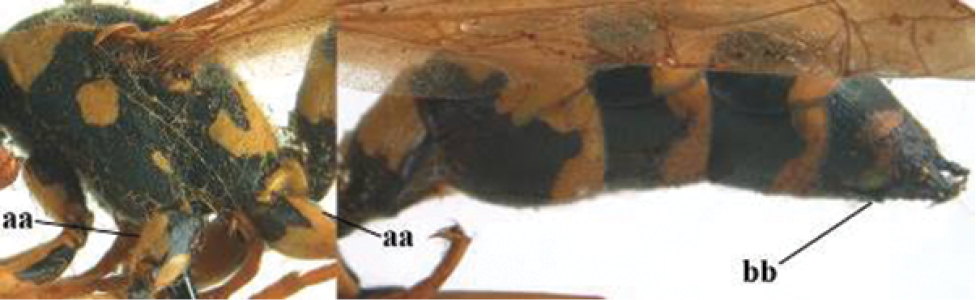	
33	Mesosternum entirely black or with a pair of elongate yellow spots (a); basal half of second metasomal sternite entirely black (b); oblique yellow stripes of pronotum often long or medium-sized (c); frontal ridge narrower (d); apical antennal segment slightly slenderer and usually parallel-sided basally (e); SE Europe	***P. mongolicus* du Buysson, 1911**
	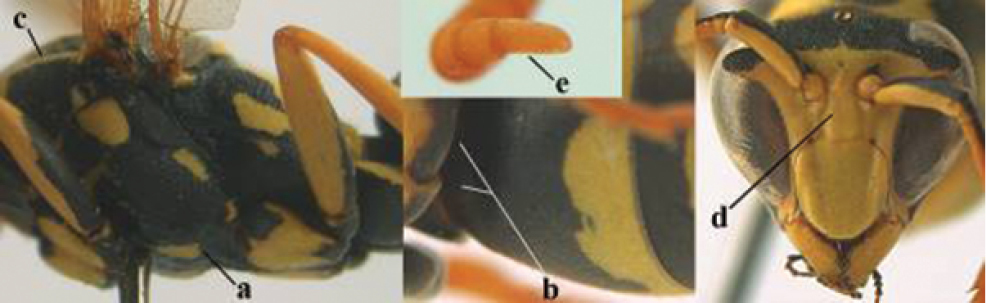	
–	Mesosternum predominantly or entirely yellow (aa); basal half of second sternite with yellow pattern, varying from a pair of very small sublateral yellow spots to mainly yellow (bb), **if** sometimes entirely black (*P. gallicus*) then pronotal transverse yellow band moderately widened; oblique yellow stripes of pronotum variable, sometimes absent (cc); frontal ridge wider (dd); apical antennal segment slightly less elongate and more or less widened basally (ee), but comparatively slender in *P. bischoffi* (eee)	**34**
	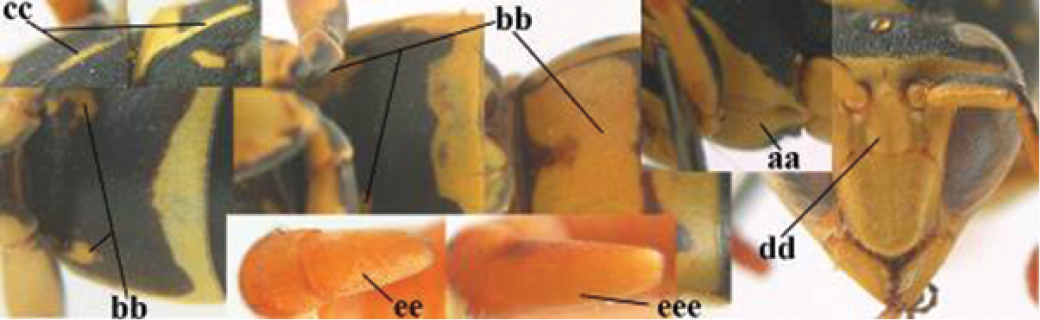	
34	Yellow transverse pronotal band narrow laterally (a), **if** widened dorso-laterally then narrowed medio-laterally (aaa); apical half of antenna slightly infuscate (b), but if partly darkened dorsally (bb), cf. *P. albellus*; mesoscutum without or with minute paired yellow spots (c); scutellum usually with comparatively small paired yellow spots (d); setae of pronotum and mesoscutum 0.5–0.7 times diameter of posterior ocellus and curved (e); southern C and S Europe, Turkey	***P. bischoffi* Weyrauch, 1937**
	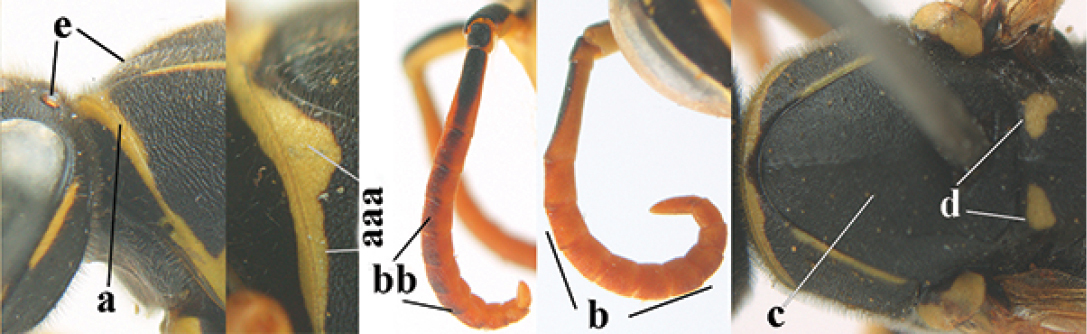	
–	Yellow transverse pronotal band moderately to strongly widened laterally, of even width medio-laterally (aa); apical half of antenna uniformly orange to yellow dorsally and ventrally (bb), mesoscutum nearly always with minute to large paired yellow spots (cc); scutellum with large yellow spots (dd); setae of pronotum and mesoscutum usually shorter, about 0.5 times diameter of posterior ocellus and mainly straight (ee); NW Africa, SW Europe, N Italy, S Switzerland, Croatia, Corfu	***P. gallicus* (Linnaeus, 1767)**
	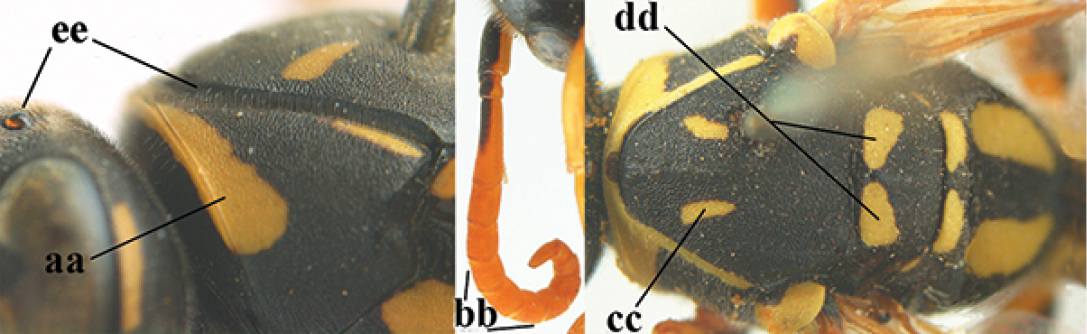	

### Species treatments

#### Genus *Polistes* Latreille

##### Subgenus Polistes Latreille

*Polistes* Latreille, 1802, Hist. Nat. Crust. Insect. 3: 363. – Type species: *Vespa gallica* Linnaeus, 1767, designated by Latreille, 1810, Consid. Gén. Crust. Arachn. Insect.: 438.

*Polystes* [sic!]; Palisot de Beauvois, 1818, Insect. Recueill. Afrique Amérique: pl. 8; Buysson, 1892, Ann. Soc. Entomol. France 61: 59; H. von Ihering, 1896, Zool. Anz. 19: 452. Invalid emendation.

*Eupolistes* Dalla Torre, 1904, Genera Insect. 19: 68. – Name for “Premiere division” of *Polistes* Latreille in de Saussure, 1853, Et. Fam. Vesp. 2: 45 (61 species). Type species: *Vespa gallica* Linnaeus, 1767, designated by Richards, 1973, Rev. Bras. Entomol. 17 (13): 86.

*Pseudopolistes* Weyrauch, 1937, Zool. Jahrb. (Abt. Syst. Ökol. Geogr. Tiere) 70: 266, 274, genus (three species). – Unavailable; no type species designated.

*Sulcopolistes* Blüthgen, 1938 (1937), Konowia 16: 273. – Subgenus of *Polistes* Latreille. Type species: *Polistes semenowi* Morawitz, 1889, by original designation.

*Polistula* Weyrauch, 1938, Arbeit. Physiol. Angewand. Entomol. 5 (3): 273, genus (5 species). – Unavailable; no type species designated.

*Polistula* Weyrauch, 1939, Arch. Naturgesch. (N. F.) 8(2): 148. – Genus. Type species: *Polistes kohli* Dalla Torre, 1904 [= *Polistes biglumis* Linnaeus, 1758], by original designation.

*Pseudopolistes* Weyrauch, 1939, Arch. Naturgesch. (N. F) 8(2): 195. Validation by type selection of *Pseudopolistes* Weyrauch, 1937. Type species: *Polistes sulcifer* Zimmermann, 1930, by original designation.

*Leptopolistes* Blüthgen, 1943, Arch. Naturgesch. (N. F) 12(1): 99, 121. – Subgenus of *Polistes* Latreille. Type species: *Polistes associus* Kohl, 1898, by original designation.

###### Polistes albellus

Taxon classificationAnimaliaHymenopteraVespidae

Giordani Soika

Fig. 1

Polistes bischoffi Weyrauch, 1937, Zoologische Jahrbücher (Jena), Abteilung für Systematik, Ökologie und Geographie der Tiere 70: 274, in part. – Mixed type series, see Neumeyer et al. (2014). Most citations of P. bischoffi from Central Europe refer to P. albellus.
Polistes foederatus albellus Giordani Soika, 1976, Acta zoologica Academiae Scientiarum Hungaricae 22(3–4): 272 – Holotype female (HNHM, currently on loan, not examined), type locality: Bulgan aimag: Namnan ul mountains, 23 km NW of Somon Chutag, Mongolia (1 paratype female in MSNV, examined by RN).
Polistes helveticus Neumeyer, 2014, ZooKeys, 400: 101-108. – Holotype female (NMBE, examined by RN), type locality: Schwerzenbach (Switzerland).
Polistes albellus – Neumeyer et al. 2015, Boletín de la Sociedad Entomológica Aragonesa (S.E.A.) 57: 206–211. – Species status.

####### Remarks.

*Polistes albellus* was confused with *P. bischoffi* before Neumeyer et al. (2014) clarified the status of the latter by designating a neotype. He re-described the former under the new name *P. helveticus*. Later, Neumeyer et al. (2015) synonymised *P. helveticus* with *P. albellus*, a species described from Mongolia. Genetic results confirmed the conspecificity of the European populations with the Central Asian specimens (for detailed descriptions and discussion see Neumeyer et al. 2014, 2015).

The species typically occurs on humid meadows or along lake shores or in fens with a large reed zone, but it also colonizes dry habitats. In contrast to *P. biglumis*, *P. albellus* does not occur at higher altitudes of the Alps. Both species may sometimes occur sympatrically in lowland habitats.

####### Diagnosis.

The female is characterized by reduced yellow markings and dorsally dark antennal segments. It can be confused mainly with *P. biglumis*. Distinction of the females of the two species is problematic and they are most easily separated by the lack of an epicnemial ridge in combination with an even transition in sculpture from the coarser sculptured mesopleuron to the finer sculptured epicnemium in *P. albellus*. *Polistes biglumis* is characterized by having an epicnemial ridge, with a sudden change from the rather coarse sculpture of mesopleuron to the finer sculpture of the epicnemium. In addition, the mesoscutal setae are shorter in *P. albellus* than in *P. biglumis*.

The male is unique within the *gallicus*-group by the combination of narrow temples (genae) in dorsal view and the antenna, with nearly black or dark brown dorsal surface. Females from Central Asia are darker than European individuals (clypeus may be all black with small basal pale spot), and yellow markings are largely replaced by white or ivory.

**Figures 1–12.**
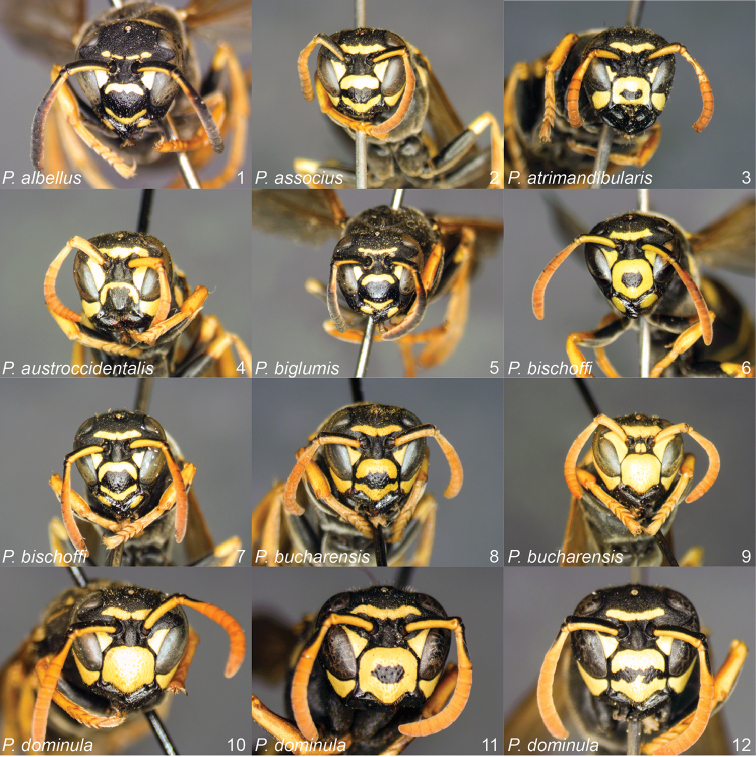
*Polistes* heads in frontal view. **1** *P. albellus* **2** *P. associus* **3** *P. atrimandibularis* **4** *P. austroccidentalis* sp. n. **5** *P. biglumis*, **6** *P. bischoffi* (Switzerland) **7** *P. bischoffi* (France) **8** *P. bucharensis* (Crete) **9** *P. bucharensis* (Turkey) **10** *P. dominula* (Germany) **11** *P. dominula* (Hungary) **12** *P. dominula* (Hungary).

**Figures 13–24.**
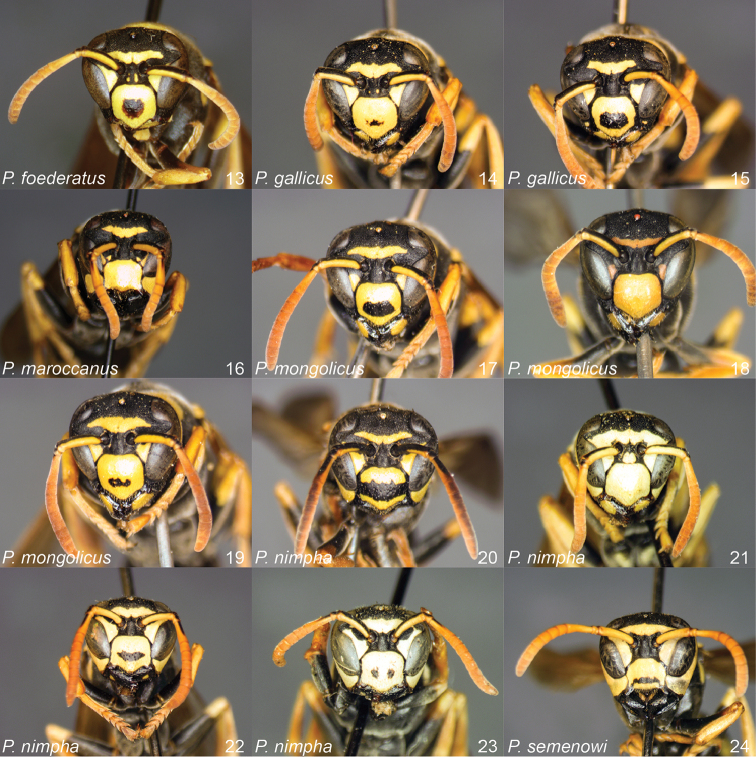
*Polistes* heads in frontal view. **13** *P. foederatus* (Greece) **14** *P. gallicus* (Spain) **15** *P. gallicus* (Italy) **16** *P. maroccanus* sp. n. **17** *P. mongolicus* (Greece) **18** *P. mongolicus* (Croatia) **19** *P. mongolicus* (Croatia) **20** *P. nimpha* (Germany), **21** *P. nimpha* (Turkey), **22** *P. nimpha* (Turkey) **23** *P. nimpha* (Turkey), **24** *P. semenowi*.

####### Distribution.

*Polistes albellus* has a wide distribution, ranging from eastern France to the Pacific coast of the Russian Far East, although latitudinally remaining roughly between the 44th (France, Lardiers: 44°03'N) and the 53rd (Russia, Donskoye: 52°03'N) northern latitude (Neumeyer et al. 2015). In Germany, the northernmost records are from central Hessen (Tischendorf et al. 2015) and Ebergötzen (Niedersachsen, 51°34'N 10°06'E, Neumeyer et al. 2015). The species usually occurs at elevations below 1000 m a.s.l.

####### Specimens examined.

Europe: France (eastern part), Belgium, Switzerland, Germany, Austria, Czech Republic, Russia (Orenburg Oblast). Asia: Kazakhstan, Mongolia, Russia (Primorsky Krai), China.

####### Genetic results.

Specimens from Switzerland, Germany, and regions as far apart as Kazakhstan exhibited little intraspecific variation (0.16%, Table 1).

###### Polistes associus

Taxon classificationAnimaliaHymenopteraVespidae

Kohl

Fig. 2

Polistes associa Kohl, 1898, Ann. Naturh. Hofmus., Wien 13: 89 + Taf. III. – Syntypes males (NHMW, male from Poros examined by RN & CvA), type localities Poros (Greece) and “Helenendorf” [Göygöl], Azerbaijan. Male from Poros designated as lectotype by Blüthgen on label, but not in Blüthgen (1943: 121). The male is herewith designated, **new designation**.

####### Diagnosis.

The recognition of *P. associus* females may be problematic because of their similarity to *P. nimpha*, in particular specimens from SW Asia. Females can be separated by colour differences only (see key to species), although in western Asia *P. nimpha* often exhibits high levels of colour variation.

The male is unique by the combination of narrow temples (genae) in dorsal view and a markedly depressed clypeus with distinct lateral ridges. The dorsal length of the apical antennal segment is about 3.0 times its maximum width, and longer than in similar species.

####### Distribution.

Southern Europe and Turkey, northwards to Switzerland, southwards to Israel, eastwards to Azerbaijan. Guiglia (1972) also mentions India (Jammu and Kashmir) and China, but these records may refer to the similar species *P. chinensis* (Fabricius, 1793).

####### Specimens examined.

Europe: Spain, France, Italy, Switzerland, Croatia, Bulgaria, Macedonia, Montenegro, Greece. Asia: Israel.

####### Genetic results.

We regard *P. associus* as a member of the *P. dominula* group instead of the *P. gallicus* group due to the results of genetic data (see discussion below for details). Specimens from Croatia and northern Italy exhibited no intraspecific variation (Table 1).

###### Polistes atrimandibularis

Taxon classificationAnimaliaHymenopteraVespidae

Zimmermann

Fig. 3

Polistes atrimandibularis Zimmermann, 1930, Mitt. Zool. Mus. Berlin 15: 611. – Holotype male (MFNB, examined by CSE), type locality: Toblach, “[Süd]Tirol 19.8.1908” (Italy).
Polistes atrimandibularis albidus Blüthgen, 1957, Revue de la Faculté des Sciences de l’Université d’Istanbul. Série B 22 (3): 164. Holotype male (MFNB, not examined), type locality: Ulu Dagh [Uludağ], Turkey. Probably a synonym of P. atrimandibularis.
Sulcopolistes atrimandibularis – Guiglia (1972), new combination.

####### Diagnosis.

The social parasitic species can be recognized by the shape of the mandibular impression and by the colour pattern of the clypeus. Females of the species group show differences in the depth of the medial impression of the mandible and the size and shape of the upper ridge. The weakest medial impression occurs in *P. maroccanus*, whose upper ridge is only weakly developed or even lacking in the paratype. It is followed by *P. atrimandibularis* with a shallow impression, and flat, but visible upper ridges. The medial impression is deep in *P. austroccidentalis*, with large but not modified upper ridges, whereas in *P. semenowi* the impression is very deep with large and narrow upper ridges. Additionally, the lower ridge is modified with a triangular margin in dorsal view. The size of the black clypeal spot is variable but as a general rule it is largest in *P. austroccidentalis* (only upper third of clypeus yellow), medium-sized in *P. semenowi* (lower third black only), small and isolated in *P. atrimandibularis*, and even smaller (isolated) or completely lacking in *P. maroccanus*.

The shape of the mandibular impression in males generally follows that of females, but is in general less developed. In addition, the male of *P. atrimandibularis* has the mandible and parts of clypeus black (almost entirely so in some specimens, except lateral margins), whereas mandible and clypeus are yellow in the remaining species. The male of *P. maroccanus* is unknown.

####### Distribution.

Southern C and S Europe, northwards to S Germany, Turkey, Iran, Armenia (Guiglia, 1972). Records from NW Africa probably refer to *P. maroccanus* sp. n. A male from the MFNB from “Ägypten [Egypt], Ehrenberg [leg.]” is probably mislabelled because Egypt is far outside the known range of the species.

####### Specimens examined.

Europe: Bulgaria (Rhodope Mts), France, Greece, Italy (Alps, Abruzzi), Spain, Switzerland.

####### Biology.

*P. atrimandibularis* is a social parasite of *P. biglumis*. In Greece, it was collected together with *P. biglumis* (2 males 20.ix.1989, Mt. Olympos, eastern slope, 2200-2500 m a.s.l., T. Osten leg., in coll. CSE).

####### Genetic data.

Not enough specimens were sequenced to detect any genetic variation.

###### Polistes austroccidentalis

Taxon classificationAnimaliaHymenopteraVespidae

van Achterberg & Neumeyer sp. n.

http://zoobank.org/25A2E89A-26D5-414A-A5EB-7701EB831BDA

Figs 4 , 25–32 , 33–36 , 37–45

Polistes semenowi auctt., nec Morawitz, 1889.

####### Type specimens.

Holotype, ♀ (RMNH), “**España**, Burgos, Las Macharras, 23.iv.1984, R. Leys”. Paratypes: 1♀ (RN0706), **Algeria**, Algiers, [unknown collector] (ETHZ); 1♂, Sid bel Abbes [unknown collector] (MFNB); 1♂, Blidah, Medeah, vii/viii.1884, Quedenfeld (MFNB); 2♀, **Andorra**, St. Julia, 21.vi.1981 & 1.v.1985, P.J.L. Roche (RMNH); 1♂ (GBIFCH00281850), **France**, Alpes-Maritimes, Lucéram, Peïra-Cava, 1400 m, viii.1950, Matthey (MZL); 1♀ (GBIFCH00281951), Bouches-du-Rhône, Eygalières, 13.viii.1964, D. Petitpierre (MZL); 1♂ (GBIFCH00281950), Saint-Rémy-de-Provence, 24.viii.1964, D. Petitpierre (MZL); 2♀, Camargue, Salin de Badon, 26.v.1952, H. Engel (RMNH); 1♀, id., but Astoin, 1.v.1981, R. Leys (RMNH); 1♀ (GBIFCH00281955), Haute-Savoie, Pied du Salève, 3. ix.1933, J. de Beaumont (MZL); 1♀, Hérault, Notre Dame de Londres, 8 km N of Les Matelles, 7.vii.1990, L. Blommers (RMNH); 1♀, Landes, Linxe, 21-30.vi.1968, R.T. Simon Thomas (RMNH); 1♀, Lot, Le Montat, 14-19.v.1986, A.D.J. Meeuse, on *Euphorbia* (RMNH); 1♀, Lozère, St. Enimie, Le Buisson, near Quézac, along Tarn, 21-28.vi.1986, P. Thomas (RMNH); 1♂, Vaucluse, Carpentras, 30-31.vii.1951, P.M.F. Verhoeff (RMNH); 1♀, Vaucluse, Bedoin, 1.vi.1993, H. & J.E. Wiering (RMNH); 1♀, id., but Rustrel, 300 m, 12.ix.1999 (RMNH); 1♀, Pyrénées-Orientales, Banyuls-sur-Mer, 5-200 m, 12.vii.1965, R.T. Simon Thomas; 1♀, Drôme, Espenel, 25.vii.1979, V. Lefeber (RMNH); 1♀, Montpellier/Lac du Salagou, 18.v.1986 & 1♀, 1♂, Narbonne plage, 11.ix.1987 & 1♂, Alpes Maritimes, Tende, 1000 m NN, 12.vii.2009 & 1♂, Pyrenees, Pic du Canigou, 1700 m, 13.ix.1987 & 1♂, Sisteron/Serres, 6.ix.1997, C. Schmid-Egger (CSE); 16♂ 25♀, Digne, 1957, Schewen, (ZSM), 1♂, 3♀, Champs de Bes, 1957, Schewen (ZSM) 1957; 1♀, Camargue, 13.vi.1852, Forster (ZSM); 1♂ (RN0690), **Italy**, Tuscany, Passo della Cisa (“Colle la Cisa”), 19.viii.1949, A. Nadig (ETHZ); 1♀, Aosta, 27.v.27, Bischoff (MFNB); 1♀ (RN0704), **Morocco**, Fès-Meknès, Ifrane, 22-24.vii.1932, A. Nadig (ETHZ); 1♂ (RN0722), Taza, 24.vii.1931, A. Nadig (ETHZ); 2♀ (RN0697, RN0699), 2♂ (RN0698, RN0723), Marrakesh-Safi, Asni, 10-14.vii.1932, A. Nadig (ETHZ); 1♂ (RN0705), Marrakesh, 6-18.vii.1932, A. Nadig (ETHZ); 2♀ (RN0700, RN0701), 2♂ (RN0702, RN0703), Tanger-Tétouan-Al Hoceïma, Tanger, 4.vii.1932, A. Nadig (ETHZ); 1♂, Atlas moyenne, Azrou, 2.vii.1926, Landshut (MFNB); 1♂, Aoulouz, 12.vi.2014 & 1♂, Haut Atlas, Aguelmouss, 13.vi.2014, 2070 m & 1♂, Haut Atlas, Tiz n’Tichka, 28.ix.2016, C. Schmid-Egger (CSE); 1♀, **Portugal**, C. Alentejo, Montfort, Vaiamonte, iii.2012, A. v. Harten (RMNH); 1♀, Porto, (MFNB); 1♀ (RN0721), **Spain**, Asturias, ≤ 1898, [unknown collector] (ETHZ); 1♀ (RN0695), Community of Madrid, El Escorial, R. García Mercet (ETHZ); 1♀ (RN0696), Rivas, J.M. Dusmet (ETHZ); 1♀, Alicante, Benidorm, 28.iv.1993, V. Lefeber (RMNH); 3♂, Granada, Sierra Nevada, near Albergue Universitario, 2500–2600 m, 16.vii.1953, C.A.W. Jeekel (RMNH); 1♂, id., but km 24 road Granada-Pic. de Veleta, 1700 m, 19.vii.1953, (RMNH); 1♂, Granada, Orgiva, 22.vii.1969, H. Overbeek (RMNH); 2♂, Santander, Enterrias, 30.viii.-5.ix.1969, M.C. & G. Kruseman (RMNH); 1♀, 1♂, Teruel, Albarracin, 27.ix.1963, (RMNH); 1♂, id., but 17.ix.1963 (RMNH); 1♂, Lerida, Artesa de Segre, 41°54'N, 1°3'E, 30.vii.1969, C. v. Heijningen (RMNH); 1♂, Málaga, Rincón de la Victoria, 6.vi.1967, M.J. & J.P. Duffels (RMNH); 1♀ Tiermas, viii.1926, Dusmet; 1♂,Villamartín, 30.vii.1950, Verhoeff; 1♂, Valle de Ordesa, vii 1923, Seitz; 2♀, Peña de Francia, Prov. Salamanca, Krichelsdorf; 1♀, Sta. Maria, Andalusia, vi.1993, Hering; 2♀, Aranjuez, 27.v.1920, Dusmet (MFNB); 1 ♂, San Fernando, 6.vi.1998, Kroupa (CSE); 1♀, Estepona [near Málaga], 1.-11.iv.1985, H. Wolf (C. Saure); 1 ♂ (RN0713), **Switzerland**, Canton Valais, Erschmatt, Bawald, 13.vii.2003, A. Breitenstein (ETHZ); 1♀ (GBIFCH00281987), Fully, Les Follatères, 11.vi.1932 & 1♀ (GBIFCH 00281986), 19.vii.1935, P. Bovey (MZL); 1♂ (RN0710), 12.viii.1941, A. Nadig (ETHZ); 2♂ (RN0711, RN0712), 23.vii.-2.viii.1942, A. Nadig (ETHZ); 1♀ (GBIFCH00281988), 13.v.1947, J. Aubert (MZL); 1♂ (RN0296), Gampel, Jeizibärg, 46°19’14.19"N, 07°43’53.53"E, 1090 m, rocky steppe, 10.viii.2013, R. Neumeyer (RN); 1♂ (GBIFCH00110807), Gampel, Jeizinen, 46°19’21.06"N, 07°43’50.81"E, 1200 m & 2♂ (GBIFCH00110810, GBIFCH00110811), 46°19’33.47"N, 07°44’02.57"E, 1330 m & 1♂ (GBIFCH00110809), 46°19’31.91"N, 07°43’36.85"E, 1430 m & 1♂ (GBIFCH00110808), 46°19’35.15"N, 07°43’36.86"E, 1480 m, 3.viii.2015, R. Wenger (RW); 1♀ (GBIFCH00281992), Martigny, viii.1932 & 1♀ (GBIFCH00281991), 29.viii.1933, R. Matthey (MZL); 2♀ (GBIFCH00281989, GBIFCH00281993), 29.iv.1934 & 1♀ (GBIFCH00281962), 28.iv.1935 & 1♀ (GBIFCH00281990), 14.vi.1936, J. de Beaumont (MZL); 1♂ (RN0707), Mörel, 19.viii.1916, [unknown collector] (ETHZ); 1♀ (GBIFCH00281985), Sierre, 26.v.1931, J. de Beaumont (MZL); 2♂ (RN0708, RN0709), 23.vii.-2.viii.1942, A. Nadig (ETHZ); 1♂ (RN0689), Stalden, ≤ 1893, [unknown collector] (ZMUZ); 1♂ (RN0714), ≤ 1893, [unknown collector] (ETHZ).

####### Remarks.

Konstantin Samartsev (ZISP) kindly provided photos of the female lectotype of *P. semenowi* Morawitz, 1889, from Copet-dag. Their examination showed unambiguously that the specimen is conspecific with *P. sulcifer* (Zimmermann, 1930). This requires the species *P. semenowi* of authors to be described as a new species: *P. austroccidentalis* van Achterberg & Neumeyer, sp. n.

####### Diagnosis.

Large and relatively bright species with robust mandible and wide, yellow malar space (Figs 29, 31, 35, 36), entirely yellow flagellum, black mesosternum and change in sculpture between mesepisternum and epicnemium rather abrupt (epicnemial ridge distinct) in both sexes. Outer face of mandible with distinct depression between a wide dorsal ridge and a much narrower ventral one; dorsal ridge of mandible convex and distinctly elevated above middle of mandible. Female clypeus mainly black, basally yellow (Fig. 29); mesoscutum with pair of yellow spots (Figs 26, 33); hind coxa black; yellow area along inner eye margin usually connected with yellow bar above antennal sockets (Fig. 29); hypopygium with yellow tip; basal half of mandible gradually curved in dorsal view and convex; clypeus abruptly depressed ventrally. Male with mandible mainly yellow, except for its more or less darkened margins (Figs 44, 45); mandibular depression rather short and occupying less than half of outer face of mandible, and dorsal ridge wide; clypeus medially entirely yellow, face and frons yellow (Fig. 42); temple (or gena) in dorsal view convex (Fig. 43). See also comments of *P. semenowi* for recognising the species.

**Figures 25–32.**
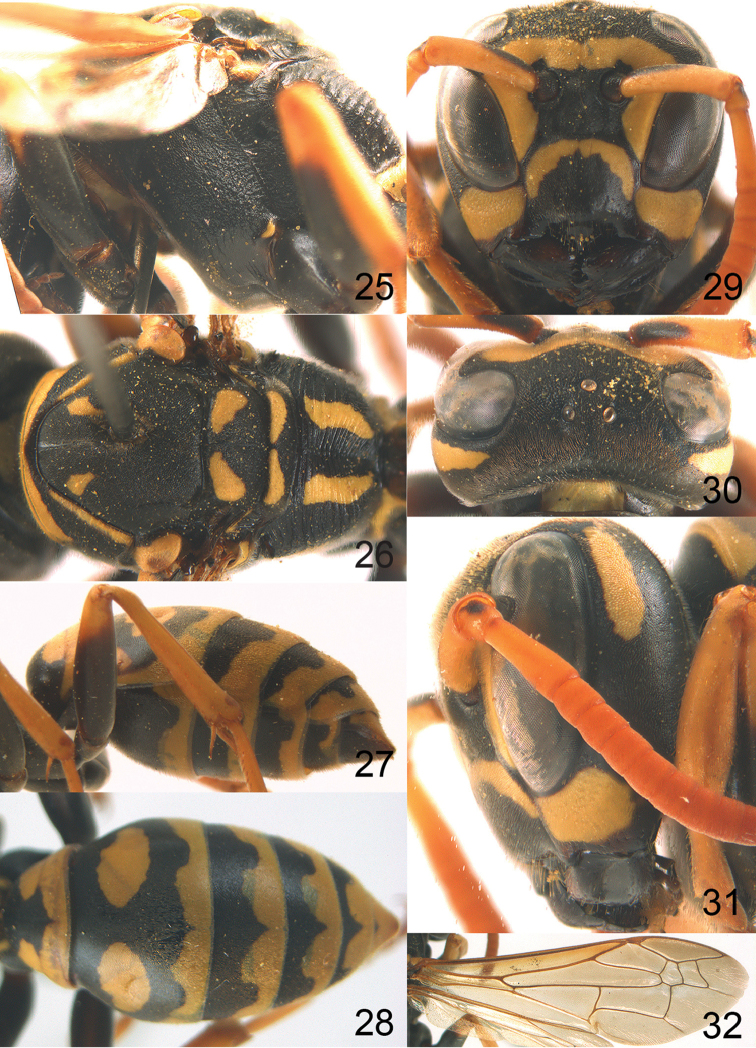
*Polistes austroccidentalis* sp. n. Holotype female. **25** Mesosoma in lateral view, **26** mesosoma in dorsal view **27** metasoma in lateral view **28** metasoma in dorsal view **29** head in frontal view **30** head in dorsal view **31** head in lateral view **32** fore wing.

**Figures 33–36.**
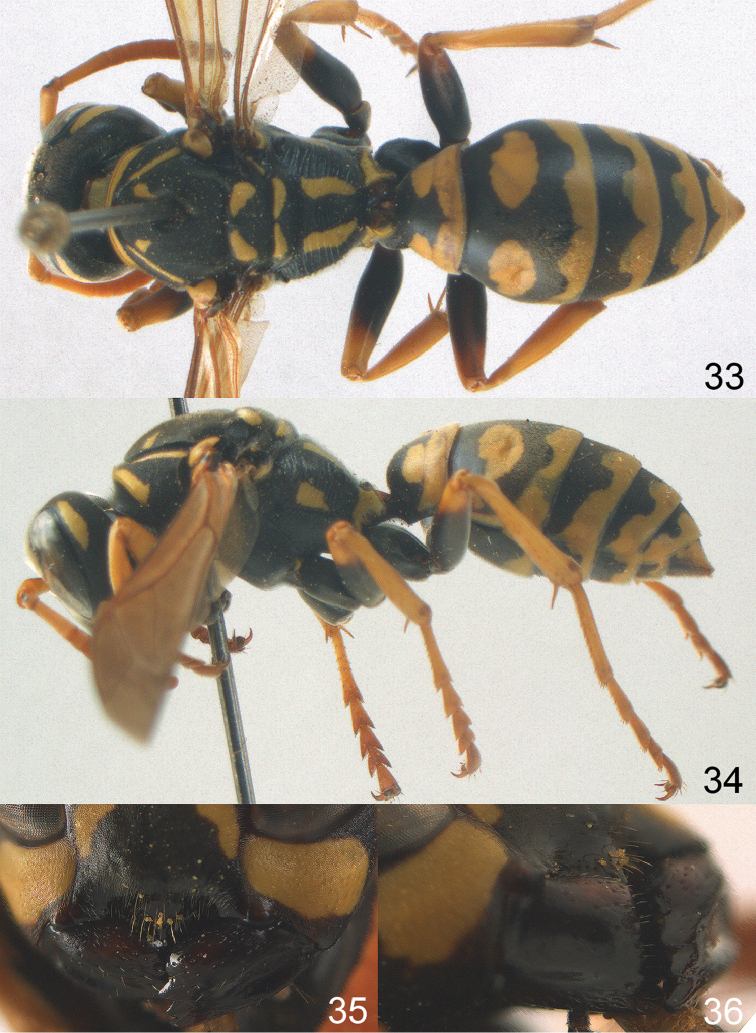
*Polistes austroccidentalis* sp. n. Holotype female. **33** Habitus, dorsal view **34** habitus lateral view **35** lower part of head in frontal view **36** gena and mandibles.

**Figures 37–45.**
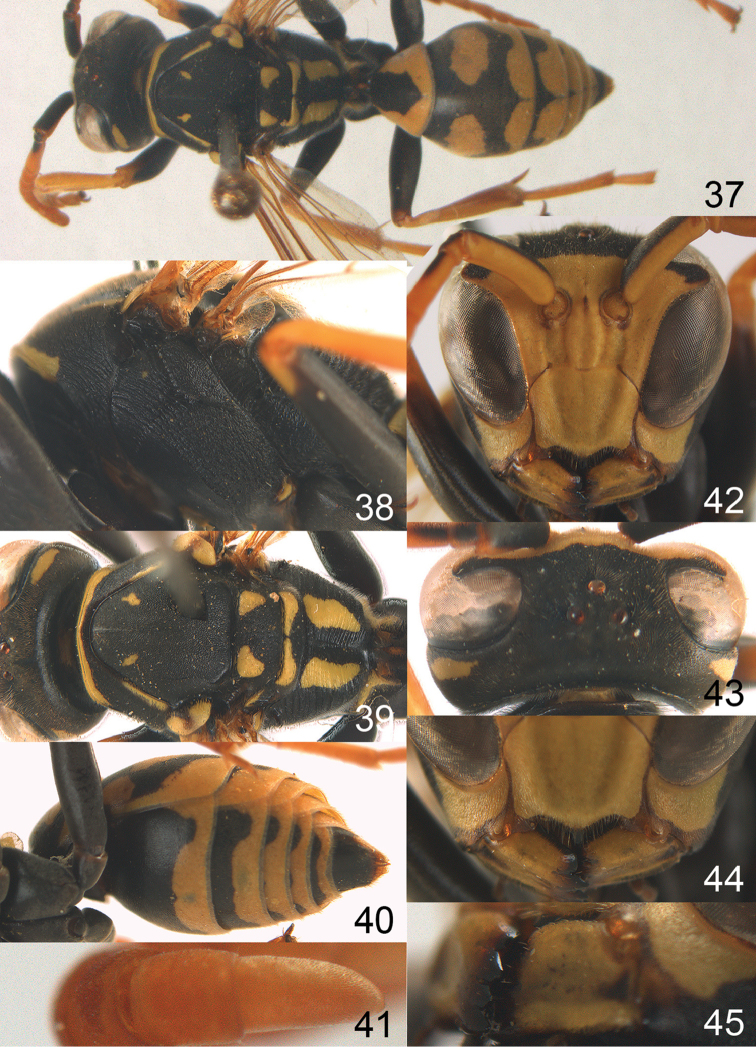
*Polistes austroccidentalis* sp. n. Paratype male. **37** habitus, dorsal view, **38** mesosoma in lateral view **39** mesosoma in dorsal view **40** metasoma in ventrolateral view **41** apex of antenna **42** head in frontal view **43** head in dorsal view **44** lower part of head in frontal view **45** gena and mandibles.

####### Description.

FEMALE. Holotype, body length 15.8 mm; fore wing length 11.6 mm.

For colour pattern, see figures.

*Head*. Mandible very stout and 1.5 times as long as wide (Figs 35, 36) and with a large depression on its outer face; basal half of mandible gradually curved in dorsal view and convex; dorsal ridge of mandible wide, smoothly convex without sharp edges and distinctly elevated above depression; ventral lobe of clypeus acute and step-like lowered; fine pubescence of clypeus conspicuous and comparatively long ventrally (Figs 29, 31); malar space 1.8 times POL; ocelli in equilateral triangle (Fig. 30).

*Mesosoma*. Posterior half of pronotum obliquely rugose and with short pubescence, only medio-anteriorly with longer setae; epicnemial ridge distinct and abruptly separating rugulose mesepisternum from smoothly sculptured epicnemium; propodeum coarsely transversely striate (Figs 25, 26). Fore wing distinctly infuscate anteriorly (Fig. 32), pterostigma and veins brown.

*Variation*. Body length 13.0–17.1 mm; fore wing length 10.1–12.7 mm. Mandible either black (Europe) or partially yellowish (NW Africa), if with a yellowish or brownish area then that area always smaller than yellow area on malar space. Vertex often with pair of tiny yellow spots behind lateral ocelli. Third antennal segment often with tiny basal dark brown spot, about 5% of specimens have the yellow posterior stripes of the pronotum connected to the transverse yellow band. Tergite II with paired anterolateral yellow spots absent (NW Africa) or present (Europe). Hypopygium usually with yellow tip, but sometimes apical half or nearly entirely yellow, or entirely blackish and only dark brown apically.

MALE. Body length 11.6–17.1 mm; fore wing length 9.6–11.7 mm. For colour pattern, see figures. Similar to female, differs as follows:

*Head*. Mandible except for darkened margins yellow (Figs 44, 45). Clypeus yellow, and laterally distinctly convex, sub-antennal depressions continued onto clypeus (Fig. 44). Malar space yellow and 1.4 times as long as POL. Temples in dorsal view convex (Fig. 43). Face and anterior half of frons yellow, at most a tiny black spot on interantennal prominence and frequently also a narrow vertical black dash originating from upper margin of each torulus. Vertex often with pair of small yellow dots behind lateral ocelli, remainder of head black. Antenna brownish yellow, but scapus and pedicellus dorsally as black as a spot on third antennal segment (Figs 37, 42). Apical antennal segment 2.2 times as long as wide (Fig. 41).

*Mesosoma*. Mesoscutum with paired medium-sized yellow spots, sometimes reduced to tiny dots or absent (Figs 37, 39). Dorsal yellow spot of mesopleuron large but rarely minute, often only apex of femora yellowish.

*Metasoma*. Pair of spots of tergite I either connected to terminal band or well separated (Fig. 37). Sternites II-VI with continuous yellow terminal bands (Fig. 40), sometimes briefly interrupted on sternite VI. Hypopygium black with brown margin (Fig. 40).

####### Distribution.

Specimens from Algeria, Andorra, France, Italy, Morocco, Portugal, Spain, and Switzerland have been examined, indicating that the species is confined to NW Africa and SW Europe with an extension to Central Europe, and is replaced by *Polistes semenowi* Morawitz further east. In Switzerland, *P. austroccidentalis* occurs only in the SW part (Valais and one record in the Jura Mountains), whereas *P. semenowi* occupies mainly the SE part (Ticino and southern Grison valleys, except for two records from the canton of Valais).

####### Biology.

According to Cervo (2006), *P. austroccidentalis* is an obligate social parasite, normally of *P. dominula*, but occasionally also of *P. nimpha*. Corresponding to its ubiquitous, euryoecious main host (*P. dominula*), *P. austroccidentalis* can be found in a wide variety of open and semi-open habitats, but up to now it apparently avoids the northern part of its host‘s range. The altitudinal records (n= 20) for *P. austroccidentalis* range from near sea level (Carpentras, France) to 2600 m in Spain (Sierra Nevada, Andalusia) and 2150 m in Morocco. The seasonal records (n = 67) range from March (Vaiamonte, Portugal) to 21 September in Switzerland (Ausserberg, VS) or 28. September in Morocco (males only), but most individuals were observed from May to August, at least in Switzerland (CSCF in litt.). There, the earliest record for males is 12 July (Erschmatt, VS), the latest for females 29 August (Martigny, VS).

####### Genetic data.

Specimens from south-central Europe and Morocco only showed a small genetic distance.

####### Etymology.

The name is a combination of the Latin adjectives “australis” (southern) and “occidentalis” (western), because of its southwestern distribution in Europe.

###### Polistes biglumis

Taxon classificationAnimaliaHymenopteraVespidae

(Linnaeus)

Fig. 5

Vespa biglumis Linnaeus, 1758, Systema naturae 1 (Editio decima): 573. – Holotype female (LSL, examined by RN), designated by Day (1979), type locality: Europe.
Vespa rupestris Linnaeus, 1758, Systema naturae 1 (Editio decima): 573. – Holotype male (LSL, examined by RN), designated by Day (1979), type locality: Sweden.
Vespa bimaculata Geoffroy in Fourcroy, 1785, Entomologia parisiensis, sive catalogus insectorum quae in agro parisiensi reperiuntur, vol. 2: 433. – Holotype female (type lost; see Blüthgen 1961: 54), type locality: near Paris, France.
Polistes geoffroyi Lepeletier & Serville, 1825, In: Latreille M (Ed) Encyclopédie Méthodique, Histoire Naturelle. Insectes. Vol. 10: 173. – Syntypes males, females (MNHN, 1 female and 1 male examined by RN), type locality: France.
Polistes dubia Kohl, 1898, Annalen des kaiserlich-königlichen Naturhistorischen Hofmuseums, Wien 13: 90 + Taf. III. – Lectotype male (NHMW, examined by RN & CvA), designated by Blüthgen (1943: 128), type locality: Brühl, Austria.
Polistes kohli Dalla Torre, 1904, Vespidae. Genera Insectorum 19: 70. – Replacement name for Polistes dubia Kohl, 1898, nec de Saussure, 1867.
Polistes bimaculatus var. arduinoi Guiglia, 1948, Mem. Soc. Entomol. Ital. 27, Fasc. Suppl.: 14 (key), 22. – 2 syntype females (MSNG, not examined), type locality: Ponte di Legno, Lombardia (Italy).
Polistes pamirensis Zirngiebl, 1955: Mitt. Münchner Entomol. Ges. 44/45: 381–383. – Syntypes 4 females (ZSM, examined by RN), type locality: “Umss-Tugai”, probably in the area of eastern Uzbekistan to southwestern Tadjikistan.
Polistes pamirensis var. soikai Zirngiebl, 1955, Mitt. Münchner Entomol. Ges. 44/45: 383. –Holotype female (ZSM, examined by RN), type locality: “Umss-Tugai”, probably in the area of eastern Uzbekistan to southwestern Tadjikistan.
Polistes pamirensis var. interruptus Zirngiebl, 1955, Mitt. Münchner Entomol. Ges. 44/45: 383. – Holotype female (ZSM, examined by RN), type locality: “Umss-Tugai”, probably in the area of eastern Uzbekistan to southwestern Tadjikistan.
Polistes bimaculatus var. pamirensis Zirngiebl, 1955: 385, var. status.
Polistes bimaculatus var. nigrinotum Zirngiebl, 1955, Mitt. Münchner Entomol. Ges. 44/45: 385. – Holotype female (ZSM, examined by RN), type locality: Althegnenberg, Germany.
Polistes biglumis alpium Blüthgen, 1957, Rev. Fac. Sci. Univ. Istanbul, Ser. B, 22 (3): 163. Holotype female (MFNB, examined by CSE). Type locality: Ulu Dagh [Uludağ], Turkey.

####### Remarks.

See Neumeyer et al. (2014) for detailed comments about taxonomy.

####### Diagnosis.

The female shares a dark brown to black upper side of antennal segments with *P. albellus* (see under *P. albellus* for recognition). The other species of the *P. gallicus* group have lighter antennal segments (except northern *P. foederatus*). The male has broad, convex temples in dorsal view and the clypeal disk without impressions or lateral ridges, similar to that of *P. dominula* and *P. bucharensis*.

####### Distribution.

Europe including Norway and Sweden south of 65° N to Turkey, Central Asia. Guiglia (1972) also mentioned N Africa, Iran and Mongolia, but these records require confirmation. *Polistes biglumis* occurs up to 2400 m in the European Alps. The presence in Greece is confirmed by a male from Mt. Olympus, eastern slope, 2200-2500 m a.s.l., 20.ix.1989, leg. T. Osten, coll. CSE), removing doubts expressed by Arens (2011).

####### Specimens examined.

Europe: Austria, Belgium, France, Germany, Greece, Italy, Netherlands, Sweden, Switzerland. Asia: Tajikistan, Turkey, Uzbekistan.

####### Genetic data.

*Polistes biglumis* consists of two closely placed subclusters which share the same BIN (Suppl. material 2: NJ tree, and Fig. 58). Specimens from the Aosta valley in NW Italy form a geographic subcluster that is separated from a second subcluster consisting of specimens from NW Italy and SE Germany.

**Figures 46–49.**
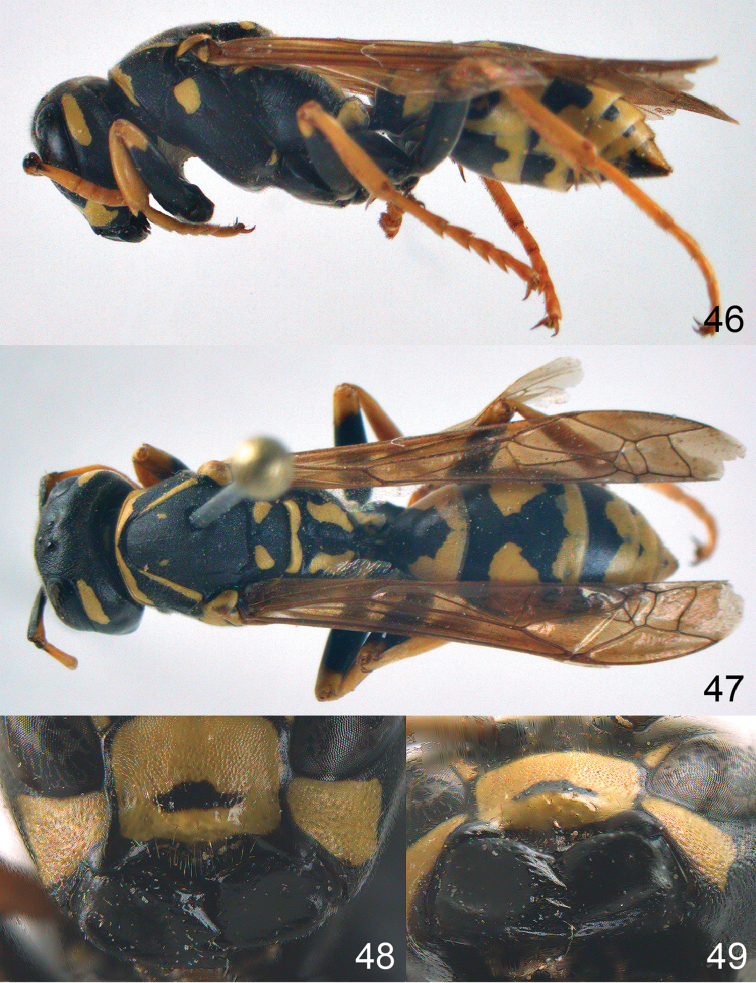
*Polistes maroccanus* sp. n. Holotype female. **46** habitus, lateral view **47** habitus, dorsal view **48** lower part of head in frontal view **49** head in ventral view.

**Figures 50–56.**
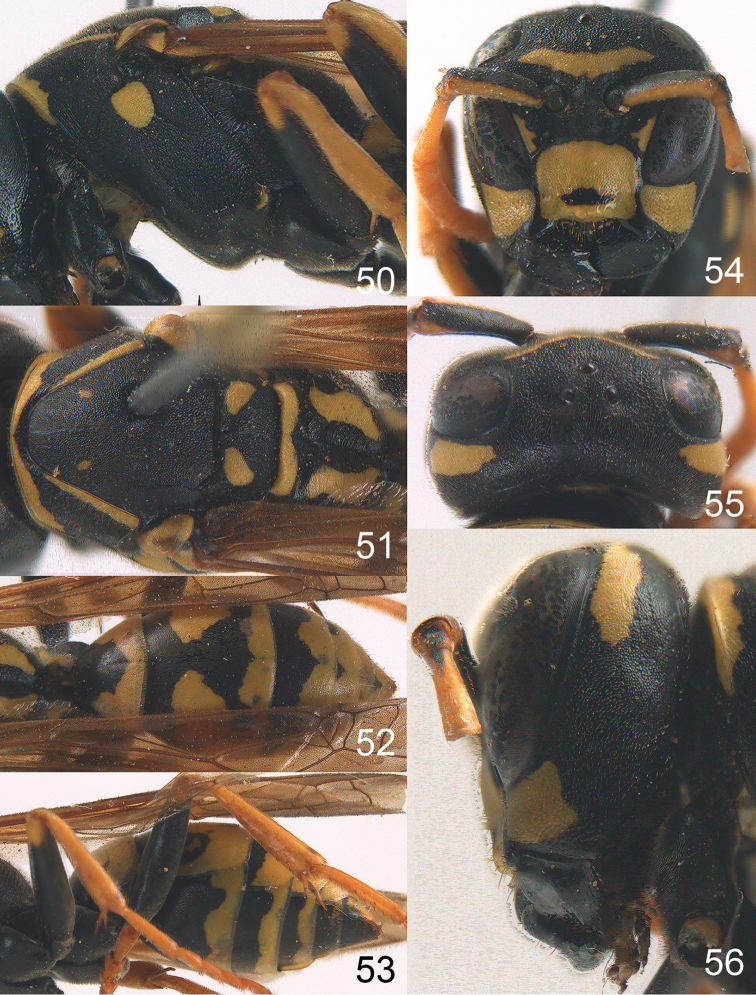
*Polistes maroccanus* sp. n. Holotype female. **50** mesosoma in lateral view, **51** mesosoma in dorsal view **52** metasoma in dorsal view **53** metasoma in ventrolateral view **54** head in frontal view **55** head in dorsal view **56** head in lateral view.

###### Polistes bischoffi

Taxon classificationAnimaliaHymenopteraVespidae

Weyrauch

Figs 6 , 7

Polistes bischoffi Weyrauch, 1937, Zoologische Jahrbücher (Jena), Abteilung für Systematik, Ökologie und Geographie der Tiere 70: 274. – Neotype female (NMBE, examined by RN, designated by Neumeyer et al. 2014), type locality: Galeria, Corsica (France).

####### Remarks.

For a detailed discussion about the mixed type series of the species see Neumeyer et al. (2014, and references therein). Weyrauch (1937) described *P. bischoffi* from Sardinia and later (Weyrauch 1939) he included a dark form of *P. bischoffi* (actually *P. albellus*) that he interpreted as geographic variations (followed by Schmid-Egger and Treiber 1989, and others). The true *P. bischoffi* has a southern European distribution, whereas *P. albellus* ranges from Central Europe to the eastern Palaearctic region.

####### Diagnosis.

This species belongs to the group of species with a short female malar space within the *P. gallicus* species group. The female can further be recognized by a medially interrupted yellow band on sternite IV, a large black spot or band on the clypeus, and by the dorsally black hind coxa. The sternal band IV is always continuous in *P. gallicus* and *P. mongolicus*, and the clypeus is entirely yellow in most *P. mongolicus* specimens or has a smaller, more excentric spot in most *P. gallicus* specimens. The epicnemial ridge is reduced or absent in *P. bischoffi* and *P. albellus* versus distinct or reduced in *P. gallicus* and *P. mongolicus*. The character is therefore of limited diagnostic value. The flagellum is in both sexes dorsally light orange to somewhat dark orange, whereas it is always light orange in related species, except northern *P. foederatus*. The male can be confused with *P. gallicus* (see the key to species for differences).

####### Distribution.

S Europe and Turkey, northwards to Austria (Neusiedl am See) and Switzerland (northwards to Zürich). See Neumeyer et al. (2015) for the detailed distribution.

####### Specimens examined.

Europe: Spain, Gibraltar, Switzerland, southern France and Corsica, Austria, Greece, Italy, Croatia. Asia: Eastern Turkey (Hakkari region).

####### Genetic results.

Specimens from Spain, S France and Corsica, Switzerland, and Croatia were barcoded. The species forms a separate cluster, with a small gap between SW (France, Spain) and southern central European populations.

###### Polistes bucharensis

Taxon classificationAnimaliaHymenopteraVespidae

Erichson stat. rev.

Figs 8 , 9

Polistes bucharensis Erichson, 1849, Mem. Acad. Sci. St. Petersburg (6)6: 307. – Holotype female (depository unknown), type locality: Uzbekistan “Bokhara“ (= Bukhara). **Sp. restit.**
Polistes gallica var. ornata Weyrauch, 1938, Arbeit. Physiol. Angewand. Entomol. Berlin-Dahlem 5: 278, nec Lepeletier, 1836. – Female lectotype (lost), designated by Weyrauch (1939: 154), type locality: Astrabad [Gorgan], Iran. **Syn. n.** (previously considered a synonym of P. dominula)
Polistes gallica var. pacifica Weyrauch, 1939, nec Fabricius, 1804, Arch. Naturgesch. (N. F.) 8: 155–156. – Holotype female (depository unknown), type locality: China. Synonymy likely but requiring confirmation through examination of types.
Polistes gallicus pseudopacificus Giordani Soika, 1970, Ann. Hist.-Nat. Mus. Natl. Hung., Zool. 62: 326. Replacement name for *pacificus* Weyrauch, 1939, nec Fabricius, 1804.
Polistes iranus Guiglia, 1976, Boll. Mus. Civ. Stor. Nat. Venezia 28 (1976): 99; description of male and female. – Holotype female (MSNV, not examined), type locality „Daria Namak“ [salt steppe near Dariun], Fars (Iran). **Syn. n.**
Polistes gallicus muchei Gusenleitner, 1976, Nachrichtenbl. Bayer. Entomol. 25(6): 118. – Holotype male (OLL, not examined), type locality: “Kislovods” [Kislovodsk], northern Caucasus (Russia). Paratypes from Kislovodsk and from Turkey/Artvin. Six male paratypes (MFNB, examined by CSE). **Syn. n.** (previously considered a synonym of P. dominula)

####### Remarks and genetic data.

*Polistes bucharensis* is recognized as a valid species distinct from *P. dominula* here. Its taxonomic status has been controversial. It was formerly treated as valid species (*P. iranus*) by Guigla (1976), as subspecies of *P. dominula* (e.g. Gusenleitner 1976), or as a synonym of *P. dominula* (Carpenter 1996). In our opinion *P. bucharensis* is clearly distinguishable from *P. dominula* by colour pattern, by shape of the male clypeus and by the genetic data.

Specimens from Mongolia and China (= *Polistes gallicus pseudopacificus*) probably belong to *P. bucharensis* and not to *P. dominula*, as indicated by Giordani Soika (1970).

The species was first described from Uzbekistan and later as Polistes gallica var. ornata and as *Polistes iranus* from Iran. It was not possible to examine types of these taxa, but their descriptions agree well with examined specimens. Also, we could examine a large series of specimens from the type locality of *P. bucharensis* from “Bukhara” in MFNB, which also agree with description of *P. bucharensis*. Therefore, the valid name for this taxon is *Polistes bucharensis* Erichson, 1849 sp. restit.; and *Polistes iranus* syn. n. is a new synonym of *P. bucharensis*.

Another problematic taxon in this group is *P. gallicus muchei*. It was described by Gusenleitner (1976) from northern Caucasus and eastern Turkey as a subspecies of *P. gallicus* (now *P. dominula*) based on reduced pale marks in the male and whitish yellow or ivory instead of yellow ground colour. Six male paratypes and a non-type male from eastern Turkey (Kars, identified by J. Gusenleitner as *P. gallicus muchei*, in coll. CSE) were examined by CSE. They agree with typical *P. bucharensis* by having the mesosternum black and the clypeal ridges present. The male from Kars differs by an all-black mesonotum and by a black medial spot on clypeus. Consequently, we treat “*muchei*” (based on males) as a dark and whitish colour form of *P. bucharensis* and not as a form of *P. dominula* (= former *P. gallicus*). It is probably restricted to mountainous regions of NE Turkey and the Caucasus. This assignment is based on colour and morphology alone and requires verification through sequence data.

According to the description by Gusenleitner (1976), the female paratypes of *P. muchei* differ markedly from typical *P. bucharensis* by a reduction of the pale body colour. The mesoscutum is black and the clypeus has a transverse band. We cannot rule out that the type series of *P. muchei* includes *P. nimpha* specimens. Two non-type females from Kagisman (eastern Turkey, collected by CSE together with the above-mentioned male) belong to two species: one to *P. bucharensis* (typical form) and other to *P. nimpha* (with extreme extended pale colour pattern: clypeus and genae all yellow, however, with the mesoscutum and the hypopygium (sternite VI) all black.

Based on the material that was available for us it appears that two taxa of this lineage occur in eastern Turkey and the Caucasus: *P. bucharensis* and *P. nimpha*. Males can be recognized easily by morphological characters (see key to species), whereas the identification of females is hampered by an extraordinary colour variation (extreme pale forms occur together with extreme dark forms). They can be distinguished by characters given in the key (mainly by colour of the hypopygium (sternite VI): yellow in *P. bucharensis*, mainly black or reddish in *P. nimpha*). In addition, the sculpture of the lower half of the mesopleuron is coarser in *P. bucharensis* than in *P. nimpha* (where it is finer, with overlap in a few specimens). Results of DNA barcoding of females is needed to confirm this hypothesis. Another problem is a white coloured form of *P. nimpha* in Iraq (“*P. nimpha irakensis*”, see discussion under *P. nimpha*).

The MFNB houses a large series of females from West Pamir, collected in 1928 by Reinig. This taxon is darker than typical *P. bucharensis* from Bukhara or Turkey (yellow band on gena of female medially largely interrupted, mesoscutum black, tergites with narrow pale bands, and pale colour whitish instead of yellow). The specimens have the clypeus all whitish yellow, the clypeal disc is punctate with large punctures and has some bristles. They probably also represent a dark high-elevation form of *P. bucharensis*. The colour variation of Central Asian specimens is not understood well and requires examination of more material.

Three barcoded specimens from Crete were assigned a separate BIN (Tab. 1). Their colour pattern is intermediate between *P. bucharensis* and *P. dominula* (see description below) but genetically they are most similar to *P. bucharensis* from Cyprus. Therefore, we treat them as an island form of *P. bucharensis*. It is possible that the population from Crete represents a distinct species but further research is required to clarify the taxonomic status of the involved species.

The specimens barcoded fall into four clusters that were assigned three different BINs (Suppl. material 2: NJ tree). The species shows distinct geographic subclustering with specimens originating from Crete, Cyprus, and Azerbaijan. Specimens from Crete differ morphologically distinctly from those of Cyprus (see discussion above), whereas two of the examined specimens from Azerbaijan are similar to the specimens from Cyprus.

A single specimen from Azerbaijan has been assigned a different BIN, whereas another specimen from Azerbaijan that is morphologically similar to the remaining females of *P. bucharensis* agrees genetically with *P. dominula* from Central Europe (Suppl. material 2: NJ tree). More specimens from this region need to be examined to be able to assess the morphological variation of each potential species in this group and its status in relation to *P. dominula*. It is probable that several other genetically distinct taxonomic units of this *P. dominula/P. bucharensis* species complex occur in this region.

####### Diagnosis.

*Polistes bucharensis* is similar to *P. dominula*, and both sexes can be distinguished by the continuous wide yellow band on the temple (gena); seen in lateral view it is more than half as wide as the temple and extends along the entire posterior margin of the eye (specimens from Crete are different, see below). In *P. dominula*, this band is medially interrupted and less than half as wide as the temple, rarely continuous but then it is constricted medially. In females, the yellow band above the antennae is always connected with the band along the inner eye margin. This band is isolated from the lateral bands in *P. dominula* and it does not reach the inner eye margin.

Females of *P. bucharensis* have an entirely yellow clypeus and a somewhat denser pilosity on the clypeus (the pilosity concerns the dark bristles on the clypeal surface). In females of *P. dominula* the colour of the clypeus is variable: entirely yellow (mainly in specimens from Central Europe) or with a black medial spot in 50-70% of specimens from southern Europe and western Turkey (fig. 38/39).

Sternite II is predominantly black with narrow apical yellow band in *P. bucharensis*, the visible part of the remaining sternites is entirely yellow (except in specimens from Cyprus, which have a larger basal part of sternite III black). In *P. dominula*, the visible base of the sternites III-V is always black and the apical yellow band is 0.5-0.7x as wide as the total visible part of the sternite. Additionally, the species can be recognised by the sculpture of the mesopleuron and the lateral face of the propodeum that is markedly coarser in females of *P. bucharensis* compared to *P. dominula*.

Males of *P. bucharensis* have the mesosternum always all black, whereas the mesosternum of *P. dominula* males is partly or entirely yellow. The mesosternum has at least two triangular yellow spots subapically (except in specimens from southern Greece, see below). The colour pattern of the sternites is more variable than in females. For distinction from *P. nimpha* and from species of higher mountains in Central Asia see the key to species and the discussion above.

####### Variations.

Females from Crete (n = 6) differ from typical *P. bucharensis* by a reduction of the yellow body colour. The yellow band of the temple (gena) in lateral view is medially widely interrupted and the clypeus has nearly always a transverse band or medial spot (Fig. 35), except one female with entirely yellow clypeus. The yellow band above the antennal sockets is isolated from both lateral bands. Sternites III-VI are all yellow and sternite II has only a narrow yellow apical band.

The single male that was examined genetically has the mesosternum all black and the yellow band of the temple is medially interrupted. In specimens from Iran the yellow body colour of the only examined male from Arak is replaced by an extreme whitish yellow. For recognition of specimens from Caucasus and E Turkey see discussion on *P. dominula muchei*.

####### Distribution.

From Central Turkey to Central Asia, Israel, Iran and Egypt. In Europe only known from Crete and Cyprus. Specimens described from China and Mongolia (not examined) may also belong to *P. bucharensis* (see Giordani Soika, 1970).

####### Specimens examined.

Europe: Greece (Crete), Cyprus. Asia: Turkey (Diyarbakir, Hakkari, Van, Esendere, Mersin, Göreme, Kars/Kagisman), Iran (Arak/Besril), Azerbaijan, Uzbekistan (Bukhara), Israel (Arava Valley). Africa: Egypt (female, Oasis Dakhla, 2.ix.1992, CSE).

###### Polistes dominula

Taxon classificationAnimaliaHymenopteraVespidae

(Christ)

Figs 10–12

Vespa dominula Christ, 1791, Naturgesch. Insect.: 229–232 + Taf. 21, fig. 1. – Types (♀,♂) lost, type locality: “Kronenberg an der Höh” [Kronberg im Taunus], Germany.
Polistes italica Herrich-Schäffer, 1840, Nomencl. Entomol. 2: 196. – Nomen nudum.
Polistes pectoralis Herrich-Schäffer, 1841, Fauna Insect. German. 179: 39 – Type (♂) lost, type locality: Italien [Italy].
Polistes gallica var. *Lefebvrei* Guérin, 1844, Iconographie du règne animal de G. Cuvier 3: 447 + Pl. 72, Fig. 6. – type repository unknown, type locality: Egypt.
Polistes maculatus Rudow, 1889, Societas Entomologica 3: 171. – Uncounted syntypes (type depository unknown), type locality Smyrna [Izmir], Turkey. The nest (“befestigt an einem Schilfrohrstengel” [attached on a reed stalk]) from where the syntypes were taken puts this synonymy in doubt.
Polistes Merceti Dusmet, 1903, Memorias de la Sociedad Española de Historia Natural 2 (3): 146 (key), 149. – Holotype male (not examined), type locality: Los Molinos, Madrid (Spain).
Polistes gallicus var. rufescens du Buysson, 1912, Annales d’Histoire Naturelle Délégation en Perse II. Entomol. 1: 79. – No type designated, neither type locality nor type depository mentioned. No specimens labelled as “Polistes gallicus var. rufescens” exist at the MNHN (Paris).

####### Remarks and genetic data.

*Polistes dominula* and the reinstated closely related species *P. bucharensis* show high levels of variation in their colour patterns. Previously, both taxa were treated as a single species (Gusenleitner 1976, Carpenter 1996). Our study revealed the presence of six genetic clusters for the two species, all of which were assigned different BINs by the BOLD system, viz. 1) Morocco, 2) SW to Central Europe (subsequently referred to as western cluster), 3) SE to C Europe (subsequently referred to as eastern cluster), 4) Crete, 5) Cyprus to Azerbaijan, and 6) Azerbaijan (Table 1, Suppl. material 2: NJ tree with clusters 1–6 indicated by numbers next to each cluster). In both, the neighbour-joining and the phylogenetic analysis (Fig. 58, see below), *Polistes bucharensis* is nested within the *P. dominula* cluster.

**Figure 57.**
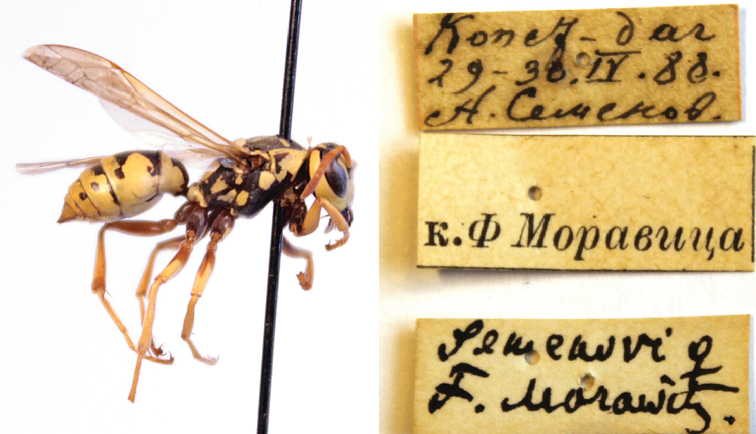
*Polistes semenowi*. Lectotype female, habitus, lateral aspect, and labels. Photo: K. Samartsev.

**Figure 58.**
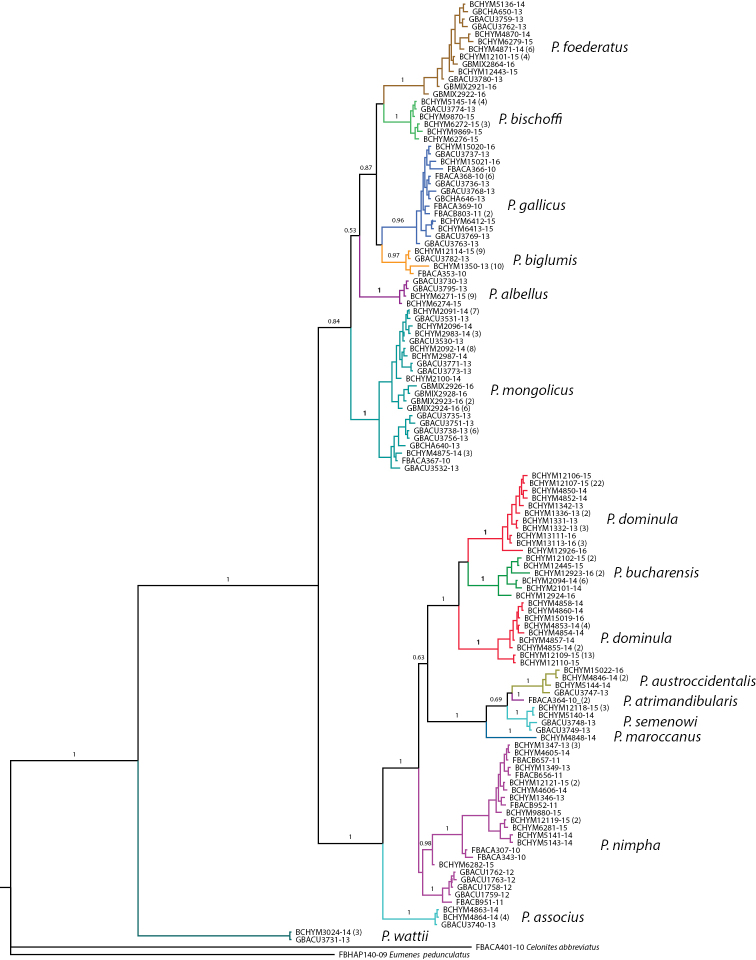
Phylogenetic tree resulting from Bayesian analysis of COI sequence data. Number in brackets after terminal branch labels (Process ID) indicate number of haplotype sequences. Numbers on branches denote posterior probability values (omitted for branches within species).

Two of these clusters (2 and 3) occur in Europe. The current data indicate for cluster 2 a south-western European distribution although we could only examine one specimen from France and records from Spain still are missing. The cluster occurs over whole Germany and it is close to the cluster of Morocco (1). The other European cluster (3) includes specimens from Greece and Azerbaijan and seems to have a more south-eastern distribution. It covers whole Germany with the westernmost records from the Aosta valley in the Italian Alps. This cluster is genetically closer to *P. bucharensis* than to the *P. dominula* clusters (1) and (2) from the western area and consequently we treat specimens from Crete as *P. bucharensis* (see above).

The high intraspecific variation (Table 1) and presence of multiple BINs could be the result of several species being present. However, specimens from NW Africa and Europe are very similar and indistinguishable by morphology or colour pattern. Furthermore, Neumeyer at al. (2014) found no sufficient differences in the ITS1 gene between the European clusters 2 and 3. We therefore refrain from describing each cluster as a new species until further evidence is available.

*Polistes bucharensis* with its eastern clusters (3–6) is genetically closer to the eastern clusters of *P. dominula* (1, 2). However, specimens of *P. bucharensis* are clearly separated by colour pattern and male morphology from *P. dominula*, supporting the notion that they represent distinct species.

The population of Crete seems to be isolated from the remaining populations for a long period, and the common ancestor probably came from the *P. bucharensis* lineage.

Because of this result, we follow the concept of morphospecies here and treat *P. bucharensis* as a valid species, separated from *P. dominula* s. str. by genetics, morphology, and colour pattern.

####### Diagnosis.

*Polistes dominula* is the most common *Polistes* species in Europe. The female is characterised by a mostly or entirely yellow sternite VI in combination with an orange apical half of the antenna. The yellow band on the temple behind the eye in lateral view is interrupted in nearly all examined specimens, except in some from southern Greece and from Tunisia. The clypeal pattern is variable. Populations from Central Europe usually have an all yellow clypeus, most specimens from southern Europe and some from southern Central Europe (e.g. southern Germany) have one or two black medial spots, or a band on the clypeus medially (Figs 38, 39).

The female of *P. dominula* can be confused with *P. associus* (see key to species) and with *P. bucharensis* in western Asia. *Polistes bucharensis* always has a completely yellow clypeus in combination with a continuous band on the temple behind the eyes. This band is interrupted in most specimens of *P. dominula*, and the clypeus has often (about two-thirds of examined specimens) black spots or a band in females from southern Europe. For specimens from Crete, see under *P. bucharensis*.

The male of *P. dominula* is characterised by the orange apical half of antenna in combination with the lack of any impressions or ridges on the clypeus. It can be distinguished from the similar *P. bucharensis* by a (partly or entire) yellow mesosternum; the latter is all black in *P. bucharensis*. Also, the clypeus is laterally somewhat bulging in *P. bucharensis*, and always without any elevations in *P. dominula*.

Some specimens of *P. dominula* (identity confirmed by barcoding) from the Peloponnese (Greece) have some colour similarities of typical *P. bucharensis* (females with yellow clypeus and with wide yellow band on temple, males with mesosternum entirely black) and resemble *P. bucharensis*. However, the yellow band on the temple is medially constricted, and the yellow band above the antennal sockets is isolated from both lateral bands. In addition, the mesopleural sculpture is finer than that of typical *P. bucharensis*. We therefore regard these specimens as pale form of *P. dominula* at the SE border of its distribution area. The specimens from Greece occur together with typically coloured *P. dominula* (females: clypeus with large black spot or band, band on temple interrupted; males: mesosternum partly or all yellow). A female from Tunisia (Dougga) agrees in colour pattern with the above-described pale females from Greece and likely belongs to the same whitish form of *P. dominula*.

####### Distribution.

NW Africa, C and S Europe as far north as Latvia, but missing in Great Britain, Scandinavia, Crete and Cyprus. Introduced to Australia, North America (including Canada, Buck et al. 2008) and South America. The species has also been recorded from central and eastern Palaearctic regions and from India (e.g. Guiglia 1972). These records need confirmation, because they may belong to *P. bucharensis*. The easternmost records that we could examine are from western Turkey and from Azerbaijan. However, it can be expected that the species occurs farther east in Russia or Central Asia. One examined female from Egypt is clearly *P. bucharensis* and *P. dominula* probably does not occur in NE Africa.

####### Specimens examined.

Europe: Examined from most countries in Central and S Europe. Asia: Turkey (Termessos/Antalya), Azerbaijan. Africa: Morocco, Tunisia.

###### Polistes foederatus

Taxon classificationAnimaliaHymenopteraVespidae

Kohl, sp. restit.

Fig. 13

Polistes foederata Kohl, 1898, Annalen des kaiserlich-königlichen Naturhistorischen Hofmuseums, Wien 13: 90 + Taf. III. – Lectotype male (NHMW, examined by RN & CvA), designated by Blüthgen (1943: 129), type locality “Transkauk., Helenendorf” [Göygöl], Azerbaijan. **Sp. restit.**
Polistes foederata var. obscuricornis Mader, 1936, Entomologische Zeitschrift (Frankfurt a.M.) 50 (23): 263. – 2 syntype females (NHMW, examined by RN & CvA), type locality: Insel Krk [island of Krk], Croatia, but the name is not available (A. Ćetković, in litt.).

####### Remarks.

The species is widespread in N Italy, the Balkans, and western Asia (Turkey to Caucasus area). The identity of *P. foederatus* remained unclear for a long time, and in the past the species was usually treated as *P. gallicus*. Arens (2011) was the first to recognize two species of the *P. gallicus* group in Greece (*P. gallicus* and *P. hellenicus*), using the length of the malar space as a new diagnostic character. However, he interpreted specimens with long malar space as “*P. gallicus*” but ignored that true *P. gallicus* from the western Mediterranean usually have a short malar space (with some exceptions). Type examination and genetic analysis clearly show that *P. gallicus* sensu Arens (2011) from Greece belong to *P. foederatus*.

####### Diagnosis.

*Polistes foederatus* is unique in the *P. gallicus* species group by possessing the longest malar space of all species combined with a large and mainly rectangular and central black spot on the clypeus in females. In addition, the dorsal side of the flagellum is often slightly darkened. However, especially females can be confused with *P. gallicus* in the transition zone of both species (N Italy to Balkans), because the latter rarely has an extreme long malar space (some genetically examined specimens from the Italian Alps). The flagellum is completely reddish in *P. gallicus*, but always darkened dorsally in alpine *P. foederatus*. The yellow spots on the mesoscutum are usually lacking in smaller specimens from Croatia and Italy. The male is variable in colour pattern; the mesoscutum is black or has a pair of large yellow spots and the base of tergite II is either black or largely yellow.

####### Distribution.

From NE Italy to Greece and Azerbaijan. Widespread and common in mainland Greece (Arens 2011, as *P. gallicus*).

####### Specimens examined.

Europe: Italy (Trentino/Rovereto, Lombardia/Valtellina/Grossio, Veneto/N of Verona), Croatia (Krk), Greece (Crete/Matala, Peloponnese), Cyprus (Akrotiri). Asia: Turkey (Antalya), Azerbaijan.

####### Genetic data.

Specimens of *Polistes foederatus* from several countries, including Azerbaijan, where the type locality is situated, were analysed genetically. The species exhibits little genetic variation and all specimens share the same BIN. The specimens from Crete form a distinct subcluster, perhaps because of longer isolation. However, the specimens from Crete are closer to specimens from the European and Asian mainland than Cretan *P. bucharensis* are from their mainland populations.

###### Polistes gallicus

Taxon classificationAnimaliaHymenopteraVespidae

(Linnaeus)

Figs 14 , 15

Vespa gallica Linnaeus, 1767, Systema Naturae Ed. 12, 1 (2): 949 – Holotype male (LSL, examined by RN), type locality “Europa australi” [S Europe].
Polistula omissa Weyrauch, 1938, Arbeiten über physiologische und angewandte Entomologie aus Berlin-Dahlem 5 (3): 277 – Lectotype male (lost; see Arens 2011: 462), designated by Weyrauch 1939, Archiv für Naturgeschichte, Neue Folge 8 (2): 161, type locality: Marseille, France, mentioned in Weyrauch (1939: 161).

####### Remarks.

The name *P. gallicus* (sensu lato) was used in the past for three Mediterranean species: *P. foederatus*, *P. mongolicus*, and *P. gallicus*. A reassessment of morphological characters in combination with DNA barcoding shows that *P. gallicus* (sensu stricto) is a valid species with a mainly western Mediterranean distribution (eastwards to Corfu, but probably very rare on Balkans). *Polistula omissa* is regarded as a synonym of *P. gallicus* (sensu stricto).

####### Diagnosis.

*Polistes gallicus* females are characterised by a short malar space (but in a few specimens from Italy as long as in *P. foederatus*) and by two yellow spots on the mesoscutum; these spots are absent in most females of *P. mongolicus*. If there is a dark patch on the clypeus, it is small (rounded or forming a transverse band) and situated on the apical half of the clypeus. The posterior pronotal band is variable, short (in most specimens from Portugal) or reaching the anterior pronotal transverse band (in most specimens from N Italy). In the transition zone to *P. mongolicus* (N Italy, Balkans), *P. gallicus* can be confused with *P. mongolicus* when the yellow mesonotal spots are absent (one barcoded female of *P. gallicus* from Italy, Lombardia, with reduced, minute yellow spots only). *Polistes gallicus* has the posterior stripes connected to the anterior transverse band of the pronotum, whereas it remains separated from the pronotal band in all examined *P. mongolicus* from Croatia. Males can be recognized by the combination of the short malar space and two yellow spots on the mesoscutum.

*Colour variation.* All examined females from NW Africa have the clypeus yellow (one female with minute black spot), the mesoscutum has a pair of large yellow spots, and the tergite VI is entirely black (apical half yellow in one specimen). Males from NW Africa were not examined.

####### Distribution.

Western and central Mediterranean area, eastwards to the Greek island of Corfu. In NW Africa from Tunisia to Morocco. Northwards to Italian Alps and S Switzerland. Specimens from Egypt and Israel formerly assigned to *P. gallicus* belong to *P. mongolicus*.

####### Specimens examined.

Europe: Croatia (Istria), Greece (Corfu), Italy (Lombardia/Brescia, Veneto/N. of Verona, Pavone, Dro, Sardinia), Spain (Mallorca/Alcúdia, Andalucía/various locations), France (Bouches du Rhône/Alpilles, Pont du Diable, La Rouquette), Portugal (Algarve/Carrapeteira), Malta (Ghajn Tuffieha, female, photo from Kristofer Mogyorossy), Switzerland. Africa: Algeria (Alger), Morocco (Sefrou, Ht Atlas), Tunisia (Le Kef).

####### Genetic results.

Specimens from *Polistes gallicus* originating from several European countries between Croatia and Portugal and from Morocco were examined genetically. They exhibit some genetic variation but all specimens share the same BIN.

###### Polistes maroccanus

Taxon classificationAnimaliaHymenopteraVespidae

Schmid-Egger sp. n.

http://zoobank.org/BCA5FEA8-A64F-48AD-8203-7350DE1BC870

Figs 16 , 46–49 , 50–56

####### Type specimens.

Holotype♀, Morocco, Haut Atlas, 2 km N Tizi n’Tichka, 31.289°N, 7.381°W, 2150 m 13.vi.2014, (leg. Schmid-Egger, voucher ID: BC ZSM HYM 22043, ZSM). Specimen with right antenna and right fore leg missing. Paratypes: 1♀, Ifrane env. 9.v.1997, (leg. Denes jun., coll. OLL). 1♀ (RN0664), Asni (1250 m), 3-11 vii 1934 (A. Ball leg., ETHZ).

####### Diagnosis.

*Polistes maroccanus* sp. n. is close to *P. atrimandibularis* and can be distinguished by the characters given in Table 2.

####### Description.

FEMALE. Holotype, body length 14 mm; fore wing length 11.5 mm.

For colour pattern see figures.

*Head*. Mandible with a large depression on its outer face; both ridges of mandible narrow, medially 0.15 times as wide as mandibular diameter, remaining space shiny, with a few large punctures on upper third; malar space 1.5 times POL. Clypeus slightly wider than long (length/width ratio 0.92 in holotype, 0.86 in paratype).

*Mesosoma*. Posterior half of pronotum obliquely rugose and with short pubescence, only medio-anteriorly with longer setae; change in sculpture between mesepisternum and epicnemium abrupt (=“epicnemial ridge distinct”), mesepisternum densely rugulose and epicnemium only superficially micro-sculptured; propodeum coarsely transversely striate. fore wing including pterostigma and veins reddish brown.

*Variations*. Body length of paratypes similar to holotype. Left mandible of one paratype with small yellow spot, and clypeus all yellow in one paratype.

**Table 2.** Characters for separating *P. maroccanus* from *P. atrimandibularis* (females only, HT = Holotype, PT = Paratype).

	
Lower ridge of mandible narrow, in HT= 0.22× (in PT= 0.24×) as large as mandible (measured medially)	Lower ridge of mandible wider, 0.37× as wide as mandible
Both ridges of clypeus smaller, upper ridge in PT nearly flat and barely expressed	Both ridges of clypeus wider
Medial impression of mandible shiny, with very fine microsculpture	Medial impression of mandible dull, with coarser microsculpture
Lower margin of clypeus (= near margin of yellow colour) ridged, shiny	Lower margin of clypeus straight, with microsculpture
Mesoscutum in HT medially with two minute yellow spots, black in PT	Mesoscutum all black
Black clypeal spot band-shaped, small in holotype, missing in paratypes	Black clypeal spot larger

####### Distribution.

Only known from the High and Middle Atlas Mountains in Morocco. Previous to this study, CSE identified a female from Quirgane (High Atlas, 22.v.1995, leg. et coll. M. Hauser) as *P. atrimandibularis*. It is probably referable to *P. maroccanus* as well but the specimen was not available for re-examination.

####### Biology.

The species is most probably a social parasite. At the type locality, it was collected together with *P. dominula* that is most probably the host.

####### Etymology.

*Polistes maroccanus* is named after the country of origin, Morocco.

####### Remarks.

DNA barcoding of a specimen from Morocco, formerly identified as *P. atrimandibularis*, indicated that it belongs to a different species that is close to the previously known social parasites. A detailed morphological examination resulted in some different character states and supports the notion that the Moroccan specimens belong to a new species. The species is morphologically close to *P. atrimandibularis* and probably replaces it in NW Africa.

###### Polistes mongolicus

Taxon classificationAnimaliaHymenopteraVespidae

du Buysson stat. rev.

Figs 17–19

Polistes gallicus var. mongolicus du Buysson, 1911, Bulletin du Muséum National d‘Histoire Naturelle 76: 218 – Syntypes males, females (MNHN, ZISP, male from MNHN examined by RN & CvA, hereby designated as lectotype by CvA), type locality: road from Kuqa [“Koutchar”] to Karashahr [“Karachar”], China (Xinjiang autonomous region), ix.1909. **Stat. rev.**
Polistes omissus var. ordubadensis Zirngiebl, 1955, Mitt. Münchner Entomol. Ges. 44/45: 381. – Holotype female (ZSM, examined by RN & CvA), type locality: Ordubad, Azerbaijan. **Syn. n.**
Polistes omissus kaszabi Giordani Soika, 1970: 327–328 – Holotype female (HNHM, examined by RN), type locality “Duusch ul” near Züünkharaa [“Zuun-Chara“], Mongolia. Synonymy tentative.
Polistes hellenicus Arens, 2011: 464 – Holotype male (coll. Werner Arens, examined by RN), paratype (examined by CvA), type locality: Ano Kotili, Greece. **Syn. n.**

####### Remarks.

The species is widespread in SE Europe to C Asia and China. Apart from the original description it was later described as P. omissus var. ordubadensis Zirngiebl from Caucasus and as *P. hellenicus* from Greece by Arens (2011). Arens (2011) was the first who recognized two different species of the *P. gallicus* species group in Greece and he described *P. hellenicus* as new species. He based his description mainly on the short malar space in contrast to *P. foederatus* with long malar space, and the black venter of the males (yellow in *P. foederatus*). Morphological comparison, genetic examination of specimens from a wide geografic range and type study confirms the conspecificity of *P. hellenicus* and *P. ordubadensis* with *P. mongolicus*. Our material increases the known range of the species from Croatia to Central Asia and China, and to NE Africa. The examined type specimen of *P. mongolicus* from China is somewhat darker than western specimens, but agrees in general aspects with our species definition. For taxonomic status of *Polistes omissus kaszabi*, see Neumeyer et al. (2014).

####### Diagnosis.

Within the *P. gallicus* group the female of *P. mongolicus* is characterized by a short malar space, the lack of yellow spots on the mesoscutum (present in some females from Greece and western Asia), and usually by a yellow clypeus. Some females mainly from Greece have a very small to a medium-sized transverse spot on the clypeus. See Arens (2011, as *P. hellenicus*) for discussion of the colour variability. *Polistes foederatus* has longer malar space (see key to species).

The recognition of *P. mongolicus* is not problematic in Greece and farther east, but on the Balkans females may be confused with *P. gallicus* (see diagnosis of the latter). Males of *P. mongolicus* occur in two different colour forms. Specimens from Europe usually have the mesosternum entirely black or with a pair of yellowish spots, whereas the mesosternum of males from Asia and Egypt is largely yellow. Recognition of European males is therefore unambiguous.

In N Africa *P. mongolicus* is restricted to Egypt, whereas *P. gallicus* occurs in Tunisia, Algeria and Morocco. Specimens from Libya were not examined, but it cannot be ruled out that ranges of both species overlap in this region.

*Colour variations.* All examined females from Egypt have a yellow clypeus, with at most a minute black medial spot; the hypopygium (sternite VI) is partly yellow or reddish; one of the females has a pair of minute yellow spots on the mesoscutum.

####### Distribution.

Balkans from Croatia to Greece, east to Central Asia, Mongolia, and China, south to Israel and Egypt.

####### Specimens examined.

Europe: Croatia, Serbia, Macedonia, Greece, Cyprus. Asia: Turkey (Antalya, Hakkari), Israel (Jordan Valley), Azerbaijan, China. Africa: Egypt (Kairo; Al Fajum).

####### Genetic results.

Specimens from Croatia, Greece, Turkey, Cyprus, and Azerbaijan were analysed. *Polistes mongolicus* shows some genetic divergence, mainly between specimens from Cyprus, from Asia and from Europe, with a mean intraspecific distance of 0.88% and a maximum intraspecific distance of 2.04% (Table 1). They all share the same BIN.

###### Polistes nimpha

Taxon classificationAnimaliaHymenopteraVespidae

(Christ)

Figs 20–23

Vespa nimpha Christ, 1791, Naturgesch. Insekt.: 232. – Types (female, male) lost, type locality: Kronberg, Taunus (Germany).
Vespa diadema Latreille, 1802, Ann. Mus. Hist. Nat. 1: 292, nec Christ, 1791. – Type (female) lost, type locality: surroundings of Paris (France).
Polistes opinabilis Kohl, 1898, Ann. Naturh. Hofmus., Wien 13: 90 + Taf. III. – Lectotype male (NHMW, examined by RN & CvA) designated by Blüthgen (1943: 127), type locality: Frain [= Vranov], Moravia (Czech Republic).
Polistes nimpha var. *Moltonii* Guiglia, 1944, Atti d. Soc. Italiana di Sc. Nate del Museo Civico di Storia Naturale in Milano 83: 166. – Holotype female (MSNM, not examined), type locality: Spotorno, Liguria (Italy).
Polistes nimpha irakensis Gusenleitner, 1976: 119. – Holotype male (ZSM, examined by CSE), type locality “Hashimiya, Irak”. Female paratype from Abu Ghureib, Iraq, (ZSM, examined by CSE).

####### Remarks.

*Polistes nimpha* is well defined by male morphology, in particular the long apical antennal segment and distinct lateral ridges of the clypeus, and in the female by the colour pattern (European specimens only). In western Asia, the recognition of females is not always easy since the species varies markedly in colour pattern. It can be confused with *P. associus* (lowlands of Turkey, Israel) and with *P. bucharensis* (eastern Turkey, Caucasus region, Iraq). In a small geographic area in western Asia, the dark and the pale coloured form occur in close vicinity, but probably not sympatric. Especially specimens from Iraq have an extended yellow colour pattern and can be confused with *P. bucharensis*. They can be recognised by the colour of the hypopygium (=sternite VI), but identification of some females remains difficult.

Differences in the ocellar angle (more obtuse in *P. nimpha/dominula* than in *P. associus*), as stated by Arens (2011), cannot be confirmed here. The sculpture of the lower half of the mesopleuron is somewhat coarser in *P. nimpha* than in *P. associus*, although both species overlap in this character.

####### Diagnosis.

The most important diagnostic character of *P. nimpha* females is the shape of the transverse pronotal band in that it is narrow and pointed ventrally. The lateral portion of the transverse band (seen in lateral view) is wider in front of the pronotal carina than behind it. In the remaining non-parasitic species of the *P. dominula* species group (*P. associus*, *P. dominula*, and *P. bucharensis*) the portion of the yellow band behind the carina is always wider. However, some extremely xanthic females of *P. nimpha* from western Asia also possess a very wide pronotal band. About 70% of females from western Asia have paired yellow drop-shaped spots on the mesoscutum. These spots are usually absent in European specimens. The visible part of the hypopygium (sternite VI) is usually black or partly reddish in *P. nimpha* and also in *P. associus*, rarely with a yellowish apical spot, while the hypopygium is entirely or predominantly yellow in *P. dominula* and *P. bucharensis*.

The latter character is used here for recognition of *P. nimpha* and *P. bucharensis* in eastern Turkey. This character is helpful in distinguishing xanthic *P. nimpha* females (i.e., with an all-yellow clypeus and temple), which are otherwise similar to *P. bucharensis*. Often only a combination of a several characters will ensure a correct identification of western Asian specimens.

The separation of *P. associus* and *P. nimpha* females can also be difficult, especially in areas where both species occur sympatrically (e.g. in western Croatia). The colour pattern of *P. associus* is diagnostic and exhibits little variation (based on specimens identified by barcoding): Transverse pronotal band wide laterally, separated from posterior band by 2-3 times the diameter of the anterior ocellus; mesoscutum with two large drop-shaped yellow spots. Despite significant variation, western Asian *P. nimpha* never show this combination of characters. The hypopygium colour is variable in both species but never all red in *P. nimpha* as it sometimes is in *P. associus* (one female from Israel).

####### Distribution.

Europe, north to S Finland, Palaearctic Asia east to Mongolia, China, and Russian Far East.

####### Specimens examined.

Europe: Germany, Italy (Alps), Bulgaria, Greece, France, Croatia, Portugal, Switzerland. Asia: Iraq, Turkey (Kars/Kagisman, Hakkari region, Denizli/Pamukkale, Antakya, Alanya, Marmaris, Diyarbakir).

####### Genetic results.

Only specimens from Central Europe were examined genetically, except for one specimen from Greece. The species shows significant intraspecific genetic variation. It is possible that the examination of Asian species will yield unexpected results.

###### Polistes semenowi

Taxon classificationAnimaliaHymenopteraVespidae

Morawitz

Figs 24 , 57

Polistes semenowi Morawitz, 1889, Horae Entomol. Soc. Ross. 23: 552. – 3 female syntypes (ZISP, photo of 1 female examined by CvA who hereby designated it as lectotype), type locality: Copet-dag [Kopet Dag], S Turkmenistan. The illustrated female (labelled: “Copet-dag, 29-30.iv.[18]88, A. Semenov”, “K.F. Morawitza”, “*semenowi* F. Morawitz”) is the lectotype.
Polistes sulcifer Zimmermann, 1930, Mitt. Zool. Mus. Berlin 15: 610. Holotype male (MFNB, examined by CSE), type locality: Mendel-Penegal, Südtirol (N Italy). **Syn. n.**
Pseudopolistes sulcifer var. similator Zirngiebl, 1955, Mitt. Münchner Entomol. Ges. 44/45: 384. Holotype female (ZSM, examined by CvA and CSE), type locality: Ordubad [19]13, leg. Klar. **Syn. n.**
Sulcopolistes sulcifer auctt. (e.g., Guiglia 1972).

####### Remarks.

The species was formerly treated as *P. sulcifer* by authors. See also comments under *P. austroccidentalis* for the nomenclature and taxonomy of this species.

####### Diagnosis.

The species can only be recognized based on the shape of the mandible, clypeus, and the colour of male fore and mid coxae. The upper ridge of the mandible is markedly modified in the female and forms a triangle in dorsal view (weaker and more rounded in male). The recognition of males is more difficult because the upper ridge is sometimes only weakly curved and resembles that of *P. austroccidentalis*. The mandibles (frontal view) differ between both species in that the mandibular depression is narrower in *P. austroccidentalis* and with a wider upper mandibular ridge. Furthermore, the male fore coxa is almost always and the mid coxa usually marked with yellow as opposed to *P. austroccidentalis* where all coxae are black.

####### Life history.

*Polistes semenowi* is a social parasite of *P. dominula* (see Cervo 2006, as *P. sulcifer*), in western Asia probably also of *P. bucharensis*.

####### Distribution.

S and C Europe, north to Germany (Hesse, one record from 1908; Tischendorf et al. 2015 as *P. sulcifer*), east to Central Asia, not recorded from Spain and Portugal. One female in coll. MFNB labelled „Egypt, Ehrenberg leg”] has more yellow coloured hind coxa than usual. Its origin is doubtful as is that of an *atrimandibularis* specimen with the same data (see discussion under that species). No other specimens from North Africa have been examined by the authors. Males are sometimes found at higher altitudes (e.g., five males from Italy, Dolomiti, Rif. Coldai at 2150 m; 2 males from Greece, Mt. Olympus at 2200-2500 m, in coll. CSE).

####### Specimens examined.

Europe: France, Croatia, Italy (Alps, Abruzzi, Calabria, Sicily), Greece, Macedonia, Montenegro, Serbia, Switzerland. Asia: Azerbaijan, Turkey (Uludağ, Bursa, Van), Syria, Egypt, Turkmenistan.

####### Genetic data.

Barcoded specimens from south-central Europe showed little genetic variation.

##### Other subgenera

The following species are only discussed briefly, because they were either introduced only recently or they occur near the southern border of the study area. For further information on the nomenclature of these species see Carpenter (1996). For identification of the subgenus Gyrostoma and other Asian species see Richards (1984a) and Tan et al. (2014).

###### Subgenus Aphanilopterus Meunier, 1888

####### Polistes major

Taxon classificationAnimaliaHymenopteraVespidae

Palisot de Beauvois

Polistes major Palisot de Beauvois, 1818, Insect. Recueill. Afrique Amérique, livr. 12: 206.

######## Distribution.

The nominate subspecies of *Polistes major* has been found in northern Spain (Castro et al. 2013). The species ranges from the southern U.S.A. to Brazil and Peru, including several Caribbean islands (Carpenter 1996).

###### Subgenus Gyrostoma Kirby, 1828

####### Polistes olivaceus

Taxon classificationAnimaliaHymenopteraVespidae

(DeGeer)

Vespa olivacea DeGeer, 1773, Mem. Hist. Insect. 3: 582, pl. 29 fig. 9.

######## Distribution.

The species is recorded from E Africa, S Asia and Australia (Carpenter 1996). Richards (1984b) mentions records from Oman.

####### Polistes wattii

Taxon classificationAnimaliaHymenopteraVespidae

Cameron

Polistes wattii Cameron, 1900, Ann. Mag. Nat. Hist. (7) 6: 416.

######## Distribution.

The species is recorded from SW and S Asia (Arabian Peninsula, Iran, India, and S China) according to Richards (1984b) and Carpenter (1996).

### Results of phylogenetic analysis and discussion

The phylogenetic analysis based on Bayesian inference resulted in a split of the included *Polistes* species into two major clades, with a posterior probability support value of 0.84 for the *P. gallicus* group (including *P. foederatus*, *P. bischoffi*, *P. gallicus*, *P. biglumis*, *P. albellus*, and *P. mongolicus*), and with a branch support of 1.0 for the *P. dominula* species group (including *P. dominula*, *P. bucharensis*, *P. austroccidentalis*, *P. atrimandibularis*, *P. semenowi*, *P. maroccanus*, *P. nimpha*, and *P. associus*) (Fig. 58). *Polistes dominula* is represented by two clades with *P. bucharensis* in between, illustrating the need for a closer examination of the status of the disjunct *dominula* clades. The sister group of the *dominula*/*bucharensis* clade is composed of a well-supported (pp = 1.0) clade consisting of the four social parasite species *P. atrimandibularis*, *P. austroccidentalis*, *P. maroccanus, and P. semenowi* and (Fig. 58), supporting the notion of a single origin of their biology. Within the *P. gallicus* species group there is some support for a clade composed of *P. foederatus*, *bischoffi*, *gallicus*, and *biglumis* (Fig. 58).

These results concur with an earlier analysis of a mitochondrial gene fragment (16S ribosomal RNA) by Choudhary et al. (1994). In their analysis, the three examined social parasites form a monophyletic group that is nested within other European *Polistes*. Unlike their study, where the parasitic clade came out as the sister group to *dominula*+*nimpha*, our analysis yielded the *dominula*/*bucharensis* clade as the sister group, albeit with low branch support (0.63, Fig. 58). *Polistes nimpha*, on the other hand, came out as the sister group to the clade comprising of *P. dominula*/*bucharensis* and the social parasites. This sister group relationship is supported by a robust branch support of 1.0 (Fig. 58). Based on the results of the present analysis, the following species groups are proposed within the subgenus Polistes.

The most important change affected the two species *P. associus* and *P. biglumis*. *Polistes associus* was formerly treated as a member of the *P. gallicus* species group (= subgenus Leptopolistes sensu Guiglia 1972) because of the narrowed temples of the male – a typical character of this subgenus, according to former authors. However, females of *P. associus* are morphologically and genetically close to *P. dominula*/*P. bucharensis* and *P. nimpha*, and can easily be confused with the latter species, providing support for placing the species in the *dominula* species group.

**Table 3.** Species and species groups of *Polistes* based on the present study and compared to Guiglia (1972). (Sg. = subgenus, * social parasite)

**Species**	**Proposed species group**	**Guiglia (1972)**	**Transferred to**
*albellus*	*gallicus* group	(Sg. *Leptopolistes*)	
*associus*	*dominula *group	Sg. *Leptopolistes*	*dominula *group
atrimandibularis*	*dominula *group	Genus Sulcopolistes*	
austroccidentalis*	*dominula *group	Genus *Sulcopolistes *(as *S. semenowi*)	
*biglumis*	*gallicus* group	Sg. *Polistes*	*gallicus* group
*bischoffi*	*gallicus* group	Sg. *Leptopolistes*	
*bucharensis*	*dominula *group	(Sg. *Polistes*)	
*dominula*	*dominula *group	Sg. *Polistes *(as *P. gallicus*)	
*foederatus*	*gallicus* group	Sg. *Leptopolistes*	
*gallicus*	*gallicus* group	Sg. *Leptopolistes *(as *P. omissus*)	
maroccanus*	*dominula *group		
*mongolicus*	*gallicus* group	(Sg. *Leptopolistes*)	
*nimpha*	*dominula *group	Sg. *Polistes*	
semenowi*	*dominula *group	Genus *Sulcopolistes *(as *S. sulcifer*)	

Likewise, *P. biglumis* was traditionally treated as a member of the *P. dominula* species group (= subgenus Polistes sensu Guiglia 1972), based on the broad, convex temples of the males. The female, however, is very similar to *P. albellus*, and the species has close relationships to the *P. gallicus* species group (= subgenus Leptopolistes sensu Guiglia 1972).

Despite the comparatively limited informative value of a single mitochondrial gene region like the 658bp COI barcode fragment, it still provides insights into phylogenetic relationships within the group and the phylogenetic relationships resulting from the analysis are largely in agreement with inferred by other lines of evidence. A more comprehensive analysis of *Polistes* including additional genetic markers, in particular of nuclear genes (see Neumeyer et. al. 2014), should be employed for evaluating and scrutinising the results of the present study.

## Supplementary Material

XML Treatment for Polistes albellus

XML Treatment for Polistes associus

XML Treatment for Polistes atrimandibularis

XML Treatment for Polistes austroccidentalis

XML Treatment for Polistes biglumis

XML Treatment for Polistes bischoffi

XML Treatment for Polistes bucharensis

XML Treatment for Polistes dominula

XML Treatment for Polistes foederatus

XML Treatment for Polistes gallicus

XML Treatment for Polistes maroccanus

XML Treatment for Polistes mongolicus

XML Treatment for Polistes nimpha

XML Treatment for Polistes semenowi

XML Treatment for Polistes major

XML Treatment for Polistes olivaceus

XML Treatment for Polistes wattii

## Supplementary materials

Supplementary material 1 List of voucher specimens Data type: Microsoft Excel Worksheet (.xlsx) Explanation note: specimens date. This dataset is made available under the Open Database License (http://opendatacommons.org/licenses/odbl/1.0/). The Open Database License (ODbL) is a license agreement intended to allow users to freely share, modify, and use this Dataset while maintaining this same freedom for others, provided that the original source and author(s) are credited.Christian Schmid-Egger, Kees van Achterberg, Rainer Neumeyer, Jérôme Morinière, Stefan Schmidt

Supplementary material 2 NJ tree Data type: Adobe Acrobat Document (.pdf) Explanation note: phylogenic data. This dataset is made available under the Open Database License (http://opendatacommons.org/licenses/odbl/1.0/). The Open Database License (ODbL) is a license agreement intended to allow users to freely share, modify, and use this Dataset while maintaining this same freedom for others, provided that the original source and author(s) are credited.Christian Schmid-Egger, Kees van Achterberg, Rainer Neumeyer, Jérôme Morinière, Stefan Schmidt
