# Supplementary material for: Revision of the West Palaearctic Polistes Latreille, with the descriptions of two species – an integrative approach using morphology and DNA barcodes (Hymenoptera, Vespidae)
Source: Zookeys. 2017 Nov 2;(713):53–112. doi: 10.3897/zookeys.713.11335 (PMC5674218; doi:10.3897/zookeys.713.11335)
Supplement: Supplementary material 2 — NJ tree [file zookeys-713-053-s002.pdf]

BOLD TaxonID Tree

Title	:	Western Palaearctic Polistes [WPPOL]
Date	:	23-November-2016
Data Type	:	Nucleotide
Distance Model	:	Kimura 2 Parameter
Marker	:	COI-5P
Codon Positions	:	1st, 2nd, 3rd
Labels	:	Country & Province, Sex, SampleID, BIN uri
Filters	:	Length > 200
Colorization	:	bin

Sequence Count	:	264
Species count	:	15
Genus count	:	1
Family count	:	1
Unidentified	:	0

BIN Count	:	20

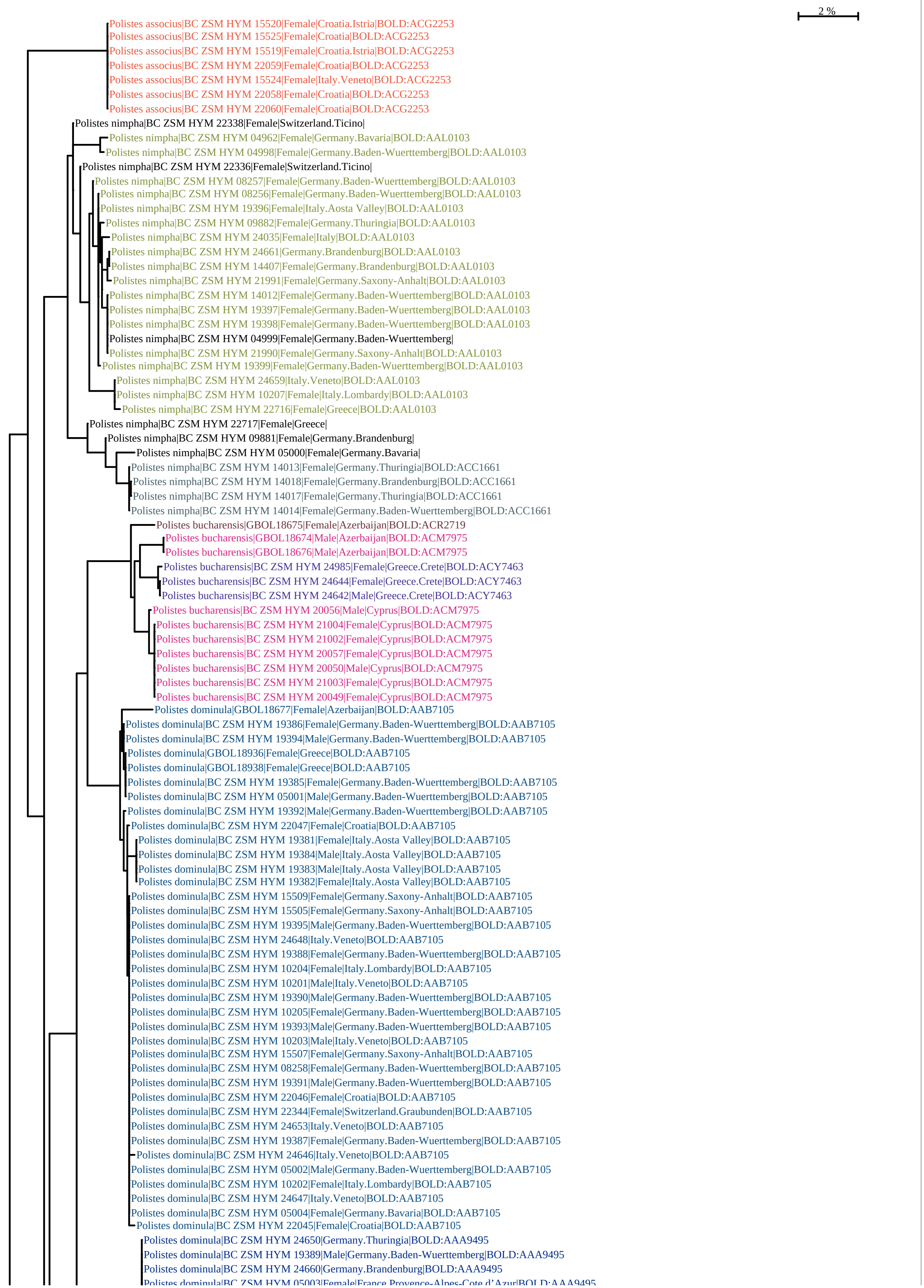
2 %
Polistes associus|BC ZSM HYM 15520|Female|Croatia.Istria|BOLD:ACG2253
Polistes associus|BC ZSM HYM 15525|Female|Croatia|BOLD:ACG2253
Polistes associus|BC ZSM HYM 15519|Female|Croatia.Istria|BOLD:ACG2253
Polistes associus|BC ZSM HYM 22059|Female|Croatia|BOLD:ACG2253
Polistes associus|BC ZSM HYM 15524|Female|Italy.Veneto|BOLD:ACG2253
Polistes associus|BC ZSM HYM 22058|Female|Croatia|BOLD:ACG2253
Polistes associus|BC ZSM HYM 22060|Female|Croatia|BOLD:ACG2253
Polistes nimpha|BC ZSM HYM 22338|Female|Switzerland.Ticino|
Polistes nimpha|BC ZSM HYM 04962|Female|Germany.Bavaria|BOLD:AAL0103
Polistes nimpha|BC ZSM HYM 04998|Female|Germany.Baden-Wuerttemberg|BOLD:AAL0103
Polistes nimpha|BC ZSM HYM 22336|Female|Switzerland.Ticino|
Polistes nimpha|BC ZSM HYM 08257|Female|Germany.Baden-Wuerttemberg|BOLD:AAL0103
Polistes nimpha|BC ZSM HYM 08256|Female|Germany.Baden-Wuerttemberg|BOLD:AAL0103
Polistes nimpha|BC ZSM HYM 19396|Female|Italy.Aosta Valley|BOLD:AAL0103
Polistes nimpha|BC ZSM HYM 09882|Female|Germany.Thuringia|BOLD:AAL0103
Polistes nimpha|BC ZSM HYM 24035|Female|Italy|BOLD:AAL0103
Polistes nimpha|BC ZSM HYM 24661|Germany.Brandenburg|BOLD:AAL0103
Polistes nimpha|BC ZSM HYM 14407|Female|Germany.Brandenburg|BOLD:AAL0103
Polistes nimpha|BC ZSM HYM 21991|Female|Germany.Saxony-Anhalt|BOLD:AAL0103
Polistes nimpha|BC ZSM HYM 14012|Female|Germany.Baden-Wuerttemberg|BOLD:AAL0103
Polistes nimpha|BC ZSM HYM 19397|Female|Germany.Baden-Wuerttemberg|BOLD:AAL0103
Polistes nimpha|BC ZSM HYM 19398|Female|Germany.Baden-Wuerttemberg|BOLD:AAL0103
Polistes nimpha|BC ZSM HYM 04999|Female|Germany.Baden-Wuerttemberg|
Polistes nimpha|BC ZSM HYM 21990|Female|Germany.Saxony-Anhalt|BOLD:AAL0103
Polistes nimpha|BC ZSM HYM 19399|Female|Germany.Baden-Wuerttemberg|BOLD:AAL0103
Polistes nimpha|BC ZSM HYM 24659|Italy.Veneto|BOLD:AAL0103
Polistes nimpha|BC ZSM HYM 10207|Female|Italy.Lombardy|BOLD:AAL0103
Polistes nimpha|BC ZSM HYM 22716|Female|Greece|BOLD:AAL0103
Polistes nimpha|BC ZSM HYM 22717|Female|Greece|
Polistes nimpha|BC ZSM HYM 09881|Female|Germany.Brandenburg|
Polistes nimpha|BC ZSM HYM 05000|Female|Germany.Bavaria|
Polistes nimpha|BC ZSM HYM 14013|Female|Germany.Thuringia|BOLD:ACC1661
Polistes nimpha|BC ZSM HYM 14018|Female|Germany.Brandenburg|BOLD:ACC1661
Polistes nimpha|BC ZSM HYM 14017|Female|Germany.Thuringia|BOLD:ACC1661
Polistes nimpha|BC ZSM HYM 14014|Female|Germany.Baden-Wuerttemberg|BOLD:ACC1661
Polistes bucharensis|GBOL18675|Female|Azerbaijan|BOLD:ACR2719
Polistes bucharensis|GBOL18674|Male|Azerbaijan|BOLD:ACM7975
Polistes bucharensis|GBOL18676|Male|Azerbaijan|BOLD:ACM7975
Polistes bucharensis|BC ZSM HYM 24985|Female|Greece.Crete|BOLD:ACY7463
Polistes bucharensis|BC ZSM HYM 24644|Female|Greece.Crete|BOLD:ACY7463
Polistes bucharensis|BC ZSM HYM 24642|Male|Greece.Crete|BOLD:ACY7463
Polistes bucharensis|BC ZSM HYM 20056|Male|Cyprus|BOLD:ACM7975
Polistes bucharensis|BC ZSM HYM 21004|Female|Cyprus|BOLD:ACM7975
Polistes bucharensis|BC ZSM HYM 21002|Female|Cyprus|BOLD:ACM7975
Polistes bucharensis|BC ZSM HYM 20057|Female|Cyprus|BOLD:ACM7975
Polistes bucharensis|BC ZSM HYM 20050|Male|Cyprus|BOLD:ACM7975
Polistes bucharensis|BC ZSM HYM 21003|Female|Cyprus|BOLD:ACM7975
Polistes bucharensis|BC ZSM HYM 20049|Female|Cyprus|BOLD:ACM7975
Polistes dominula|GBOL18677|Female|Azerbaijan|BOLD:AAB7105
Polistes dominula|BC ZSM HYM 19386|Female|Germany.Baden-Wuerttemberg|BOLD:AAB7105
Polistes dominula|BC ZSM HYM 19394|Male|Germany.Baden-Wuerttemberg|BOLD:AAB7105
Polistes dominula|GBOL18936|Female|Greece|BOLD:AAB7105
Polistes dominula|GBOL18938|Female|Greece|BOLD:AAB7105
Polistes dominula|BC ZSM HYM 19385|Female|Germany.Baden-Wuerttemberg|BOLD:AAB7105
Polistes dominula|BC ZSM HYM 05001|Male|Germany.Baden-Wuerttemberg|BOLD:AAB7105
Polistes dominula|BC ZSM HYM 19392|Male|Germany.Baden-Wuerttemberg|BOLD:AAB7105
Polistes dominula|BC ZSM HYM 22047|Female|Croatia|BOLD:AAB7105
Polistes dominula|BC ZSM HYM 19381|Female|Italy.Aosta Valley|BOLD:AAB7105
Polistes dominula|BC ZSM HYM 19384|Male|Italy.Aosta Valley|BOLD:AAB7105
Polistes dominula|BC ZSM HYM 19383|Male|Italy.Aosta Valley|BOLD:AAB7105
Polistes dominula|BC ZSM HYM 19382|Female|Italy.Aosta Valley|BOLD:AAB7105
Polistes dominula|BC ZSM HYM 15509|Female|Germany.Saxony-Anhalt|BOLD:AAB7105
Polistes dominula|BC ZSM HYM 15505|Female|Germany.Saxony-Anhalt|BOLD:AAB7105
Polistes dominula|BC ZSM HYM 19395|Male|Germany.Baden-Wuerttemberg|BOLD:AAB7105
Polistes dominula|BC ZSM HYM 24648|Italy.Veneto|BOLD:AAB7105
Polistes dominula|BC ZSM HYM 19388|Female|Germany.Baden-Wuerttemberg|BOLD:AAB7105
Polistes dominula|BC ZSM HYM 10204|Female|Italy.Lombardy|BOLD:AAB7105
Polistes dominula|BC ZSM HYM 10201|Male|Italy.Veneto|BOLD:AAB7105
Polistes dominula|BC ZSM HYM 19390|Male|Germany.Baden-Wuerttemberg|BOLD:AAB7105
Polistes dominula|BC ZSM HYM 10205|Female|Germany.Baden-Wuerttemberg|BOLD:AAB7105
Polistes dominula|BC ZSM HYM 19393|Male|Germany.Baden-Wuerttemberg|BOLD:AAB7105
Polistes dominula|BC ZSM HYM 10203|Male|Italy.Veneto|BOLD:AAB7105
Polistes dominula|BC ZSM HYM 15507|Female|Germany.Saxony-Anhalt|BOLD:AAB7105
Polistes dominula|BC ZSM HYM 08258|Female|Germany.Baden-Wuerttemberg|BOLD:AAB7105
Polistes dominula|BC ZSM HYM 19391|Male|Germany.Baden-Wuerttemberg|BOLD:AAB7105
Polistes dominula|BC ZSM HYM 22046|Female|Croatia|BOLD:AAB7105
Polistes dominula|BC ZSM HYM 22344|Female|Switzerland.Graubunden|BOLD:AAB7105
Polistes dominula|BC ZSM HYM 24653|Italy.Veneto|BOLD:AAB7105
Polistes dominula|BC ZSM HYM 19387|Female|Germany.Baden-Wuerttemberg|BOLD:AAB7105
Polistes dominula|BC ZSM HYM 24646|Italy.Veneto|BOLD:AAB7105
Polistes dominula|BC ZSM HYM 05002|Male|Germany.Baden-Wuerttemberg|BOLD:AAB7105
Polistes dominula|BC ZSM HYM 10202|Female|Italy.Lombardy|BOLD:AAB7105
Polistes dominula|BC ZSM HYM 24647|Italy.Veneto|BOLD:AAB7105
Polistes dominula|BC ZSM HYM 05004|Female|Germany.Bavaria|BOLD:AAB7105
Polistes dominula|BC ZSM HYM 22045|Female|Croatia|BOLD:AAB7105
Polistes dominula|BC ZSM HYM 24650|Germany.Thuringia|BOLD:AAA9495
Polistes dominula|BC ZSM HYM 19389|Male|Germany.Baden-Wuerttemberg|BOLD:AAA9495
Polistes dominula|BC ZSM HYM 24660|Germany.Brandenburg|BOLD:AAA9495
Polistes dominula|BC ZSM HYM 05003|Female|France.Provence-Alpes-Cote d'Azur|BOLD:AAA9495

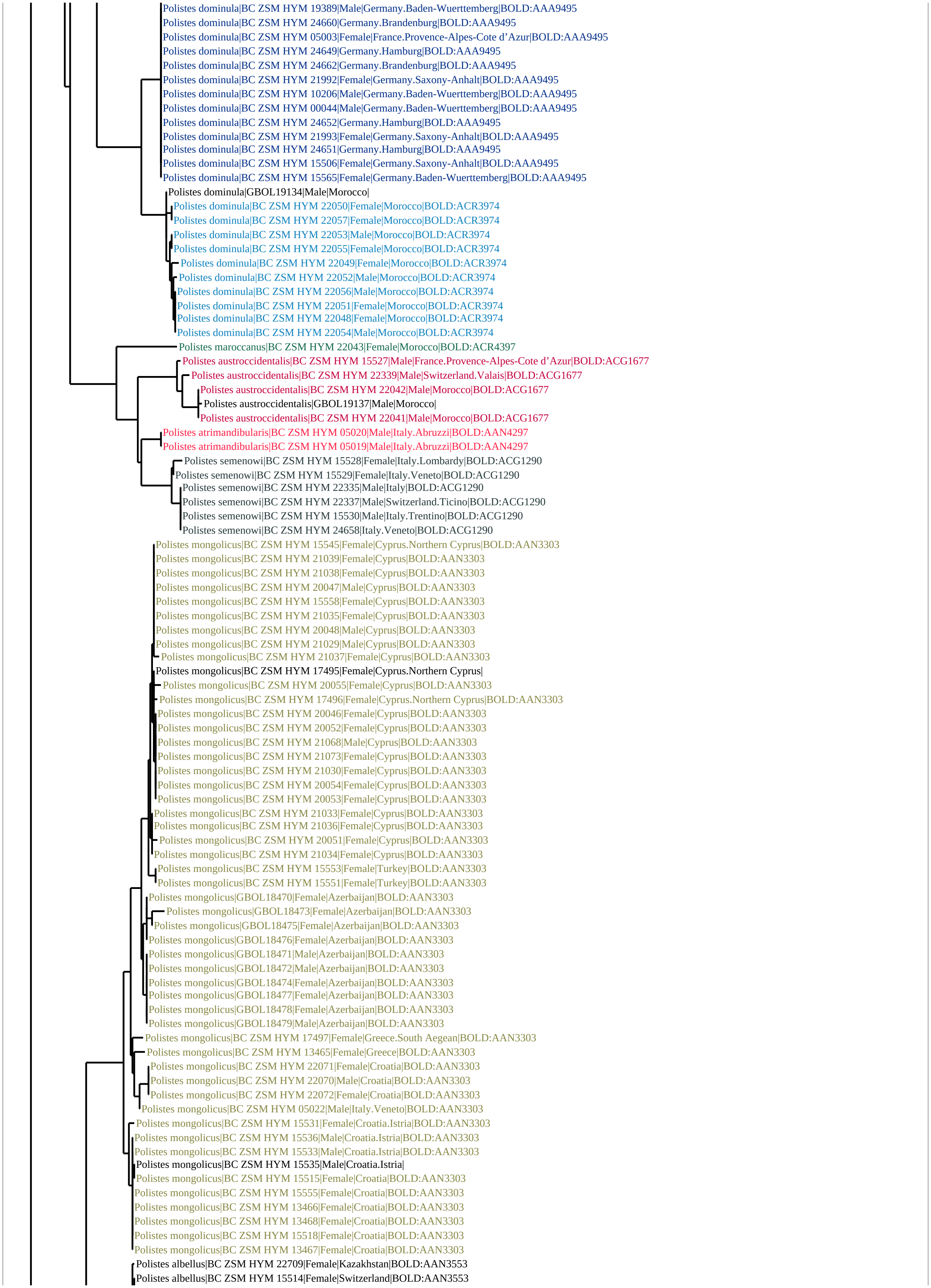
Polistes dominula|BC ZSM HYM 19389|Male|Germany.Baden-Wuerttemberg|BOLD:AAA9495
Polistes dominula|BC ZSM HYM 24660|Germany.Brandenburg|BOLD:AAA9495
Polistes dominula|BC ZSM HYM 05003|Female|France.Provence-Alpes-Cote d'Azur|BOLD:AAA9495
Polistes dominula|BC ZSM HYM 24649|Germany.Hamburg|BOLD:AAA9495
Polistes dominula|BC ZSM HYM 24662|Germany.Brandenburg|BOLD:AAA9495
Polistes dominula|BC ZSM HYM 21992|Female|Germany.Saxony-Anhalt|BOLD:AAA9495
Polistes dominula|BC ZSM HYM 10206|Male|Germany.Baden-Wuerttemberg|BOLD:AAA9495
Polistes dominula|BC ZSM HYM 00044|Male|Germany.Baden-Wuerttemberg|BOLD:AAA9495
Polistes dominula|BC ZSM HYM 24652|Germany.Hamburg|BOLD:AAA9495
Polistes dominula|BC ZSM HYM 21993|Female|Germany.Saxony-Anhalt|BOLD:AAA9495
Polistes dominula|BC ZSM HYM 24651|Germany.Hamburg|BOLD:AAA9495
Polistes dominula|BC ZSM HYM 15506|Female|Germany.Saxony-Anhalt|BOLD:AAA9495
Polistes dominula|BC ZSM HYM 15565|Female|Germany.Baden-Wuerttemberg|BOLD:AAA9495
Polistes dominula|GBOL19134|Male|Morocco|
Polistes dominula|BC ZSM HYM 22050|Female|Morocco|BOLD:ACR3974
Polistes dominula|BC ZSM HYM 22057|Female|Morocco|BOLD:ACR3974
Polistes dominula|BC ZSM HYM 22053|Male|Morocco|BOLD:ACR3974
Polistes dominula|BC ZSM HYM 22055|Female|Morocco|BOLD:ACR3974
Polistes dominula|BC ZSM HYM 22049|Female|Morocco|BOLD:ACR3974
Polistes dominula|BC ZSM HYM 22052|Male|Morocco|BOLD:ACR3974
Polistes dominula|BC ZSM HYM 22056|Male|Morocco|BOLD:ACR3974
Polistes dominula|BC ZSM HYM 22051|Female|Morocco|BOLD:ACR3974
Polistes dominula|BC ZSM HYM 22048|Female|Morocco|BOLD:ACR3974
Polistes dominula|BC ZSM HYM 22054|Male|Morocco|BOLD:ACR3974
Polistes maroccanus|BC ZSM HYM 22043|Female|Morocco|BOLD:ACR4397
Polistes austroccidentalis|BC ZSM HYM 15527|Male|France.Provence-Alpes-Cote d'Azur|BOLD:ACG1677
Polistes austroccidentalis|BC ZSM HYM 22339|Male|Switzerland.Valais|BOLD:ACG1677
Polistes austroccidentalis|BC ZSM HYM 22042|Male|Morocco|BOLD:ACG1677
Polistes austroccidentalis|GBOL19137|Male|Morocco|
Polistes austroccidentalis|BC ZSM HYM 22041|Male|Morocco|BOLD:ACG1677
Polistes atrimandibularis|BC ZSM HYM 05020|Male|Italy.Abruzzi|BOLD:AAN4297
Polistes atrimandibularis|BC ZSM HYM 05019|Male|Italy.Abruzzi|BOLD:AAN4297
Polistes semenowi|BC ZSM HYM 15528|Female|Italy.Lombardy|BOLD:ACG1290
Polistes semenowi|BC ZSM HYM 15529|Female|Italy.Veneto|BOLD:ACG1290
Polistes semenowi|BC ZSM HYM 22335|Male|Italy|BOLD:ACG1290
Polistes semenowi|BC ZSM HYM 22337|Male|Switzerland.Ticino|BOLD:ACG1290
Polistes semenowi|BC ZSM HYM 15530|Male|Italy.Trentino|BOLD:ACG1290
Polistes semenowi|BC ZSM HYM 24658|Italy.Veneto|BOLD:ACG1290
Polistes mongolicus|BC ZSM HYM 15545|Female|Cyprus.Northern Cyprus|BOLD:AAN3303
Polistes mongolicus|BC ZSM HYM 21039|Female|Cyprus|BOLD:AAN3303
Polistes mongolicus|BC ZSM HYM 21038|Female|Cyprus|BOLD:AAN3303
Polistes mongolicus|BC ZSM HYM 20047|Male|Cyprus|BOLD:AAN3303
Polistes mongolicus|BC ZSM HYM 15558|Female|Cyprus|BOLD:AAN3303
Polistes mongolicus|BC ZSM HYM 21035|Female|Cyprus|BOLD:AAN3303
Polistes mongolicus|BC ZSM HYM 20048|Male|Cyprus|BOLD:AAN3303
Polistes mongolicus|BC ZSM HYM 21029|Male|Cyprus|BOLD:AAN3303
Polistes mongolicus|BC ZSM HYM 21037|Female|Cyprus|BOLD:AAN3303
Polistes mongolicus|BC ZSM HYM 17495|Female|Cyprus.Northern Cyprus|
Polistes mongolicus|BC ZSM HYM 20055|Female|Cyprus|BOLD:AAN3303
Polistes mongolicus|BC ZSM HYM 17496|Female|Cyprus.Northern Cyprus|BOLD:AAN3303
Polistes mongolicus|BC ZSM HYM 20046|Female|Cyprus|BOLD:AAN3303
Polistes mongolicus|BC ZSM HYM 20052|Female|Cyprus|BOLD:AAN3303
Polistes mongolicus|BC ZSM HYM 21068|Male|Cyprus|BOLD:AAN3303
Polistes mongolicus|BC ZSM HYM 21073|Female|Cyprus|BOLD:AAN3303
Polistes mongolicus|BC ZSM HYM 21030|Female|Cyprus|BOLD:AAN3303
Polistes mongolicus|BC ZSM HYM 20054|Female|Cyprus|BOLD:AAN3303
Polistes mongolicus|BC ZSM HYM 20053|Female|Cyprus|BOLD:AAN3303
Polistes mongolicus|BC ZSM HYM 21033|Female|Cyprus|BOLD:AAN3303
Polistes mongolicus|BC ZSM HYM 21036|Female|Cyprus|BOLD:AAN3303
Polistes mongolicus|BC ZSM HYM 20051|Female|Cyprus|BOLD:AAN3303
Polistes mongolicus|BC ZSM HYM 21034|Female|Cyprus|BOLD:AAN3303
Polistes mongolicus|BC ZSM HYM 15553|Female|Turkey|BOLD:AAN3303
Polistes mongolicus|BC ZSM HYM 15551|Female|Turkey|BOLD:AAN3303
Polistes mongolicus|GBOL18470|Female|Azerbaijan|BOLD:AAN3303
Polistes mongolicus|GBOL18473|Female|Azerbaijan|BOLD:AAN3303
Polistes mongolicus|GBOL18475|Female|Azerbaijan|BOLD:AAN3303
Polistes mongolicus|GBOL18476|Female|Azerbaijan|BOLD:AAN3303
Polistes mongolicus|GBOL18471|Male|Azerbaijan|BOLD:AAN3303
Polistes mongolicus|GBOL18472|Male|Azerbaijan|BOLD:AAN3303
Polistes mongolicus|GBOL18474|Female|Azerbaijan|BOLD:AAN3303
Polistes mongolicus|GBOL18477|Female|Azerbaijan|BOLD:AAN3303
Polistes mongolicus|GBOL18478|Female|Azerbaijan|BOLD:AAN3303
Polistes mongolicus|GBOL18479|Male|Azerbaijan|BOLD:AAN3303
Polistes mongolicus|BC ZSM HYM 17497|Female|Greece.South Aegean|BOLD:AAN3303
Polistes mongolicus|BC ZSM HYM 13465|Female|Greece|BOLD:AAN3303
Polistes mongolicus|BC ZSM HYM 22071|Female|Croatia|BOLD:AAN3303
Polistes mongolicus|BC ZSM HYM 22070|Male|Croatia|BOLD:AAN3303
Polistes mongolicus|BC ZSM HYM 22072|Female|Croatia|BOLD:AAN3303
Polistes mongolicus|BC ZSM HYM 05022|Male|Italy.Veneto|BOLD:AAN3303
Polistes mongolicus|BC ZSM HYM 15531|Female|Croatia.Istria|BOLD:AAN3303
Polistes mongolicus|BC ZSM HYM 15536|Male|Croatia.Istria|BOLD:AAN3303
Polistes mongolicus|BC ZSM HYM 15533|Male|Croatia.Istria|BOLD:AAN3303
Polistes mongolicus|BC ZSM HYM 15535|Male|Croatia.Istria|
Polistes mongolicus|BC ZSM HYM 15515|Female|Croatia|BOLD:AAN3303
Polistes mongolicus|BC ZSM HYM 15555|Female|Croatia|BOLD:AAN3303
Polistes mongolicus|BC ZSM HYM 13466|Female|Croatia|BOLD:AAN3303
Polistes mongolicus|BC ZSM HYM 13468|Female|Croatia|BOLD:AAN3303
Polistes mongolicus|BC ZSM HYM 15518|Female|Croatia|BOLD:AAN3303
Polistes mongolicus|BC ZSM HYM 13467|Female|Croatia|BOLD:AAN3303
Polistes albellus|BC ZSM HYM 22709|Female|Kazakhstan|BOLD:AAN3553
Polistes albellus|BC ZSM HYM 15514|Female|Switzerland|BOLD:AAN3553

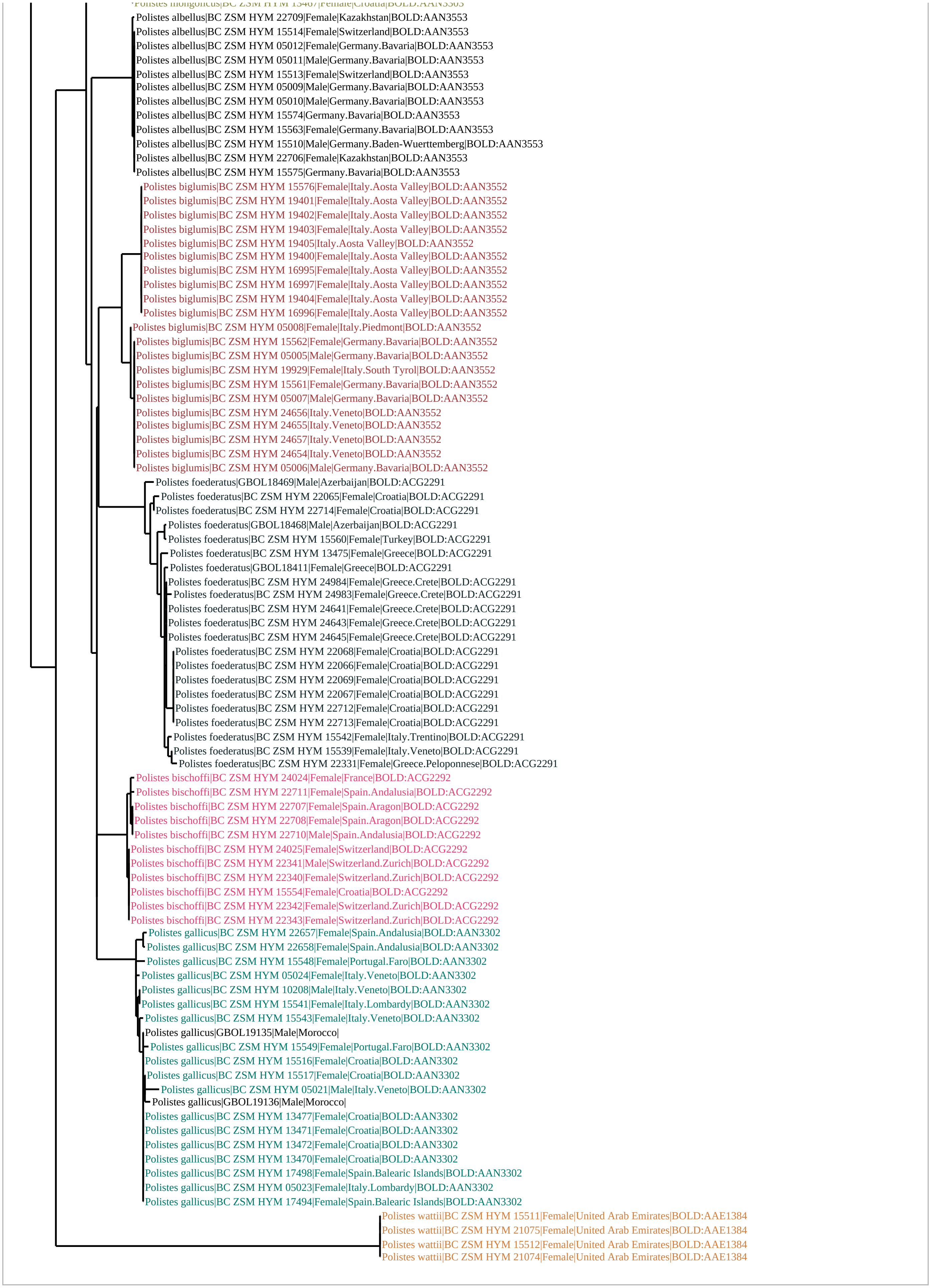
Polistes mongolicus|BC ZSM HYM 13467|Female|Croatia|BOLD:AAN[illegible]
Polistes albellus|BC ZSM HYM 22709|Female|Kazakhstan|BOLD:AAN3553
Polistes albellus|BC ZSM HYM 15514|Female|Switzerland|BOLD:AAN3553
Polistes albellus|BC ZSM HYM 05012|Female|Germany.Bavaria|BOLD:AAN3553
Polistes albellus|BC ZSM HYM 05011|Male|Germany.Bavaria|BOLD:AAN3553
Polistes albellus|BC ZSM HYM 15513|Female|Switzerland|BOLD:AAN3553
Polistes albellus|BC ZSM HYM 05009|Male|Germany.Bavaria|BOLD:AAN3553
Polistes albellus|BC ZSM HYM 05010|Male|Germany.Bavaria|BOLD:AAN3553
Polistes albellus|BC ZSM HYM 15574|Germany.Bavaria|BOLD:AAN3553
Polistes albellus|BC ZSM HYM 15563|Female|Germany.Bavaria|BOLD:AAN3553
Polistes albellus|BC ZSM HYM 15510|Male|Germany.Baden-Wuerttemberg|BOLD:AAN3553
Polistes albellus|BC ZSM HYM 22706|Female|Kazakhstan|BOLD:AAN3553
Polistes albellus|BC ZSM HYM 15575|Germany.Bavaria|BOLD:AAN3553
Polistes biglumis|BC ZSM HYM 15576|Female|Italy.Aosta Valley|BOLD:AAN3552
Polistes biglumis|BC ZSM HYM 19401|Female|Italy.Aosta Valley|BOLD:AAN3552
Polistes biglumis|BC ZSM HYM 19402|Female|Italy.Aosta Valley|BOLD:AAN3552
Polistes biglumis|BC ZSM HYM 19403|Female|Italy.Aosta Valley|BOLD:AAN3552
Polistes biglumis|BC ZSM HYM 19405|Italy.Aosta Valley|BOLD:AAN3552
Polistes biglumis|BC ZSM HYM 19400|Female|Italy.Aosta Valley|BOLD:AAN3552
Polistes biglumis|BC ZSM HYM 16995|Female|Italy.Aosta Valley|BOLD:AAN3552
Polistes biglumis|BC ZSM HYM 16997|Female|Italy.Aosta Valley|BOLD:AAN3552
Polistes biglumis|BC ZSM HYM 19404|Female|Italy.Aosta Valley|BOLD:AAN3552
Polistes biglumis|BC ZSM HYM 16996|Female|Italy.Aosta Valley|BOLD:AAN3552
Polistes biglumis|BC ZSM HYM 05008|Female|Italy.Piedmont|BOLD:AAN3552
Polistes biglumis|BC ZSM HYM 15562|Female|Germany.Bavaria|BOLD:AAN3552
Polistes biglumis|BC ZSM HYM 05005|Male|Germany.Bavaria|BOLD:AAN3552
Polistes biglumis|BC ZSM HYM 19929|Female|Italy.South Tyrol|BOLD:AAN3552
Polistes biglumis|BC ZSM HYM 15561|Female|Germany.Bavaria|BOLD:AAN3552
Polistes biglumis|BC ZSM HYM 05007|Male|Germany.Bavaria|BOLD:AAN3552
Polistes biglumis|BC ZSM HYM 24656|Italy.Veneto|BOLD:AAN3552
Polistes biglumis|BC ZSM HYM 24655|Italy.Veneto|BOLD:AAN3552
Polistes biglumis|BC ZSM HYM 24657|Italy.Veneto|BOLD:AAN3552
Polistes biglumis|BC ZSM HYM 24654|Italy.Veneto|BOLD:AAN3552
Polistes biglumis|BC ZSM HYM 05006|Male|Germany.Bavaria|BOLD:AAN3552
Polistes foederatus|GBOL18469|Male|Azerbaijan|BOLD:ACG2291
Polistes foederatus|BC ZSM HYM 22065|Female|Croatia|BOLD:ACG2291
Polistes foederatus|BC ZSM HYM 22714|Female|Croatia|BOLD:ACG2291
Polistes foederatus|GBOL18468|Male|Azerbaijan|BOLD:ACG2291
Polistes foederatus|BC ZSM HYM 15560|Female|Turkey|BOLD:ACG2291
Polistes foederatus|BC ZSM HYM 13475|Female|Greece|BOLD:ACG2291
Polistes foederatus|GBOL18411|Female|Greece|BOLD:ACG2291
Polistes foederatus|BC ZSM HYM 24984|Female|Greece.Crete|BOLD:ACG2291
Polistes foederatus|BC ZSM HYM 24983|Female|Greece.Crete|BOLD:ACG2291
Polistes foederatus|BC ZSM HYM 24641|Female|Greece.Crete|BOLD:ACG2291
Polistes foederatus|BC ZSM HYM 24643|Female|Greece.Crete|BOLD:ACG2291
Polistes foederatus|BC ZSM HYM 24645|Female|Greece.Crete|BOLD:ACG2291
Polistes foederatus|BC ZSM HYM 22068|Female|Croatia|BOLD:ACG2291
Polistes foederatus|BC ZSM HYM 22066|Female|Croatia|BOLD:ACG2291
Polistes foederatus|BC ZSM HYM 22069|Female|Croatia|BOLD:ACG2291
Polistes foederatus|BC ZSM HYM 22067|Female|Croatia|BOLD:ACG2291
Polistes foederatus|BC ZSM HYM 22712|Female|Croatia|BOLD:ACG2291
Polistes foederatus|BC ZSM HYM 22713|Female|Croatia|BOLD:ACG2291
Polistes foederatus|BC ZSM HYM 15542|Female|Italy.Trentino|BOLD:ACG2291
Polistes foederatus|BC ZSM HYM 15539|Female|Italy.Veneto|BOLD:ACG2291
Polistes foederatus|BC ZSM HYM 22331|Female|Greece.Peloponnese|BOLD:ACG2291
Polistes bischoffi|BC ZSM HYM 24024|Female|France|BOLD:ACG2292
Polistes bischoffi|BC ZSM HYM 22711|Female|Spain.Andalusia|BOLD:ACG2292
Polistes bischoffi|BC ZSM HYM 22707|Female|Spain.Aragon|BOLD:ACG2292
Polistes bischoffi|BC ZSM HYM 22708|Female|Spain.Aragon|BOLD:ACG2292
Polistes bischoffi|BC ZSM HYM 22710|Male|Spain.Andalusia|BOLD:ACG2292
Polistes bischoffi|BC ZSM HYM 24025|Female|Switzerland|BOLD:ACG2292
Polistes bischoffi|BC ZSM HYM 22341|Male|Switzerland.Zurich|BOLD:ACG2292
Polistes bischoffi|BC ZSM HYM 22340|Female|Switzerland.Zurich|BOLD:ACG2292
Polistes bischoffi|BC ZSM HYM 15554|Female|Croatia|BOLD:ACG2292
Polistes bischoffi|BC ZSM HYM 22342|Female|Switzerland.Zurich|BOLD:ACG2292
Polistes bischoffi|BC ZSM HYM 22343|Female|Switzerland.Zurich|BOLD:ACG2292
Polistes gallicus|BC ZSM HYM 22657|Female|Spain.Andalusia|BOLD:AAN3302
Polistes gallicus|BC ZSM HYM 22658|Female|Spain.Andalusia|BOLD:AAN3302
Polistes gallicus|BC ZSM HYM 15548|Female|Portugal.Faro|BOLD:AAN3302
Polistes gallicus|BC ZSM HYM 05024|Female|Italy.Veneto|BOLD:AAN3302
Polistes gallicus|BC ZSM HYM 10208|Male|Italy.Veneto|BOLD:AAN3302
Polistes gallicus|BC ZSM HYM 15541|Female|Italy.Lombardy|BOLD:AAN3302
Polistes gallicus|BC ZSM HYM 15543|Female|Italy.Veneto|BOLD:AAN3302
Polistes gallicus|GBOL19135|Male|Morocco|
Polistes gallicus|BC ZSM HYM 15549|Female|Portugal.Faro|BOLD:AAN3302
Polistes gallicus|BC ZSM HYM 15516|Female|Croatia|BOLD:AAN3302
Polistes gallicus|BC ZSM HYM 15517|Female|Croatia|BOLD:AAN3302
Polistes gallicus|BC ZSM HYM 05021|Male|Italy.Veneto|BOLD:AAN3302
Polistes gallicus|GBOL19136|Male|Morocco|
Polistes gallicus|BC ZSM HYM 13477|Female|Croatia|BOLD:AAN3302
Polistes gallicus|BC ZSM HYM 13471|Female|Croatia|BOLD:AAN3302
Polistes gallicus|BC ZSM HYM 13472|Female|Croatia|BOLD:AAN3302
Polistes gallicus|BC ZSM HYM 13470|Female|Croatia|BOLD:AAN3302
Polistes gallicus|BC ZSM HYM 17498|Female|Spain.Balearic Islands|BOLD:AAN3302
Polistes gallicus|BC ZSM HYM 05023|Female|Italy.Lombardy|BOLD:AAN3302
Polistes gallicus|BC ZSM HYM 17494|Female|Spain.Balearic Islands|BOLD:AAN3302
Polistes wattii|BC ZSM HYM 15511|Female|United Arab Emirates|BOLD:AAE1384
Polistes wattii|BC ZSM HYM 21075|Female|United Arab Emirates|BOLD:AAE1384
Polistes wattii|BC ZSM HYM 15512|Female|United Arab Emirates|BOLD:AAE1384
Polistes wattii|BC ZSM HYM 21074|Female|United Arab Emirates|BOLD:AAE1384
